# Chiral auxiliary recycling in continuous flow: automated recovery and reuse of Oppolzer's sultam†
†Electronic supplementary information (ESI) available: Experimental details, details of flow reactor and equipment, characterization data of compounds. See DOI: 10.1039/c7sc05192a


**DOI:** 10.1039/c7sc05192a

**Published:** 2018-01-25

**Authors:** R. J. Sullivan, S. G. Newman

**Affiliations:** a Centre for Catalysis Research and Innovation , Department of Chemistry and Biomolecular Sciences , University of Ottawa , 10 Marie-Curie , Ottawa , Ontario K1N 6N5A , Canada . Email: stephen.newman@uottawa.ca

## Abstract

The telescoping of a three-stage, chiral auxiliary-mediated transformation in flow is described, including continuous separation of the product and auxiliary. The auxiliary can either be collected for later reuse, or directly fed back to the beginning of the process for recycling in real time, enabling each molecule of auxiliary to make multiple equivalents of chiral product and thus minimizing the step- and atom-economy issues associated with auxiliary-mediated synthesis. This concept is demonstrated for the asymmetric hydrogenation of olefins using Oppolzer's sultam, shortening the total reaction time >100 fold compared to batch, and demonstrating formal sub-stoichiometric auxiliary loading with respect to the process by automating auxiliary recycling within a closed loop.

## Abstract 2

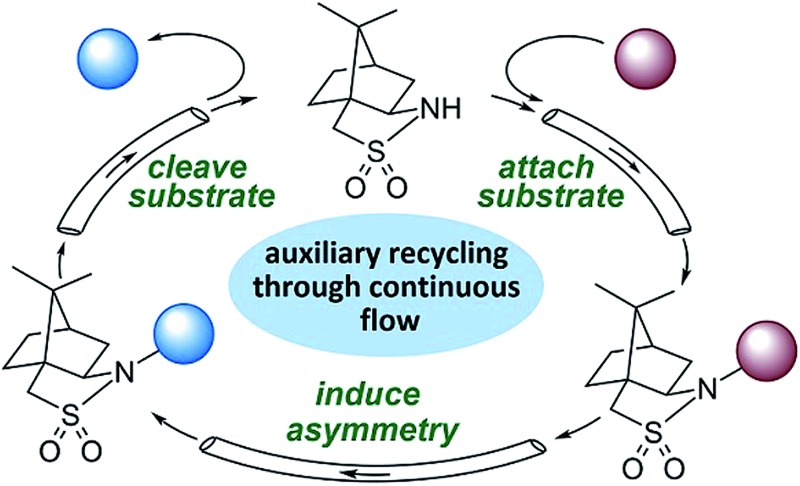
The telescoping of a three-stage, chiral auxiliary-mediated transformation in flow is described, including continuous separation of the product and auxiliary, enabling automated auxiliary reuse.

## Introduction

Accessing enantiopure molecules remains one of the toughest challenges in synthetic organic chemistry. The use of catalysis to preferentially prepare a single enantiomer from achiral starting material is generally seen as the most efficient and elegant method to do so. In contrast, the use of chiral auxiliaries, wherein an optically pure material is covalently linked to a substrate to transfer chiral information *via* a subsequent diastereoselective reaction, is seen as wasteful. Such strategies necessitate multiple chemical steps to install, utilize, and cleave the auxiliary, which is required in stoichiometric quantities. Nonetheless, chiral auxiliaries remain a core tool for asymmetric synthesis due to their ability to enable predictable, robust, highly stereoselective transformations, in many instances for reactions where catalytic methods do not exist or are inefficient.^1^,^2^ Because of this, chiral auxiliary methods are frequently carried out in the pharmaceutical industry for large scale synthesis of APIs.^3^

**Scheme 1:**
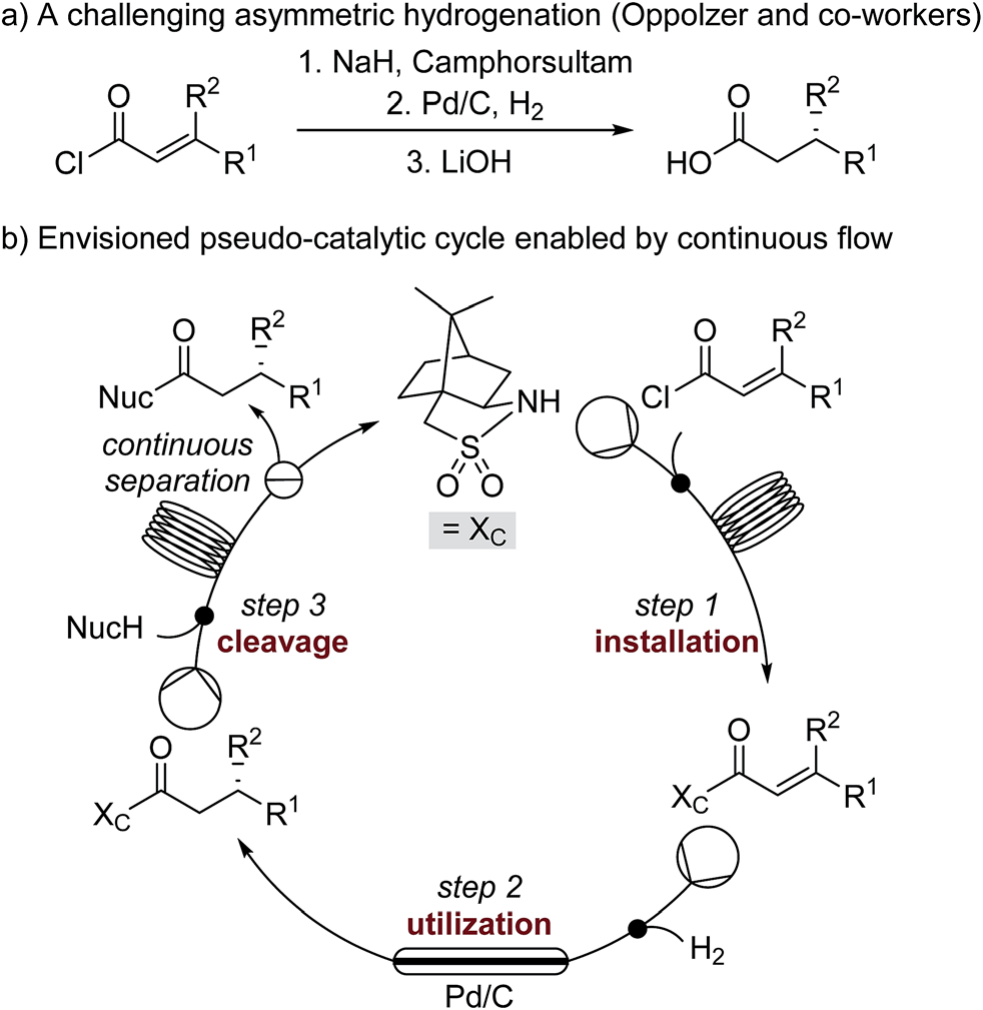
(a) A challenging asymmetric hydrogenation developed by Oppolzer and co-workers. (b) The envisioned pseudo-catalytic cycle. In contrast to a conventional catalytic cycle, wherein a series of reaction steps are separated by time, the flow pseudo-catalytic cycle can be realized because a series of continuous reaction steps are separated by space.

We hypothesized that the major downsides of chiral auxiliary-mediated synthesis could be minimized through the implementation of a continuous flow process. Flow technology is now common for increasing the efficiency and decreasing the environmental impact of chemical synthesis,^4^ for instance by through telescoping of multi-step procedures,^5^ though application to auxiliary mediated synthesis is limited.^6^ In the bulk chemical industry, further advantages are realized through automated separation and recycling of sub- to super-stoichiometric waste streams.^7^ Continuous recycling systems for complex molecule synthesis have thus far only been applied towards improving turnover numbers in catalytic reactions.^8^ Automated recycling of stoichiometric waste products, particularly in multi-step synthesis, can provide much higher reward. To demonstrate this, we chose to investigate the asymmetric hydrogenation developed by Oppolzer and co-workers (Scheme 1a).^9^ This procedure uses enantiopure camphorsultam (Oppolzer's Sultam) as a chiral auxiliary to control the diastereoselectivity in the reduction step prior to auxiliary cleavage, giving the final product in 95 : 5 to 98 : 2 enantiomeric ratio. Since this report in 1986, catalytic hydrogenations to access similar β-chiral acids and esters have been developed, for instance by the groups of Noyori,^10^ Buchwald,^11^ and Pfaltz;^12^ however, none have been able to achieve the generality and high enantiomeric ratios of the chiral auxiliary system when the two β-substituents are simple alkyl groups.^13^

A continuous flow process may enhance this procedure in two key ways. First, telescoping three separate steps into one automated process decreases the overall reaction time, number of physical manipulations, purifications, and waste generation. Second, efficient separation of the auxiliary from the product allows continuous auxiliary recycling, enabling multiple equivalents of chiral product to be produced for each molecule of auxiliary. This can be thought of as a pseudo-catalytic cycle in space, with each step of the cycle represented by a stoichiometric reaction and recovery/reuse of the auxiliary providing ‘turnover’ (Scheme 1b). Herein, we disclose how such a recycle system can be developed in the context of the sultam-mediated asymmetric hydrogenation by redesigning each step for continuous flow synthesis, linking them together in a telescoped process, and designing the process to enable automated auxiliary recovery and recycling.

## Results and discussion

At the outset of the investigation, challenges preventing direct translation of the established literature protocol into a telescoped flow process were identified. First, the literature procedure used a different solvent for each of the three reactions. Second, purification options are more limited in flow and use of continuous liquid/liquid extractions was desired for both inline purifications between steps and the eventual separation of the auxiliary and product at the end of the process (based on p*K*_a_ differences). Last, NaH was the base used for the reported sultam acylation reaction. This is incompatible with flow due to insolubility of both NaH and NaCl that precipitates during the reaction under the necessarily anhydrous conditions.

To address solvent-related challenges, it was decided to re-develop the three reactions in toluene, therefore removing the need for solvent switches between steps and introducing potential for liquid/liquid separations. Towards overcoming solid handling issues, flow-compatible reaction conditions were explored for the acylation step (Table 1). Substituting NaH with organic bases was unsatisfactory due to low conversions and precipitate formation (entry 2). Biphasic Schotten–Baumann conditions were efficient with phase transfer catalysis (PTC) in batch (entry 3),^14^ however, only moderate and highly variable yields were obtained in slug flow (entry 4). Incorporation of active mixing in flow returned yields to near quantitative (entry 5).^15^ A simple custom mixing unit was utilized composed of a hollowed syringe reactor with oscillating stir bars, inspired by a similar designed from Ley and co-workers, providing high mass transfer at low flow rates.^16^ For the post-reaction liquid/liquid separation, membrane based separators proved incompatible with the high pH aqueous phase and therefore gravity-based separation was used (for details see ESI†). Addition of 4% (w/w) NaCl to the 4% (w/w) NaOH aqueous phase was found to improve the separation.

**Table 1:** Design of a flow-compatible acylation process

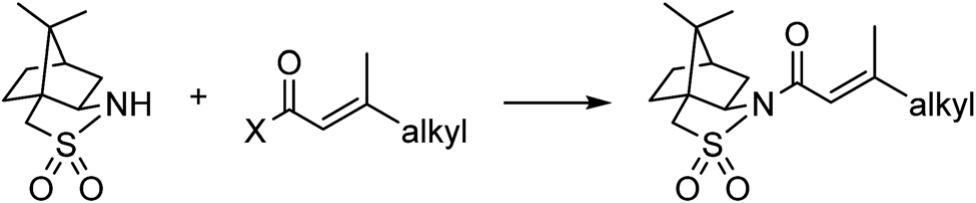				
Entry	X	Mode	Conditions	Results/comments
1	Cl	Batch	NaH^*a*^	Up to 84% (ref. 9)
2	Cl	Batch	Organic bases	Low conversion; solid formation
3	Cl	Batch	PTC, vigorous stirring, 10 min^*b*^	>95%
4	Cl	Flow	PTC, passive mixing, *t*_res_ = 10 min^*c*^	30–70%^*d*^
5	Cl	Flow	PTC, active mixing, *t*_res_ = 4.3 min^*e*^	>95%

^*a*^Sultam first treated with NaH prior to addition of acid chloride in toluene.

^*b*^1 mol% aliquat 336 added as a phase transfer catalyst, toluene/4% NaOH_(aq)_.^17^

^*c*^1 mol% aliquat 336 added as a phase transfer catalyst in a tubular plug flow reactor.

^*d*^Yields varied with time due to poor mixing.

^*e*^1 mol% aliquat 336 added as phase transfer catalyst using active mixing units.

The hydrogenation was next investigated using a packed bed reactor of Pd/C.^18^ Full conversion (*t*_res_ ≈ 1 min) was initially achieved but catalyst deactivation prevented long term operation. Hypothesizing that basic impurities were at fault,^19^ a co-feed of 0.1% (v/v) aqueous acetic acid was incorporated in combination with an increase of the reactor temperature to 45 °C.^20^ With these minor modifications, the acylation and hydrogenation reactions were successfully telescoped to yield 97% of hydrogenated substrate with no observable loss of catalyst activity over several hours of operation (eqn (1)). A 95 : 5 d.r. was obtained, consistent with the literature batch procedure that used EtOH as solvent with a 1.5 h reaction time. The process output could be carried through a modified Biotage Universal Phase Separator (Fig. S6†) to separate the excess hydrogen gas and aqueous co-feed, retaining the product in the organic phase.1
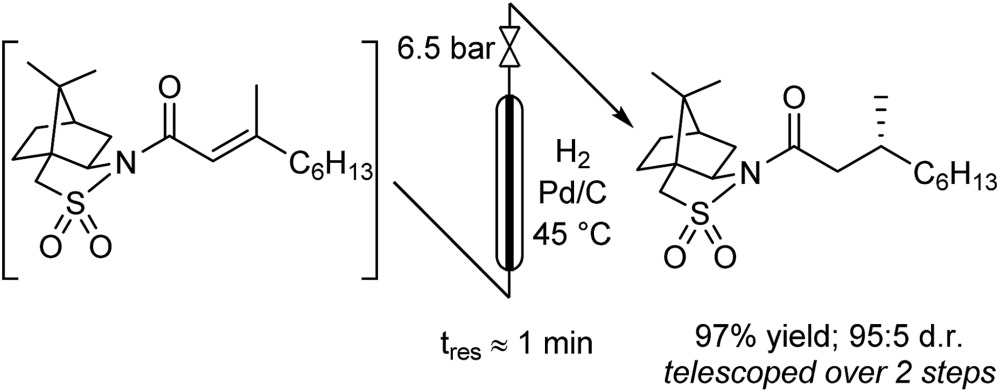



**Fig. 1:**
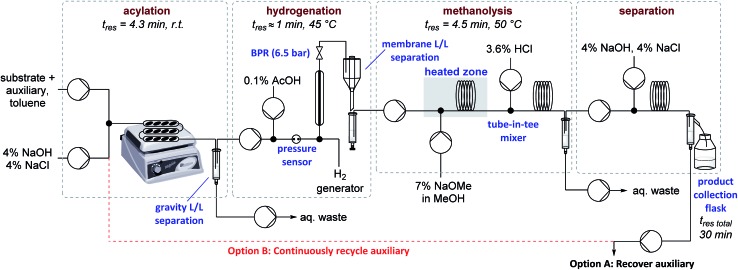
Schematic of flow reactor set up for the telescoped reaction sequence with or without automated recycling of the auxiliary. For auxiliary recovery experiments (Option A) 1.0 eq. of auxiliary w.r.t. substrate. For recycle experiments (Option B) 0.35 eq. of auxiliary w.r.t. substrate. L/L = liquid/liquid, BPR = back pressure regulator.

Auxiliary cleavage and separation from the product by selective deprotonation (carboxylic acid aq. p*K*_a_ ≈ 5, sultam aq. p*K*_a_ ≈ 11.5) and continuous liquid/liquid extraction was next examined (Table 2). Initial attempts to perform the hydrolysis using PTC appeared promising but were ultimately abandoned due to long reaction times and solid formation (entry 2). Introducing MeOH as a co-solvent to improve reaction homogeneity greatly improved reaction rates, but precipitate formation was still problematic (entry 3). Expecting that solubility problems stemmed from either salt formation or substrate/product decomposition, it was decided to target methanolysis instead of hydrolysis. Replacing KOH with NaOMe allowed milder conditions and shorter residence times without precipitate formation (entry 4). A tube-in-tee mixer after the methanolysis was necessary for effective mixing of the acid quench stream with the plugs of NaOMe. Methanolysis also greatly simplified the downstream product-auxiliary separation since the recovered auxiliary could be directly extracted from the organic phase. Overall yields of ∼70% could now be achieved over the three telescoped steps and 4 single-stage liquid/liquid extractions/separations, representing average yields/recoveries of ∼95% over each of these 7 operations. A schematic of the full experimental set up is shown in Fig. 1 and detailed descriptions are available in the ESI (Fig. S1–S8†). Total residence time from start to finish over all three stages was ∼30 min, including processing time through all pumps and the liquid/liquid separators used for in-line work up.

**Table 2:** Design of a flow-compatible auxiliary cleavage reaction

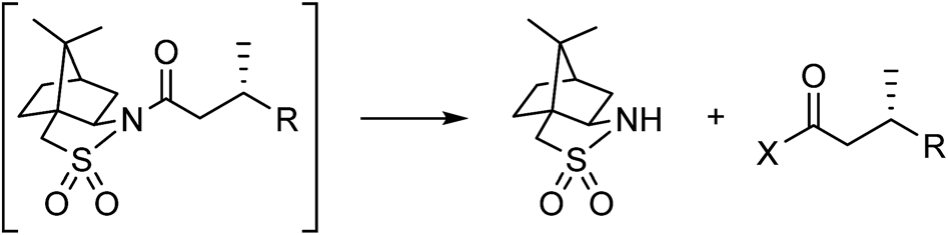				
Entry	X	Mode	Conditions	Result
1	OH	Batch	LiOH, 18 h	92% – quant. (ref. 9) *aux. not recovered*
2	OH	Batch	PTC, KOH^*a*^	Slow reaction; precipitate formation
3	OH	Flow	KOH, MeOH, 90 °C, 9 min	Fast reaction; precipitate formation
4	OMe	Flow	NaOMe, 50 °C, 4.5 min	∼70% yield Me ester *telescoped over 3 steps^*b*^*

^*a*^5 mol% 18-crown-6 and 5 mol% 2,5-dimethyl-2,5-hexanediol added as phase transfer catalysts.^21^

^*b*^Yields and auxiliary recovery varied depending on nature of alkyl group. See Table 3.

To determine if the process was general for different β,β-disubstituted α,β-unsaturated acid chlorides, a selection of substrates was examined in the multi-stage flow reactor (Table 3, top). In each case, the separated product and auxiliary effluents were collected for a 3 h period at steady state. The final ester products were obtained with isolated yields of 67–72%. Diastereoselectivity over the hydrogenation ranged from 92 : 8 to 98 : 2. In comparison to the 3-step batch process, this represented similar to greatly improved yields (batch yields over 3 steps: 45–76%) with greatly reduced processing times (batch reaction times: 2.5 h for acylation, 1.5 h for hydrogenation, 18 h for hydrolysis^9^,^22^) and equivalent diastereoselectivity. Moreover, due to the designed separation of the auxiliary and product in the three-step flow process, the auxiliary could be directly recovered in 71–79% crude yield and recrystallized to >99% purity (48–56% yield), which enabled reuse of the material for each subsequent substrate scope example.

**Table 3:** Substrate scope for auxiliary recovery and recycle experiments

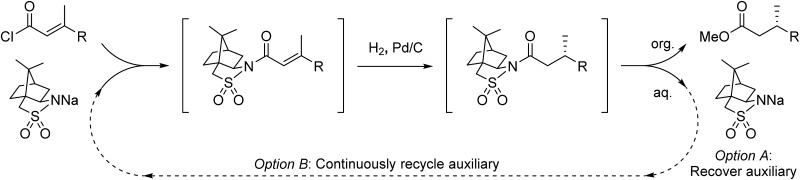				
	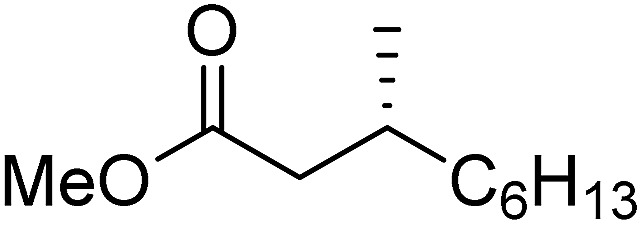	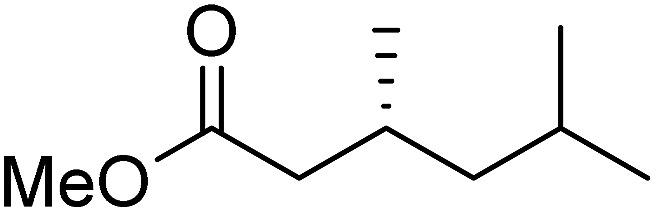	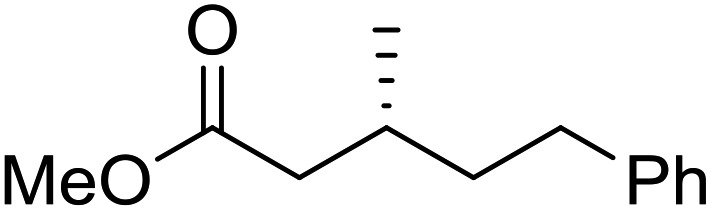	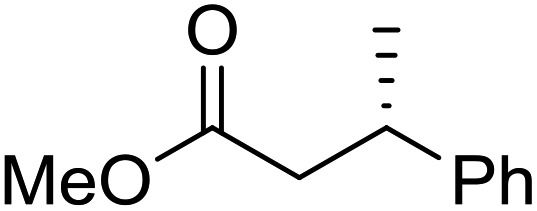
**A: auxiliary recovery experiments** ^*a*^				
Product yield (e.r.)	67% (95 : 5)	72% (98 : 2)	71% (93 : 7)	67% (92 : 8)
Recovered auxiliary^*b*^	79% (53%)	71% (54%)	73% (56%)	72% (48%)

**B: auxiliary recyle experiments** ^*c*^				
Product yield (e.r.)	54%^*d*^ (95 : 5)	48% (98 : 2)	57% (95 : 5)	68% (90 : 10)

^*a*^Product collected over 6 residence volumes (3 h; 3.6 mmol processed) at steady state. Isolated yields are reported. Enantiomeric ratios determined by monitoring *via* GC, the diastereomeric ratio of the intermediate after hydrogenation. See Fig. S9–S12.

^*b*^Crude yield (purified yield after recrystallization from hexanes).

^*c*^Product collected over 9 residence volumes (4.5 h; 5.4 mmol processed with 8 auxiliary recycles) at steady state. Isolated yields are reported.

^*d*^94% purity; contaminated with aldehyde by-product.

Focus was then turned towards automating the auxiliary recycling process (Fig. 1, dotted line). The same flow system was utilized with only two modifications. First, the amount of auxiliary in the substrate feed solution was decreased from 1 eq. to 0.35 eq. (the amount determined experimentally to be necessary to make up for lost auxiliary over the three transformations and four inline liquid/liquid extractions). Second, 0.65 eq. of auxiliary was dissolved in the 4% (w/w) NaOH, 4% (w/w) NaCl feed solution used for start-up, then after steady state was reached this feed was replaced by the extracted auxiliary stream exiting the process.

The same substrate scope was reevaluated with this modified process, operating continuously for 4.5 hours (∼8 auxiliary recycles; Table 3, bottom). In each case, transition to a recycling reactor went smoothly, with only slight losses in overall yield relative to the process without auxiliary recycling. This was attributed to a drop in extraction efficiency due to impurity carry-through with the recycle feed which could ultimately be addressed on scaleup with multi-stage extraction. Diastereomeric ratios remained constant over 4.5 h of steady state operation and were consistent with the d.r. values obtained in the previous experiments without auxiliary recycle.

## Conclusions

In summary, we have developed a continuous system to enable the auxiliary-mediated asymmetric hydrogenation of conjugated olefins to occur in a single telescoped process. The chiral auxiliary and chiral ester product are continuously separated, which allows the auxiliary to be recovered and subsequently reused. Alternatively, the auxiliary reuse can be automated in real time by sending the auxiliary recovery feed exiting the process back to the inlet of the first reaction stage. By closing this recycle loop, formal substoichiometric auxiliary loading is achieved with respect to the process. Average yields/recoveries ranged from 90–95% per reaction or in-line purification step, with a processing time of approximately 30 minutes per pass, giving a substantial improvement in efficiency while maintaining similar control in stereochemistry as compared to the batch procedure. To our knowledge, this is the first demonstration of flow chemistry-enabled recycling being used to render a stoichiometric reaction component ‘pseudo-catalytic’ in multi-step synthesis. Efforts are underway to generalize this system to a broader range of important auxiliary-mediated transformations that suffer from poor step- and atom-economy.

## Conflicts of interest

There are no conflicts to declare.

## Supplementary Material

Supplementary information
