# Supplementary material for: Chiral auxiliary recycling in continuous flow: automated recovery and reuse of Oppolzer's sultam
Source: Chem Sci. 2018 Jan 25;9(8):2130–4. doi: 10.1039/c7sc05192a (PMC5896371; doi:10.1039/c7sc05192a)
Supplement: Supplementary file 1 [file SC-009-C7SC05192A-s001.pdf]

Electronic Supplementary Material (ESI) for Chemical Science.
This journal is © The Royal Society of Chemistry 2018

Supporting Information for

Chiral Auxiliary Recycling in Continuous Flow: Automated Recovery and Reuse of Oppolzer's Sultam

Ryan J. Sullivan, Stephen G. Newman*

Centre for Catalysis Research and Innovation
Department of Chemistry and Biomolecular Sciences
University of Ottawa
10 Marie-Curie, Ottawa, Ontario, Canada, K1N 6N5.
*E-mail: stephen.newman@uottawa.ca

Contents

1 General experimental details 2

2 Details of flow reactor and equipment 2

3 Procedure for synthesis of substrates 11

4 Experimental details from optimization of reaction steps for flow 15

5 Procedure for telescoped flow synthesis of chiral materials 16

6 Spectra of substrates and products 20

7 Example GC traces of diastereomers following hydrogenation 44

8 Diastereoselective excess monitoring of hydrogenation 48

9 Chiral HPLC trace of (*R*)-3-Methyl-5-phenlybutanoic acid methyl ester 52

References 53

1 General experimental details

Unless otherwise noted reagents were used as received. Palladium on carbon was purchased from Strem, (10% palladium on activated carbon, reduced, dry powder, product 46-1900). All other chemicals were obtained from either Sigma Aldrich or Combi-Blocks. Aliquat 336 was absorbed onto silica gel, dry loaded onto a 40×100 mm silica column, eluted with DCM→95:5 DCM:MeOH, and evaporated prior to use.

^{1}H NMR and ^{13}C NMR were recorded on a Bruker AVANCE 400 MHz spectrometer and referenced to residual solvent signals. Melting point ranges were determined on a Canlab GallenKamp Melting Point Apparatus. GC yields for optimization studies were obtained via a 5-point calibration curve using FID analysis on an Agilent Technologies 7890B GC with 30 m × 0.25 mm HP-5 column. Accurate mass data was obtained using GC-EI-MS from an Agilent 5977A GC/MSD using MassWorks 4.0 software from CERNO bioscience.[1] HPLC chromatograms were collected using an Agilent 1200 HPLC equipped with a 30 cm Chiralcel OD-H column. Optical rotations were recorded on an Anton Paar MCP 500 polarimeter with a 10.00 mm path length at 20 °C and 589 nm wavelength.

2 Details of flow reactor and equipment

2.1 Flow reactor setup:

A schematic of the reactor setup is shown in Figure S1 and a photo of the setup in the fumehood is shown in Figure S2. All pump feed tubes were either 1/16" O.D., 1.0 mm I.D. PFA or 1/8" O.D., 1/16" I.D. PFA. All tubes in the hydrogenation stage between the pump and the BPR were 1/16" O.D., 0.75 mm I.D. 316 Stainless steel (SS). All other tubing was 1/16" O.D., 0.5 mm I.D. PFA unless otherwise stated. PEEK fittings and tee mixers were used for all PFA and PEEK tubing as well as for stainless steel coil C1. 316 Stainless steel fittings and tees were used for all stainless steel tubes in the hydrogenation stage. PEEK fittings and parts were purchased from UpChurch Scientific. Stainless steel fittings and parts were purchased from VICI Valco or Swagelok.

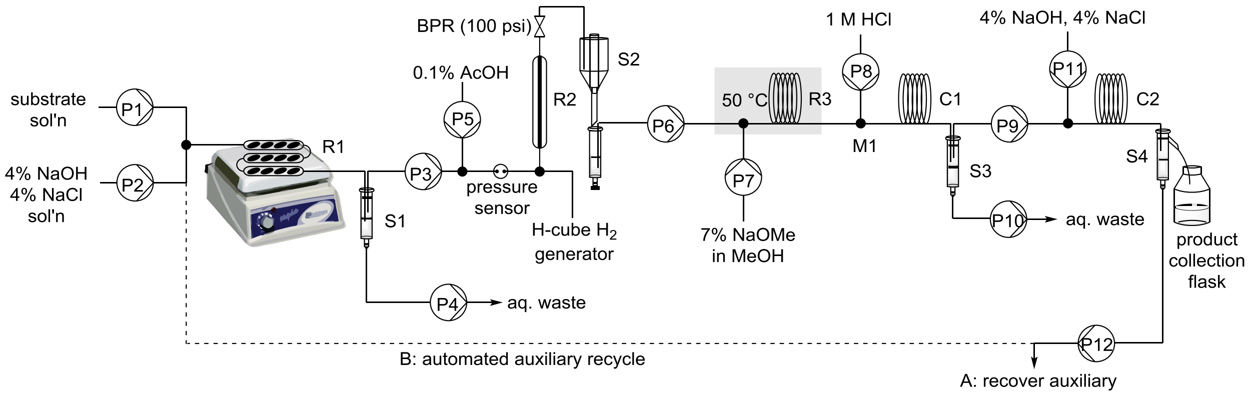

Figure S1. Schematic of complete flow reactor set up

P1 – SyrDos 2 continuous syringe pump equipped with 0.5 mL glass syringes. 200 µL/min.

A: 0.1 M acid chloride, 0.1 M sultam, 0.001 M aliquat 336 in toluene.

B: 0.1 M acid chloride, 0.035 M sutam, 0.001 M aliquat 336 in toluene.

P2 – New Era Pump Systems NE-1000 syringe pump equipped with either 60 or 25 mL luer-lock HSW norm-ject syringe. 150 µL/min.

A: 4% NaOH, 4% NaCl.

B: 4% NaOH, 4% NaCl with 0.87 M sultam for start-up only; replaced by the recovered auxiliary stream from P12 after reaching steady state.

P3 – ThalesNano micro HPLC pump, adjusted to maintain constant volume of organic phase in gravity phase separator.

P4 – ThalesNano micro HPLC pump, adjusted to maintain constant volume of aqueous phase in gravity phase separator.

P5 – ThalesNano micro HPLC pump. 50 µL/min.

P6 – Vapourtec V3 pump with red tubing, adjusted to match flow rate of organic phase exiting Biotage universal phase separator.

P7 – Chemyx fusion 200 syringe pump equipped with 25 mL stainless steel syringe (Harvard). 23.0 µL/min.

P8 – SyrDos 2 continuous syringe pump equipped with 2.5 mL glass syringes. 200 µL/min.

P9 – Global FIA milliGAT pump, adjusted to maintain constant volume of organic phase in gravity phase separator.

P10 – Vapourtec V3 pump with red tubing, adjusted to maintain constant aqueous volume in gravity phase separator.

P11 – SyrDos 2 continuous syringe pump equipped with 1.0 mL glass syringes. 150 µL/min.

P12 – Vapourtec V3 pump with red tubing, adjusted to maintain constant aqueous volume in gravity phase separator.

R1 – continuous stirred tubular reactors 3 × 0.5 mL. See section 2.2.

R2 – packed bed reactor, 1/4" O.D., 3 mm I.D. × 30 cm length packed with a mixture of 3 g 150–212 μm glass beads and 110 mg 10% Pd/C. See section 2.5.

R3 – 1.0 mL, 1/16" O.D., 0.75 mm ID PEEK tube.

C1 – 1.0 mL, 1/8" O.D., 2 mm I.D. 316 SS tube.

C2 – 200 μL, 1/16" O.D., 0.5 mm I.D. PFA tube.

M1 – tube-in-tee mixer, see section 2.6.

S1, S3, S4 – Gravity liquid-liquid separators – see section 2.3.

S2 – Modified Biotage Universal Phase Separator – see section 2.4.

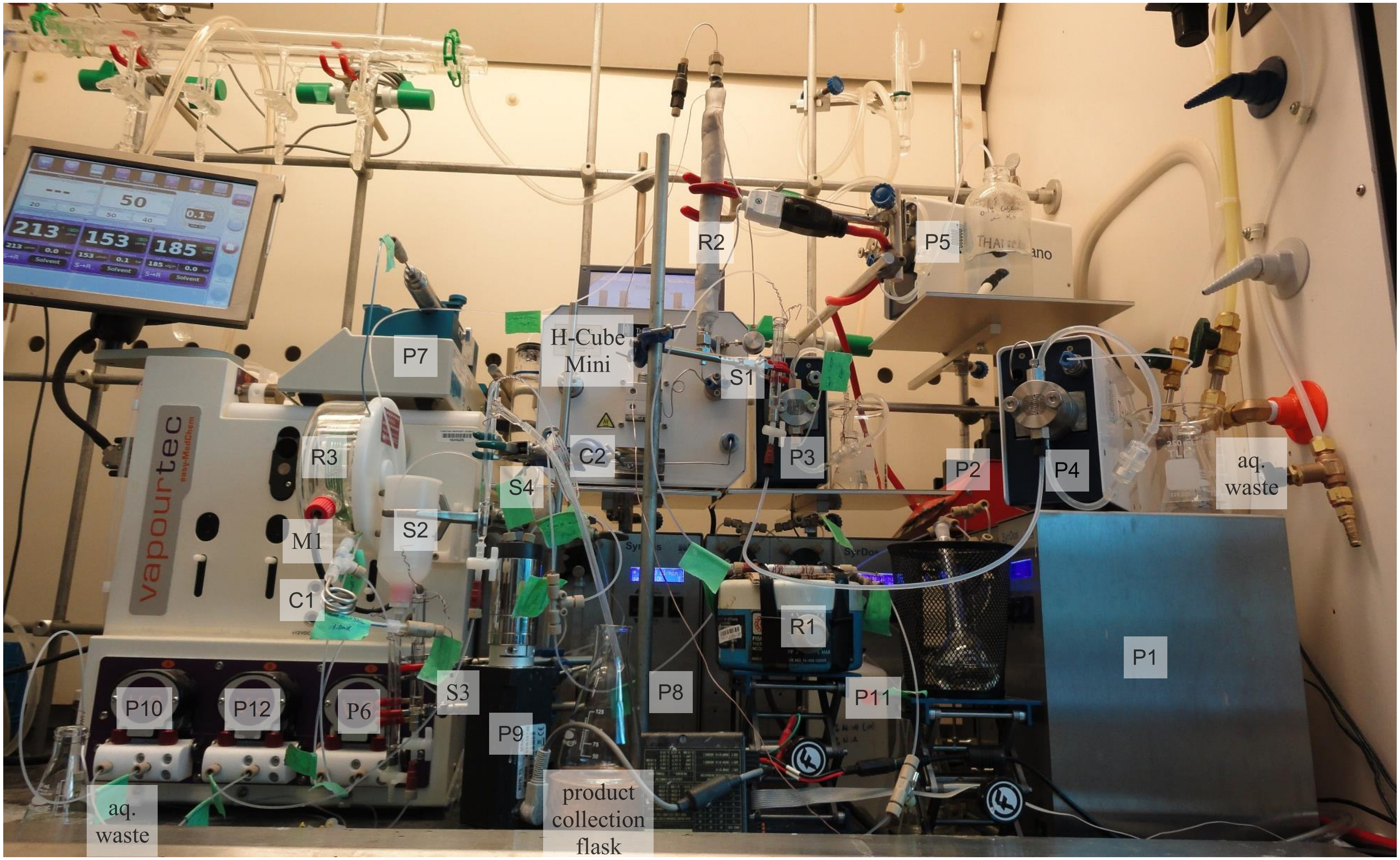

Figure S2. Photograph of complete flow reactor set up.

2.2 Active mixer reactor for acylation

R1 in Figure S1. The series of mixers was constructed by making active mixer units as reported by Ley and co-workers.[2] Three 1.00 mL plastic HSW syringes with the plungers removed were heated at the open end until soft and then a thread was cut using 1/4-28 PEEK male nuts. Four 3×10 mm PTFE coated stir bars were inserted in the mixer and PTFE tape was used to improve the seal between the PEEK fitting and the cut thread. The mixers were connected together with 10 cm of PFA tubing (1/16" O.D., 0.5 mm I.D., 20 μL). 100 cm PFA tubing (1/16" O.D., 0.5 mm I.D. 100 μL volume) was connected after last chamber to allow the emulsion to settle into plugs before entering the gravity liquid-liquid separator. The agitators were operated by a magnetic stir plate set at maximum (1400 rpm). A ~7.5 mm air gap between the stir plate surface and the mixers was maintained through use of cardboard spacers to prevent heat transfer from the stir plate surface that became warm to the touch with extended hours of operation.

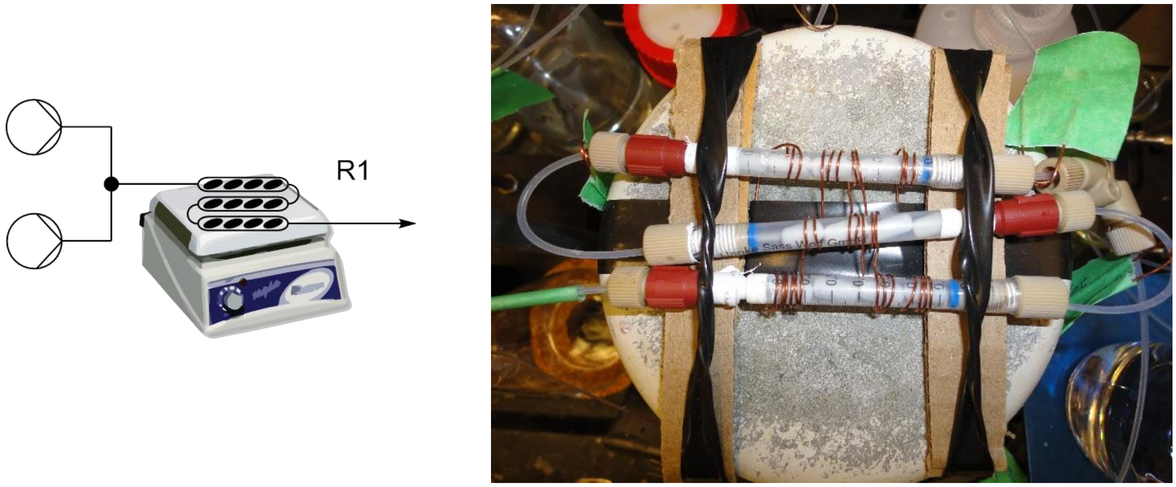

Figure S3. Schematic and photo of continuous stirred tubular reactor used for acylation

2.3 Gravity liquid-liquid separators

2.3.1 Gravity liquid-liquid separator with active withdrawal of both organic and aqueous phases (type 1)

S1 and S3 in Figure S1. A 2.5 mL glass Hamilton syringe was used for the body of the separator. The biphasic mixture was introduced at ~1 cm distance from bottom of chamber. The aqueous (lower) phase was withdrawn by pump from the luer joint at the bottom of syringe. The organic (upper) phase was withdrawn by pump through a tube ~1.5 cm from bottom of chamber. The phase boundary was maintained at ~0.75 cm from bottom of chamber by manually setting the pump withdrawal rates.

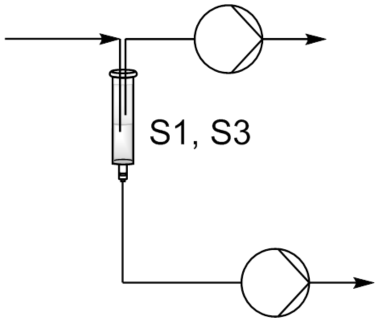

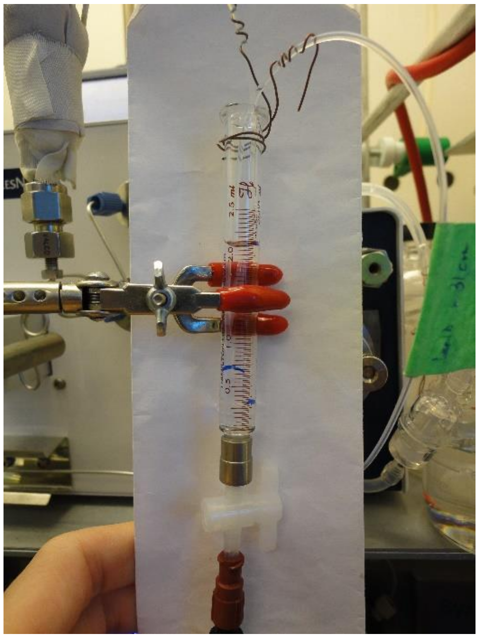

Figure S4. Schematic and photo of type 1 gravity liquid-liquid separator

2.3.2 Gravity liquid-liquid separators with active withdrawal of aqueous phase and passive withdrawal of organic phase (type 2)

S4 in Figure S1. An 8 mm O.D. 6 mm I.D. glass tube was cut to 12 cm length. Male luer joints were attached at the bottom and at 10 cm up the side by a glassblower. The biphasic mixture was introduced at ~3 cm distance from bottom of chamber. The aqueous (lower) phase was withdrawn by pump P12 from the luer joint at the bottom and the organic (upper) phase flowed out through the upper luer joint and was collected in a flask. The phase boundary was maintained at ~2.5 cm from the bottom by manually setting the pump withdrawal rate.

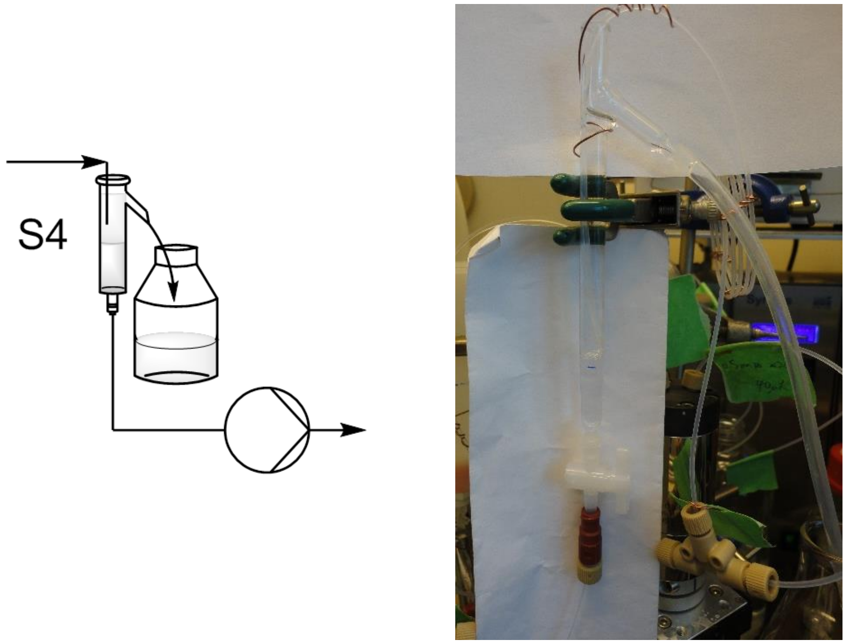

Figure S5. Schematic and photo of type 2 gravity liquid-liquid separator

2.4 Modifications to Biotage Universal Phase Separator

S2 in Figure S1. The membrane material of the Biotage Universal Phase Separator (product number 120-1930-V) was well suited for the separation after the hydrogenation, but the physical design of the unit required slight modification to adapt to the low flow rate of our scale. The membrane was cut to height of 4 cm to lower the volume of solvent retained in the membrane to ~1 mL, and the barrel of a 25 mL HSW syringe (with the end cut off to give an open cylinder) was placed around the membrane. The biphasic solution was introduced between the membrane and the syringe barrel. This kept the small volume of floating organic phase always in close contact with the membrane. The organic phase diffused through the membrane and was collected in a 2.5 mL glass Hamilton syringe body equipped with a closed two-way valve at the luer end, used as a solution reservoir for pump P6. The lower aqueous phase filled the entire reservoir both inside and outside the syringe barrel, allowing accumulation of the aqueous phase which was removed by Pastuer pipette approximately once per hour.

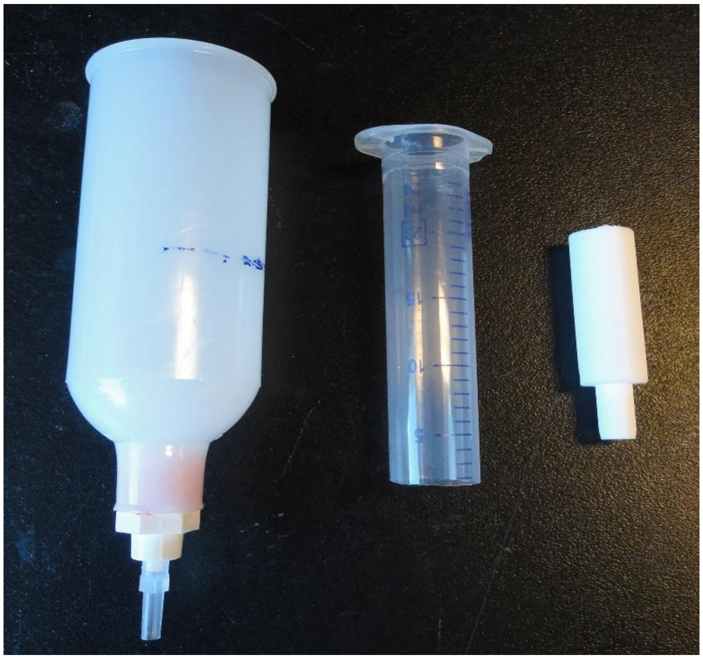

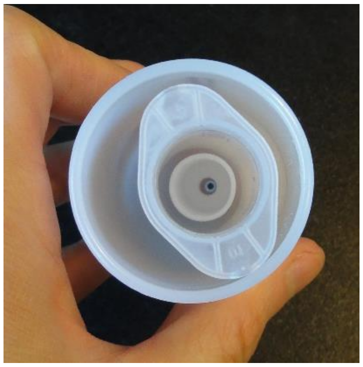

Figure S6. Photo of modified Biotage Universal Phase Separator components (left, outer reservoir, syringe body and shortened membrane) and assembled (right)

2.5 Packed bed reactor (PBR)

R2 in Figure S1. The packed bed reactor was fabricated from 30 cm of 1/4" O.D., 3.0 mm I.D. 316 stainless steel fitted with fritted HPLC column end fittings (Valco ECEF413.0F). A thermocouple was attached to the centre of the PBR and then a heating cable (McMaster Carr 3641K23) was wrapped the length of the column. The PBR was then wrapped in vinyl backed fibreglass insulation. The temperature of the PBR reactor was controlled by a J-Kem model 210 temperature controller. A 6.5 bar (100 psi) Upchurch back pressure regulator was placed after the PBR.

The packed bed reactor was interfaced with the commercially available H-Cube Mini from ThalesNano to provide *in situ* generated hydrogen. The decision to use an in-house fabricated PBR rather than purchasing catcarts was solely financially driven. Our PBR was re-packed after each experiment with a mixture of commercially available Pd/C (110 mg) and 150–212 μm glass beads (3.0 g, product G9018 from Sigma Aldrich) to limit pressure drop.[3]

Liquid volume of the packed bed was determined to be 1.0 mL by subtracting the mass once filled with water from the dry mass of the freshly backed PBR. Estimating equal occupancies of each of the 3 fluid phases (organic, aqueous and gaseous) gives a very rough estimate of residence time for the organic phase of 1.3 min. Measuring the time until breakthrough when introducing substrate through the clean packed bed was in rough agreement with this estimate.

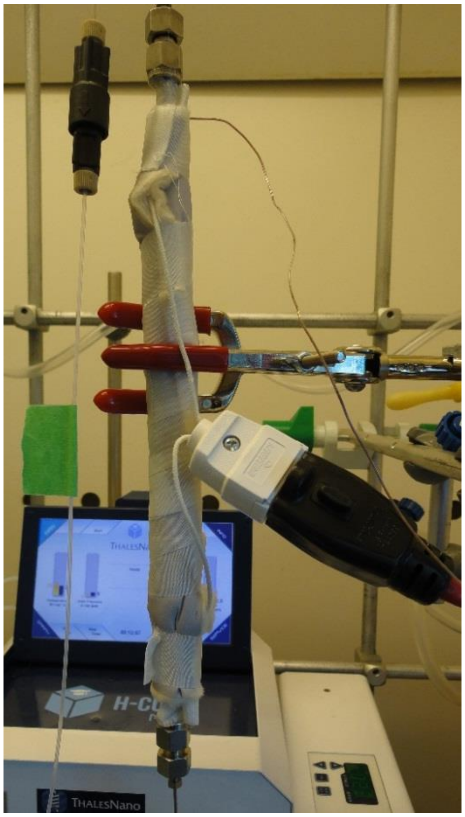

Figure S7. Photo of the in-house fabricated packed bed reactor

2.6 Tube-in-tee mixer

M1 in Figure S1. A tube-in-tee mixer[4] was used for the acid wash after the methanolysis. The NaOMe in methanol formed small plugs in the tolueune stream, causing inefficient quenching when mixing with HCl in a simple tee. The tube-in-tee was fabricated by taking an ordinary PFA tee mixer with 1.0 mm I.D. through-holes and enlarging the straight bore to 1/16" all the way through with a 1/16” drill bit, then further boring out one half of the straight bore to 2.0 mm I.D. as show in Figure S8.

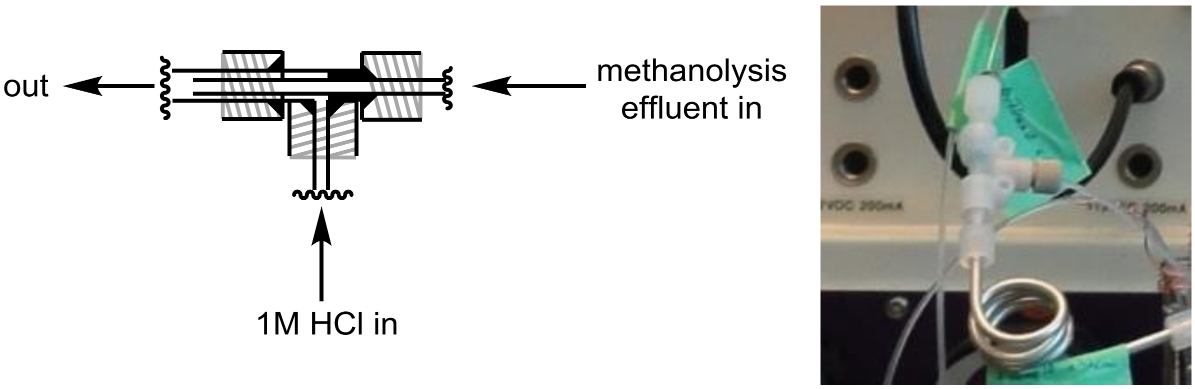

Figure S8. Schematic and photo of the in-house fabricated tube-in-tee mixer

3 Procedure for synthesis of substrates

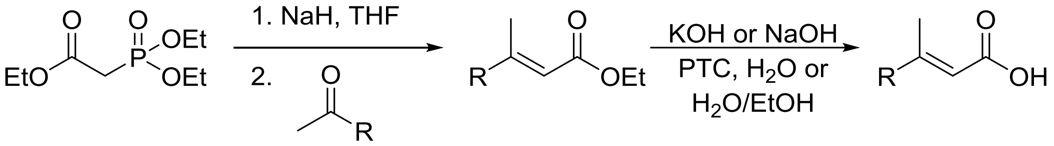

General procedure for synthesis of (*E*)-α-β-unsaturated acids: Based on the report by Seto and coworkers,[5] NaH (60% in mineral oil, 5 g, 125 mmol) was suspended in anhydrous THF (90 mL) under Ar and cooled to 0 ºC. Triethyl phosphonoacetate (30 g, 132 mmol) was added dropwise under Ar at 0 ºC. After the addition was complete the flask was allowed to warm to r.t. and stirred for 15 min until gas evolution ceased. Ketone (125 mmol) in THF (20 mL) was added dropwise. The resulting solution was stirred under Ar for 16 h. Water (100 mL) was added and the phases were separated. The aqueous phase was extracted with 2 × 50 mL Et_2O and all organic phases were combined and dried over Na_2SO_4 then concentrated to yield crude ethyl ester as a mixture of ~3:1 (*E*):(*Z*) isomers. The crude esters were then hydrolyzed and purified by varying conditions to yield pure *(E)*-α-β-unsaturated carboxylic acid.

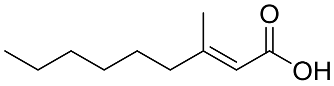

(*E*)-3-Methylnon-2-enoic acid: Prepared according to the general procedure and hydrolyzed by refluxing in 1 M NaOH (189 mL, 189 mmol) with 18-crown-6 (348 mg, 1.3 mmol) and 2,5-dimethyl-2,5-hexanediol (187 mg, 1.3 mmol) until the oil layer was gone (ca. 16 h). The mixture was cooled to room temperature, washed with 25 mL hexanes and the *p*H was adjusted to <1 with conc. HCl resulting in formation of a separate organic phase. The product was extracted with 3 × 25 mL hexanes, the combined organic extracts dried over Na_2SO_4 and the solvent evaporated to yield 18.97 g; 88% of ~3:1 (*E*):(*Z*)-3-methyl-2-nonenoic acid as a colourless oil. Repeated recrystallization from MeOH at -78 °C[5] yielded 5.42 g of >99% (*E*)-3-methylnon-2-enoic acid as a white powder that melted to a colourless oil at room temperature. ^{1}H NMR (400 MHz, $CDCl_3$): δ 12.17 (br s, 1H), 5.68 (sext, *J* = 1.2 Hz, 1H), 2.16 (t, *J* = 1.2 Hz, 3H), 2.15 (t, *J* = 7.5 Hz, 2H), 1.48 (pent, *J* = 7.3 Hz, 2H), 1.28 (m, 6H), 0.89 (T, *J* = 7.0 Hz, 3H). $^{13}C\{^1H\}$ NMR (100 MHz, $CDCl_3$): δ 172.7, 163.8, 115.2, 41.4, 31.7, 29.0, 27.5, 22.7, 19.2, 14.2. Characterization data is in agreement with the literature.[6]

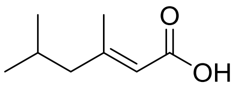

(*E*)-3,5-Dimethylhex-2-enoic acid: Prepared according to the general procedure and hydrolyzed by refluxing in 1 M NaOH (188 mL, 188 mmol), with 18-crown-6 (334 mg, 1.3 mmol) and 2,5-dimethyl-2,5-hexanediol (183 mg, 1.3 mmol) until the oil layer was gone (ca. 16 h). The mixture was cooled to room temperature, washed with 25 mL hexanes and the *p*H was adjusted to <1 with conc. HCl resulting in the formation of a separate organic phase. The product was extracted with 3 × 25 mL hexanes, the combined organic extracts dried over Na_2SO_4 and the solvent evaporated to yield 14.67 g; 81% of ~3:1 (*E*):(*Z*)-3,5-dimethylhex-2-enoic acid as a colourless oil. Repeated recrystallization from 4:1 MeOH:H_2O at -78 °C[5] yielded 2.21 g of >99% (*E*)-3-methyl-2-nonenoic acid as white needles. ^{1}H NMR (400 MHz, $CDCl_3$): δ 12.10 (br s, 1H), 5.67 (s, 1H), 2.15 (s, 3H), 2.03 (d, *J* = 7.2 Hz, 2H), 1.88 (sept, *J* = 6.8 Hz, 1H), 0.89 (d, *J* = 6.6 Hz, 6H). ^{13}C{^{1}H} NMR (100 MHz, $CDCl_3$): δ 172.5, 162.7, 116.4, 50.9, 26.5, 22.5, 19.1. Characterization data is in agreement with the literature.[7]

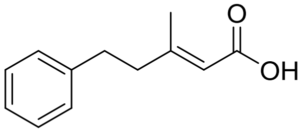

(*E*)-3-Methyl-5-phenylpent-2-enoic acid: Prepared according to the general procedure and hydrolyzed by addition of EtOH (100 mL), water (200 mL) and NaOH (65 g, 10 eq.)[8] and stirring the resulting biphasic mixture rapidly at room temperature for 5 h until a single phase was achieved. The solution was then washed with 50 mL of 1:1 hexanes:EtOAc followed by 25 mL of hexanes. The pH was adjusted to <1 with conc. HCl resulting in the formation of a separate organic phase that was collected. The aq. phase was extracted with 50 mL 1:1 hexanes:EtOAc then 25 mL hexanes and all organic phases were combined and dried over Na_2SO_4. The solvent was evaporated to yield an oil. EtOAc (100 mL) was added followed by cyclohexylamine (15.3 g, 154 mmol) with rapid stirring, resulting in a thick white slurry of 3-methyl-5-phenyl-2-pentenoic acid·cyclohexylamine salt that was recrystallized twice from EtOAc[9] to remove the *z* isomer. The resulting white solid was then added to 3 M HCl (100 mL) and the free acid was extracted with 2×25 mL DCM. The combined extracts were dried over Na_2SO_4 and the solvent evaporated yielding a colourless oil. Boiling hexanes (80 mL) were added resulting in a fine suspension that was hot filtered and cooled to –20 °C yielding colourless crystals of the desired product in 97% purity. Recrystallizing a second time from hexanes yielded white needles of pure (*E*)-3-methyl-5-phenylpent-2-enoic acid. Yield 8.75 g; 37%. ^{1}H NMR (400 MHz, $CDCl_3$): δ 11.98 (s, br, 1H), 7.31 (m, 2H), 7.20 (m, 3H), 5.72 (q, *J* = 1.2 Hz, 1H), 2.81 (dd, *J* = 9.4, 6.7 Hz, 2H), 2.49 (dd, *J* = 9.3, 6.2 Hz, 2H), 2.23 (d, *J* = 1.2 Hz, 3H).

$^{13}C\{^1H\}$ NMR (100 MHz, $CDCl_3$): δ 172.3, 162.3, 141.0, 128.6, 128.4, 126.3, 115.7, 43.1, 34.0, 19.4. Characterization data is in agreement with the literature.[6]

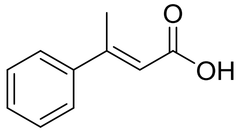

(*E*)-3-Phenylbut-2-enoic acid: Prepared as reported by Yamada and coworkers.[10] Cs_2CO_3 (26.07 g, 80 mmol), DBU (3.00 g, 20 mmol) and triethylphosphonoacetate (22.41 g, 100 mmol) were combined. Acetophenone (12.00 g, 100 mmol) was added and the slurry stirred at 40 °C for 3 days under Ar. The reaction was then quenched with H_2O (100 mL) and extracted with 3×25 mL hexanes and the combined organic extracts were dried over Na_2SO_4. The solvent was removed *in vacuo* yielding 19.34 g of a mixture of the desired product and acetophenone. To the crude mixture was added KOH (8.4 g, 150 mmol) and MeOH (100 mL) and the solution was refluxed overnight. The MeOH was then evaporated and H_2O (100 mL) and EtOAc (50 mL) were added. The mixture was agitated then allowed to settle. The organic phase was discarded and the aqueous phase was washed with 25 mL hexanes. The aqueous phase was then acidified to *p*H <1 with conc. HCl resulting in formation of a separate organic phase. The product was extracted with 50 mL 1:1 EtOAc:hexanes then 2 × 25 mL hexanes. The combined extracts were dried over Na_2SO_4 and evaporated *in vacuo*. The crude yellow powder was recrystallized from hexanes yielding white needles of pure (*E*)-3-phenylbut-2-enoic acid. Yield 5.41 g; 33%. ^1H NMR (400 MHz, $CDCl_3$): δ 12.02 (br s, 1H), 7.51 (m, 2H), 7.40 (m, 3H), 6.19 (q, J = 1.3 Hz, 1H), 2.62 (d, J = 1.3 Hz, 3H). $^{13}C\{^1H\}$ NMR (100 MHz, $CDCl_3$): δ 172.6, 158.7, 142.2, 129.5, 128.7, 126.6, 116.6, 18.5. Characterization data is in agreement with the literature.[7]

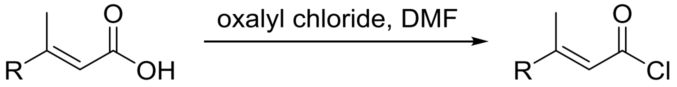

General procedure for synthesis of (*E*)- α-β-unsaturated acid chlorides: Et_2O (2.5 mL), DMF (7 μL, 0.1 mmol) and oxalyl chloride (1.7 mL, 20 mmol) were combined under Ar and the flask placed in a room temperature water bath. *(E)*-α-β-unsaturated acid (10 mmol), dissolved in Et_2O (7.5 mL), was added in small portions over the course of 30 min to the stirred oxalyl chloride solution. The mixture was stirred one additional hour after the addition was complete, then the Et_2O and excess oxalyl chloride were removed *in vacuo*.

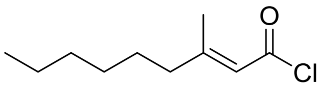

(*E*)-3-Methylnon-2-enoic acid chloride: Prepared according to the general procedure. Purified by Kugelrohr distillation at 95 °C under high vacuum. Yield 1.81 g; 96%; 99.2:0.8 (*E*):(*Z*) (relative GC-FID area count). The colourless oil was stored at –20 °C in a N_2 atmosphere glovebox. ^{1}H NMR (400 MHz, $CDCl_3$): δ 6.02 (sext, *J* = 1.2 Hz, 1H), 2.19 (td, *J* = 7.6, 1.0 Hz, 2H), 2.13 (d, *J* = 1.2 Hz, 3H), 1.49 (pent, *J* = 7.6 Hz, 2H), 1.29 (m, 6H), 0.88 (t, *J* = 7.0 Hz, 3H). ^{13}C{^{1}H} NMR (100 MHz, $CDCl_3$): δ 168.2, 163.9, 122.1, 41.1, 31.7, 29.0, 27.4, 22.6, 20.5, 14.2.

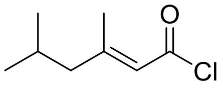

(*E*)-3,5-Dimethylhex-2-enoic acid chloride: Prepared according to the general procedure. Purified by Kugelrohr distillation at 56 °C under high vacuum. Yield 1.51 g; 92%; 99.5:0.5 (*E*):(*Z*) (relative GC-FID area count). The colourless oil was stored at –20 °C in a N_2 atmosphere glovebox. ^{1}H NMR (400 MHz, $CDCl_3$): δ 6.01 (sext, *J* = 1.1 Hz, 1H), 2.12 (d, *J* = 1.2 Hz, 3H), 2.06 (d, *J* = 7.1 Hz, 2H), 1.90 (nonet, *J* = 6.6 Hz, 1H), 0.91 (d, *J* = 6.6 Hz, 6H). ^{13}C{^{1}H} NMR (100 MHz, $CDCl_3$): δ 167.1, 163.8, 123.2, 50.4, 26.8, 22.5, 20.4. Characterization data is in agreement with the literature.[11]

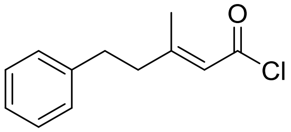

(*E*)-3-Methyl-5-phenylpent-2-enoic acid chloride: Prepared according to the general procedure. The crude acid chloride was stored at –20 °C in a N_2 atmosphere glovebox overnight and then used without further purification the following day. Yield 1.84 g; 88%; >99.5:0.5 (*E*):(*Z*) (relative GC-FID area count). ^{1}H NMR (400 MHz, $CDCl_3$): δ 7.31 (t, *J* = 7.5 Hz, 2H), 7.23 (t, *J* = 7.3 Hz, 1H), 7.17 (d, *J* = 6.8 Hz, 2H), 6.03 (q, *J* = 1.2 Hz, 1H), 2.82 (dd, *J* = 9.1, 6.7 Hz, 2H), 2.52 (dd, *J* = 9.4, 6.4 Hz, 2H), 2.18, (d, *J* = 1.2 Hz, 3H). ^{13}C{^{1}H} NMR (100 MHz, $CDCl_3$): δ 166.3, 164.0, 140.2, 128.8, 128.4, 126.6, 122.7, 42.7, 33.8, 20.6.

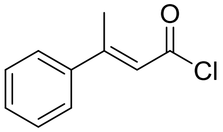

(*E*)-3-Phenylbut-2-enoic acid chloride: Prepared according to the general procedure except 22 mL of Et_2O was necessary to dissolve the acid and addition was over 60 min. Purified by Kugelrohr distillation

at 103 °C under high vacuum. Yield 1.66 g; 98 %; 99:1 (*E*):(*Z*) (relative GC-FID area count). The bright yellow oil was stored at –20 °C in a N_2 atmosphere glovebox. ^{1}H NMR (400 MHz, $CDCl_3$): δ 7.52 (m, 2H), 7.44 (m, 3H), 6.48 (q, *J* = 1.2 Hz, 1H), 2.56 (d, *J* = 1.2 Hz, 3H). $^{13}C\{^{1}H\}$ NMR (100 MHz, $CDCl_3$): 164.2, 161.4, 140.3, 130.7, 128.9, 126.8, 122.7, 19.4. Characterization data is in agreement with the literature.[11]

4 Experimental details from optimization of reaction steps for flow

4.1 Acylation optimization from Table 1

DBU, Et_3N, DBU/DMAP and Et_3N/DMAP were screened as organic bases in small scale batch experiments with 3,3-dimethylacrylic acid chloride and camphorsultam. Low to moderate yields were obtained and precipitate formation was observed, suggesting conversion to flow may be problematic.

PTC in batch: (*E*)-3-methylnon-2-enoic acid chloride (0.05 mmol), aliquat 336 (1 mol%)[12], hexadecane (0.005 mmol; istd) and camphorsultam (0.05 mmol) were dissolved in toluene (0.5 mL). 4% NaOH (0.5 mL) was added and the biphasic mixture was stirred at 1400 rpm. Yields were ~quantitative as monitored by GC analysis.

PTC with passive mixing in flow: 0.1 M (*E*)-3-methylnon-2-enoic acid chloride with 0.001M aliquat 336 (1 mol%), 0.01 M hexadecane (istd) and 0.1 M camphorsultam in toluene were pumped at 200µL min and mixed in a PEEK T-mixer with 4% (w/w) NaOH pumped at either 150 or 200 µL min and then passed through a 4.00 mL 1/16" O.D., 1.0 mm I.D. PFA coil. Yield varied with time due to poor mixing, ranging from poor to moderate as monitored by off-line GC-FID analysis.

PTC with active mixing in flow: 0.1 M (*E*)-3-methylnon-2-enoic acid chloride with 0.001M aliquat 336 (1 mol%), 0.01 M hexadecane (istd) and 0.1 M camphorsultam in toluene were pumped at 200µL min and mixed in a PEEK T-mixer with 4% NaOH at 150 or 200 µL/min then passed through the active mixer reactor (Figure S3). Yields were ~quantitative as monitored by off-line GC analysis. 4% NaCl could be added to the NaOH feed to improve downstream separations without influencing the yield.

4.2 Hydrogenation in PBR from Equation 1

Start-up as per the description in section 5.1 general procedures for process start-up but with only the acylation and hydrogenation stages telescoped together. 97:3 (*E*):(*Z*)-3-methylnon-2-enoic acid chloride was used as the substrate. The effluent was collected for 1 h 50 min then evaporated and the residue chromatographed (silica gel, 3:1 hexanes:Et_2O) to yield 790 mg; 97% hydrogenated product, d.r.: 90:10.

4.3 Methanolysis optimization from Table 2

Various phase transfer catalysts were screened in batch. The combination of 18-crown-6 and 2,5-dimethyl-2,5-hexanediol[13] with 55% (w/w) KOH $_{(aq)}$ and 0.1 M substrate in toluene was effective but when transitioning into flow precipitate formation and slow reaction rates were problematic.

Delivering instead KOH in MeOH (30% w/w, 18 µL/min, 5 eq.) and mixing with 0.1 M substrate in toluene (200 µL/min) in a PEEK T-mixer then passing through a residence coil of either 1.0 or 2.0 mL volume of either 1/16" O.D., 1.0 mm I.D. PFA or 1/16" O.D., 0.75 mm I.D. PEEK at various temperatures led to identification of conditions (90 °C, 9 min in 2.0 mL, 0.75 mm I.D. PEEK) that achieved complete consumption of starting material but precipitate formed in the tubing resulting in variable yields and would likely lead to eventual clogging.

Delivering NaOMe instead of KOH in MeOH greatly improve the reaction rate and homogeneity. Screening the NaOMe concentration (from 25% to 7% w/w), equivalents of NaOMe (from 1.25 to 2.0 eq.), and residence coil (0.5, 1.0 or 2.0 mL volume of 1/16" O.D., 0.75 mm I.D. PEEK) led to identification of conditions (50 °C, 4.5 min in 1.0 mL, 1/16" O.D., 0.75 mm I.D. coil) that gave consistent high yields for both the methyl ester and recovered auxiliary without precipitation and could be telescoped into the full process without issues. Full details of telescoped reactions in section 5.

5 Procedure for telescoped flow synthesis of chiral materials

5.1 General procedure for process start-up:

The system was assembled as described in Section 2. The packed bed reactor was heated to 45 °C and conditioned by wetting with toluene (400 µL/min) then eluting with the biphasic mixture of toluene (400 µL/min) and 0.1% acetic acid (100 µL/min) until the pH of the aqueous phase exiting the PBR was <2. The flow rates were then decreased to the process flow rates (200 µL/min toluene, 50 µL/min 0.1% AcOH) and the H-cube H_2 production was turned on. The packed bed was then conditioned with these parameters for 30 min while the acylation phase was reaching steady state, before the toluene feed was changed to the process feed. The resulting pressure drop over the conditioned packed bed reactor was typically ~30 bar.

The process was started consecutively in phases beginning with the acylation reaction and allowing each phase to reach steady state (discarding the first ~three residence volumes) before diverting effluent to in-line phase separators S1–S4. After filling the phase separator, the next phase was commenced. Times to steady state: acylation: 14 min; hydrogenation: 15 min; methanolysis and acid wash: 25 min; auxiliary

extraction: 10 min, plus 10 min for each in-line phase separator ≈ 2 h total for start-up. Each gravity liquid/liquid phase separator was maintained with ~1 mL of organic phase and ~0.25 mL of aqueous phase. The flow rate of pumps internal to the flow path (i.e., withdrawing from phase separators) were adjusted to maintain a constant volume in the separator (i.e., flow in = flow out for each liquid phase). Diastereoselectivity of the hydrogenation reaction was monitored off-line by withdrawing a ~25 μL aliquot from the post-hydrogenation organic phase and submitting to GC analysis every 30 min (see Section 9).

5.2 Telescoped process with auxiliary recovery

The acid chloride, auxiliary and PTC were all combined in the organic solution and 4% (w/w) NaOH, 4% (w/w) NaCl was used as the aqueous solution for the PTC acylation. No background sultam amide formation in the stock solution was observed over the timeframe of the experiment (~6 h).

The acid chloride solutions were prepared by combining *(E)*-α-β-unsaturated acid chloride (6.0 mmol), toluene (~5 mL), aliquat 336 (24 mg, 60 μmol) and camphorsultam (1.29 g, 6.0 mmol), then making up the solution to 60.00 mL with toluene.

After steady state was reached for the entire process, collection of the product and recovered auxiliary streams commenced. Effluents were collected for 3 h, then all pumps were stopped. The product phase effluent was evaporated and the methyl ester was purified by chromatography (20×150 mm silica gel stationary phase, pentane→5% Et_2O in pentane mobile phase).

The recovered auxiliary phase effluent was acidified to *p*H<1 with conc. HCl and then extracted with 2×15 mL DCM. The combined organic extracts were dried over Na_2SO_4, and evaporated to yield the crude auxiliary. Recrystallization from hexanes gave white needles of pure camphorsultam.

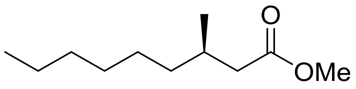

(*R*)-3-Methylnonanoic acid methyl ester: Yield: 447 mg; 67%. ^{1}H NMR (400 MHz, $CDCl_3$): δ 3.64 (s, 3H), 2.28 (dd, *J* = 14.7, 6.0 Hz, 1H), 2.08 (dd, *J* = 14.7, 8.2 Hz, 1H), 1.92 (m, 1H), 1.24 (m, 10H), 0.90 (d, *J* = 6.7 Hz, 3H), 0.86 (t, *J* = 6.6 Hz, 3H). ^{13}C{^{1}H} NMR (100 MHz, $CDCl_3$): δ 173.9, 51.4, 41.8, 36.8, 32.0, 30.5, 29.5, 27.0, 22.7, 19.8, 14.2. $[\alpha]_D^{20}$ = +3.8 ° (c = 1.03, CH_2Cl_2) [lit.[14] $[\alpha]_D^{25}$ = +5.9 ° (c = 4.70, $CHCl_3$)]. Characterization data is in agreement with the literature.[14] Crude sultam recovery: 79%; recrystallized: 410 mg, 53%. Enantiomeric ratio was taken from diastereomeric ratio data: 95 (*R*): 5 (*S*).

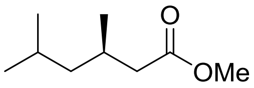

(R)-3,5-Dimethylhexanoic acid methyl ester: Yield: 410 mg; 72%. ^{1}H NMR (400 MHz, $CDCl_3$): δ 3.66 (s, 3H), 2.27 (dd, J = 14.2, 5.3 Hz, 1H), 2.08 (dd, J = 14.2, 8.3, 1H), 2.01 (octet, J = 6.2 Hz, 1H), 1.61 (nonet, J = 6.6 Hz, 1H), 1.08 (m, 2H), 0.90 (d, J = 6.4 Hz, 3H), 0.86 (t, J = 6.5 Hz, 6H). $^{13}C\{^{1}H\}$ NMR (100 MHz, $CDCl_3$): δ 173.9, 51.6, 46.4, 42.1, 28.2, 25.3, 23.3, 22.2, 19.9. $[\alpha]_D^{20}$ = +4.5 ° (c = 1.05, CH_2Cl_2). Accurate mass m/z = 158.1268, spectral accuracy 98%, calc: 158.1301. Crude sultam recovery: 71%; recrystallized: 420 mg, 54%. Enantiomeric ratio was taken from diastereomeric ratio data: 98 (R): 2 (S).

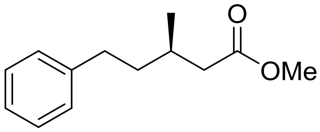

(R)-3-Methyl-5-phenlybutanoic acid methyl ester: Yield: 528 mg; 71%. ^{1}H NMR (400 MHz, $CDCl_3$): δ 7.28 (m, 2H), 7.18 (m, 3H), 3.67 (s, 3H), 2.62 (m, 2H), 2.36 (dd, J = 14.7, 6.0 Hz, 1H), 2.18 (dd, J = 14.8, 8.0 Hz, 1H), 2.02 (oct, J = 6.5 Hz, 1H), 1.67 (m, 1H), 1.53 (m, 1H), 1.01 (d, 6.6 Hz, 3H). $^{13}C\{^{1}H\}$ NMR (100 MHz, $CDCl_3$): δ 173.6, 142.4, 128.45, 128.42, 125.8, 51.5, 41.6, 38.6, 33.4, 30.2, 19.8. $[\alpha]_D^{20}$ = +14.7 ° (c = 1.05, CH_2Cl_2) [lit.[15] $[\alpha]_D^{25}$ = +18.5 ° (c = 0.88, CH_2Cl_2)]. Characterization data is in agreement with literature.[16] Enantiomeric ratio was confirmed by chiral HPLC using a 30 cm Chiralcel OD-H column, 0.5 mL/min 99.7% hexanes/0.3% ^{i}PrOH, retention times 49.3 min (minor) and 60.3 min (major):[16] 93.4 (R): 6.6 (S); in agreement with diastereomeric ratio data (93:7). Crude sultam recovery: 73%; recrystallized: 435 mg, 56%.

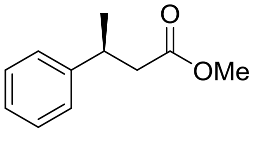

(S)-3-Phenylbutanoic acid methyl ester: Yield: 431 mg; 67%. ^{1}H NMR (400 MHz, $CDCl_3$): δ 7.31 (m, 2H), 7.21 (m, 3H), 3.63 (s, 3H), 3.29 (hextet, J = 7.3 Hz, 1H), 2.60 (ABX pattern, J = 15.2, 8.2, 6.9 Hz, 2H), 1.3 (d, J = 7.6 Hz, 3H). $^{13}C\{^{1}H\}$ NMR (100 MHz, $CDCl_3$): δ 172.9, 145.8, 128.6, 126.8, 126.5, 51.6, 42.8, 36.5, 21.9. $[\alpha]_D^{20}$ = +23.5 ° (c = 0.99, CH_2Cl_2) [lit.[17] $[\alpha]_D^{20}$ = +7.3 ° (c = 1.36, $CHCl_3$, ee = 44%)]. Characterization data is in agreement with literature.[17] Crude sultam recovery: 72%; recrystallized: 358 mg, 46%. Enantiomeric ratio was taken from diastereomeric ratio data: 92 (S): 8 (R).

5.3 Telescoped process with auxiliary recycling

For auxiliary recycle experiments the acid chloride, 35% auxiliary make up and PTC were combined in the organic solution and the other 65% of auxiliary was dissolved in a solution of 4% NaOH, 4% NaCl used for the acylation during start-up.

The acid chloride solutions were prepared by combining *(E)*-α-β-unsaturated acid chloride (7.5 mmol), toluene (~5 mL), aliquat 336 (30 mg, 75 μmol) and camphorsultam (0.56 g, 2.6 mmol), then making up the solution to 75.00 mL with toluene.

The auxiliary in 4% NaOH, 4% NaCl solution used for start-up was prepared by taking camphorsultam (0.46 g, 2.2 mmol) and making up to 25.00 mL with 4% NaOH, 4% NaCl.

After steady state was reached for the entire process as in the single pass experiments, collection of the product stream was commenced and the recovered auxiliary stream was connected to the acylation phase, completing the recycle loop. Product containing effluent was collected for 4.5 h, then all pumps were stopped. The effluent was evaporated and the methyl ester purified by chromatography (20×150 mm silica gel stationary phase, pentane→5% Et_2O in pentane mobile phase).

(*R*)-3-Methylnonanoic acid methyl ester: Yield: 548 mg; 54%, contaminated with ~33 mg (6% by mass) rac-3-methylnonanal impurity. The aldehyde impurity was removed by treating the product with Brady's reagent (2,4-dinitrophenylhydrazine, 100 mg, 0.5 mmol) in MeOH (10 mL) with 1 drop H_2SO_4 at 40 °C for 20 min and then separation of the formed hydrazone from the methyl ester by chromatography (20 × 150 mm silica gel column, pentane→5% Et_2O in pentane eluent) giving the methyl ester as a pale yellow oil. Kugelrohr distillation at 80 °C, high vacuum yielded the pure methyl ester product as a colourless oil, 444 mg; 44%. Enantiomeric ratio taken from diastereomeric ratio data: 95 (*R*): 5 (*S*).

(*R*)-3,5-Dimethylhexanoic acid methyl ester: Yield: 411 mg, 48%. Enantiomeric ratio taken from diastereomeric ratio data: 98 (*R*): 2 (*S*).

(*R*)-3-Methyl-5-phenlybutanoic acid methyl ester: Yield: 640 mg, 57%. Enantiomeric ratio taken from diastereomeric ratio data: 95 (*R*): 5 (*S*).

(*S*)-3-Phenylbutanoic acid methyl ester: Yield: 658 mg, 68%. Enantiomeric ratio taken from diastereomeric ratio data: 90 (*S*): 10 (*R*).

6 Spectra of substrates and products

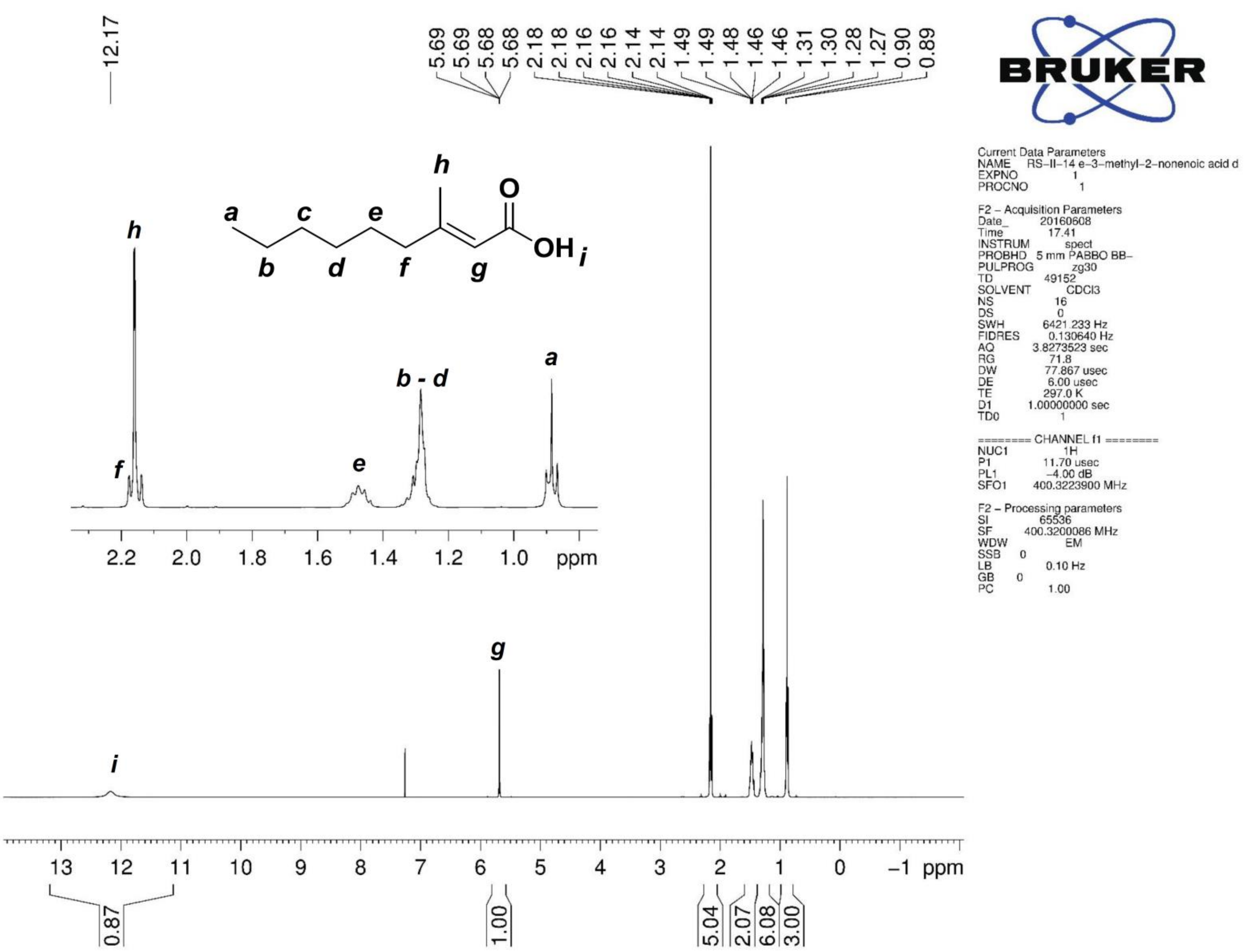

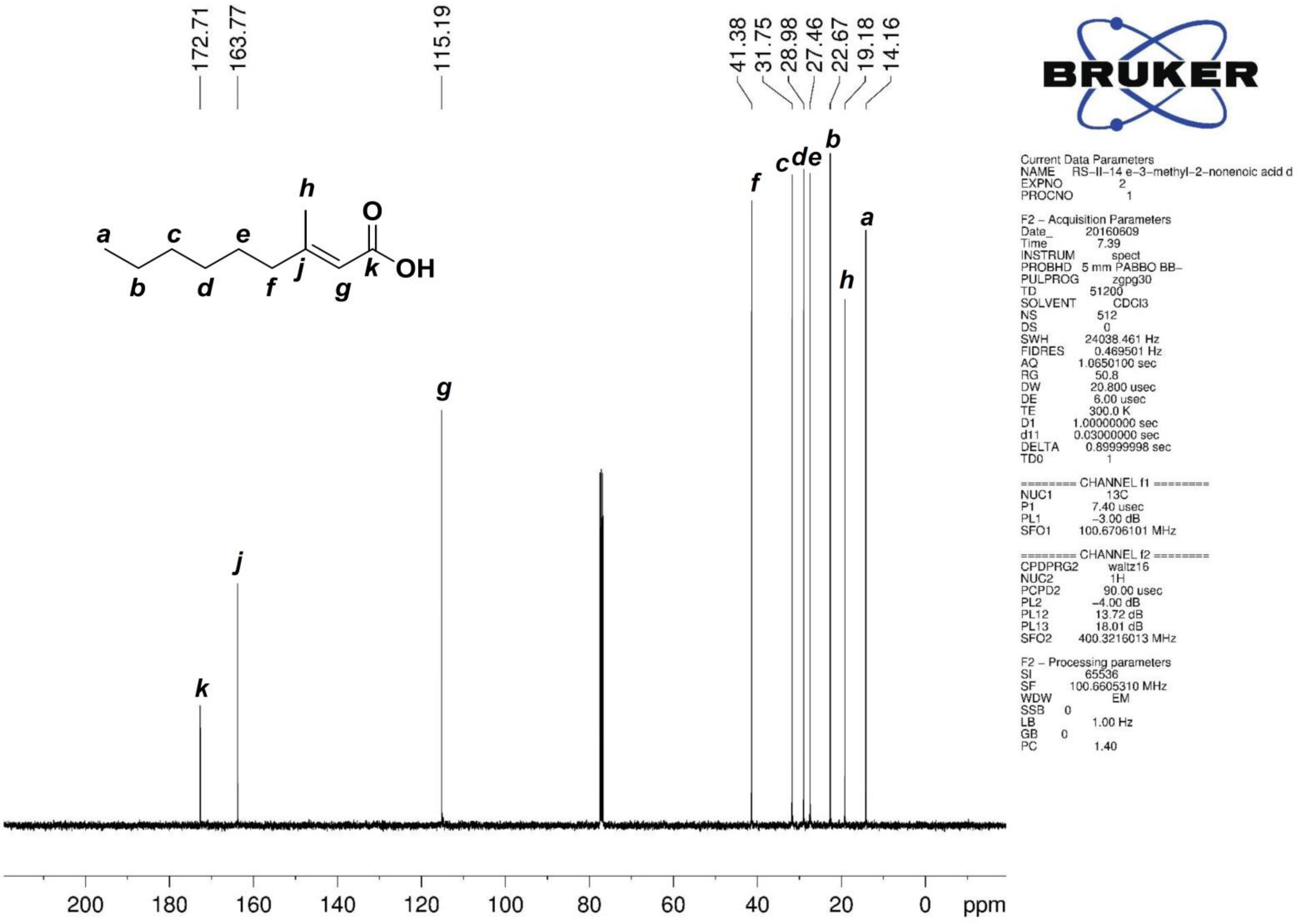

172.71
163.77
115.19
41.38
31.75
28.98
27.46
22.67
19.18
14.16
a b c d e f g h j k
O
OH
200 180 160 140 120 100 80 60 40 20 0 ppm
BRUKER
Current Data Parameters
NAME RS–II–14 e–3–methyl–2–nonenoic acid d
EXPNO 2
PROCNO 1
F2 – Acquisition Parameters
Date_ 20160609
Time 7.39
INSTRUM spect
PROBHD 5 mm PABBO BB–
PULPROG zgpg30
TD 51200
SOLVENT CDCl3
NS 512
DS 0
SWH 24038.461 Hz
FIDRES 0.469501 Hz
AQ 1.0650100 sec
RG 50.8
DW 20.800 usec
DE 6.00 usec
TE 300.0 K
D1 1.00000000 sec
d11 0.03000000 sec
DELTA 0.89999998 sec
TD0 1
======== CHANNEL f1 ========
NUC1 13C
P1 7.40 usec
PL1 –3.00 dB
SFO1 100.6706101 MHz
======== CHANNEL f2 ========
CPDPRG2 waltz16
NUC2 1H
PCPD2 90.00 usec
PL2 –4.00 dB
PL12 13.72 dB
PL13 18.01 dB
SFO2 400.3216013 MHz
F2 – Processing parameters
SI 65536
SF 100.6605310 MHz
WDW EM
SSB 0
LB 1.00 Hz
GB 0
PC 1.40

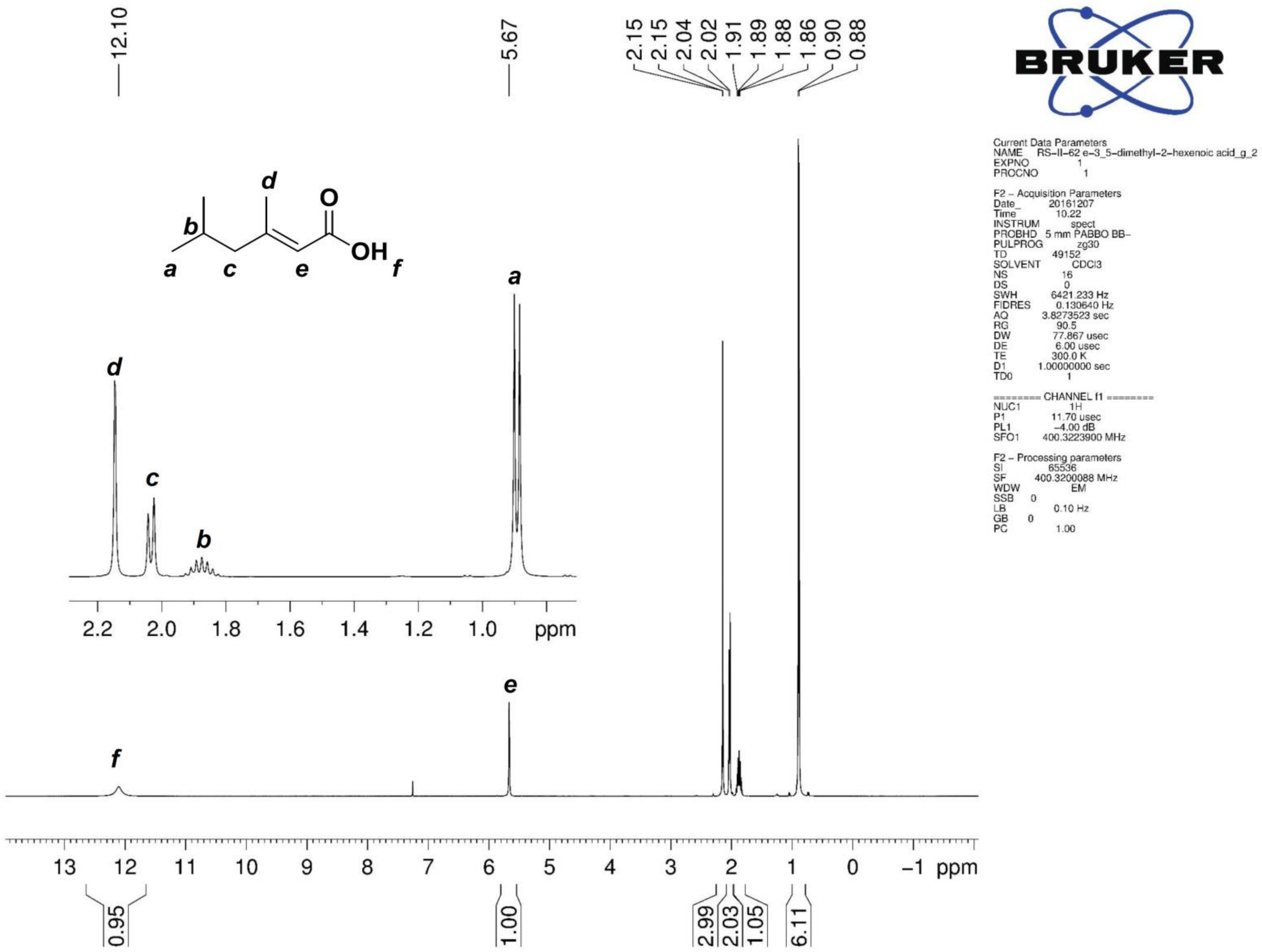
12.10
5.67
2.15
2.15
2.04
2.02
1.91
1.89
1.88
1.86
0.90
0.88
d
O
b
OH
a
c
e
f
a
d
c
b
2.2
2.0
1.8
1.6
1.4
1.2
1.0
ppm
e
f
13
12
11
10
9
8
7
6
5
4
3
2
1
0
−1
ppm
0.95
1.00
2.99
2.03
1.05
6.11
Current Data Parameters
NAME RS−II−62 e−3_5−dimethyl−2−hexenoic acid_g_2
EXPNO 1
PROCNO 1
F2 − Acquisition Parameters
Date_ 20161207
Time 10.22
INSTRUM spect
PROBHD 5 mm PABBO BB−
PULPROG zg30
TD 49152
SOLVENT CDCl3
NS 16
DS 0
SWH 6421.233 Hz
FIDRES 0.130640 Hz
AQ 3.8273523 sec
RG 90.5
DW 77.867 usec
DE 6.00 usec
TE 300.0 K
D1 1.00000000 sec
TD0 1
======== CHANNEL f1 ========
NUC1 1H
P1 11.70 usec
PL1 −4.00 dB
SFO1 400.3223900 MHz
F2 − Processing parameters
SI 65536
SF 400.3200088 MHz
WDW EM
SSB 0
LB 0.10 Hz
GB 0
PC 1.00

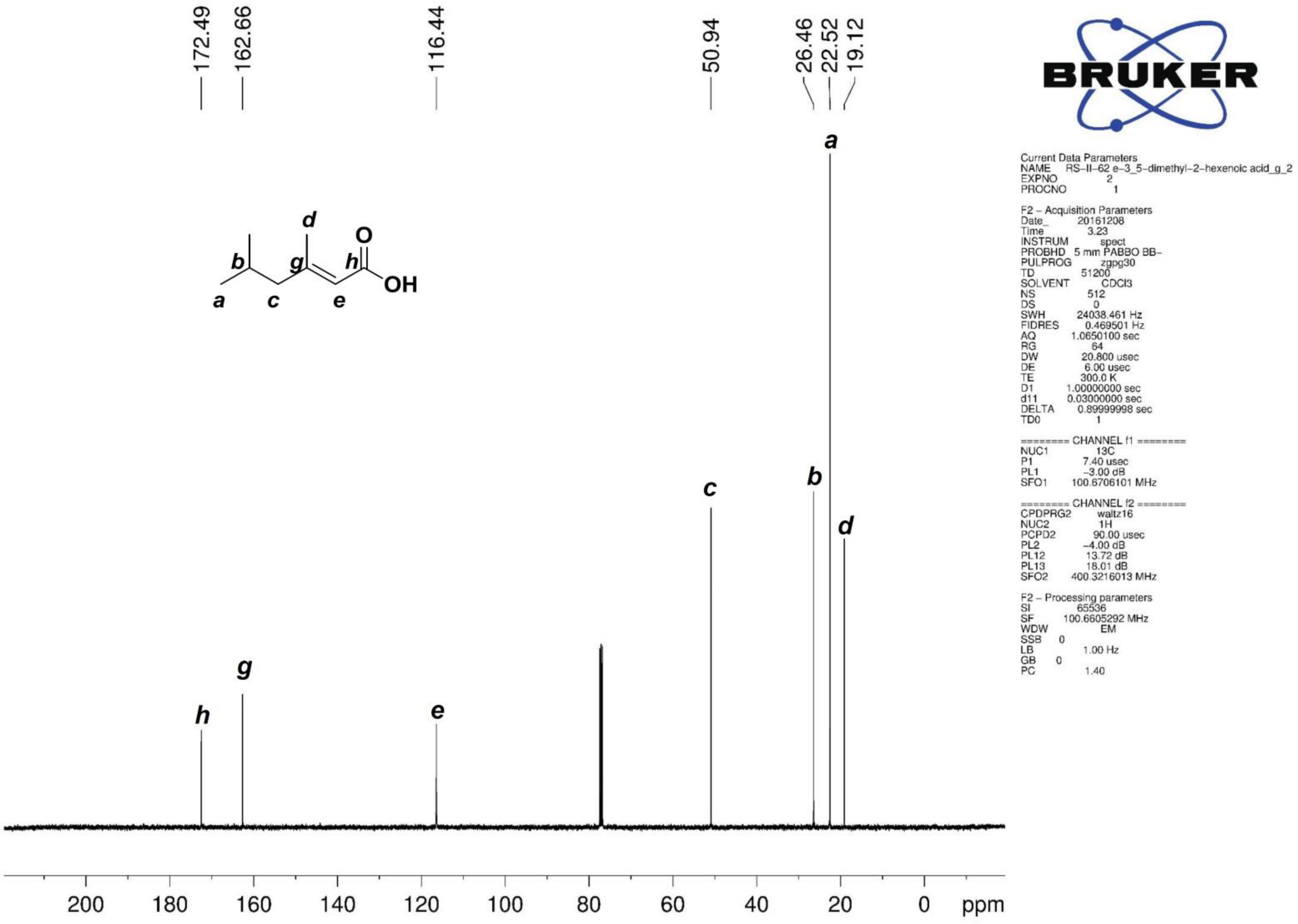
172.49
162.66
116.44
50.94
26.46
22.52
19.12
a
b
c
d
e
g
h
O
OH
BRUKER
Current Data Parameters
NAME RS–II–62 e–3_5–dimethyl–2–hexenoic acid_g_2
EXPNO 2
PROCNO 1
F2 – Acquisition Parameters
Date_ 20161208
Time 3.23
INSTRUM spect
PROBHD 5 mm PABBO BB–
PULPROG zgpg30
TD 51200
SOLVENT CDCl3
NS 512
DS 0
SWH 24038.461 Hz
FIDRES 0.469501 Hz
AQ 1.0650100 sec
RG 64
DW 20.800 usec
DE 6.00 usec
TE 300.0 K
D1 1.00000000 sec
d11 0.03000000 sec
DELTA 0.89999998 sec
TD0 1
======== CHANNEL f1 ========
NUC1 13C
P1 7.40 usec
PL1 –3.00 dB
SFO1 100.6706101 MHz
======== CHANNEL f2 ========
CPDPRG2 waltz16
NUC2 1H
PCPD2 90.00 usec
PL2 –4.00 dB
PL12 13.72 dB
PL13 18.01 dB
SFO2 400.3216013 MHz
F2 – Processing parameters
SI 65536
SF 100.6605292 MHz
WDW EM
SSB 0
LB 1.00 Hz
GB 0
PC 1.40
200
180
160
140
120
100
80
60
40
20
0
ppm

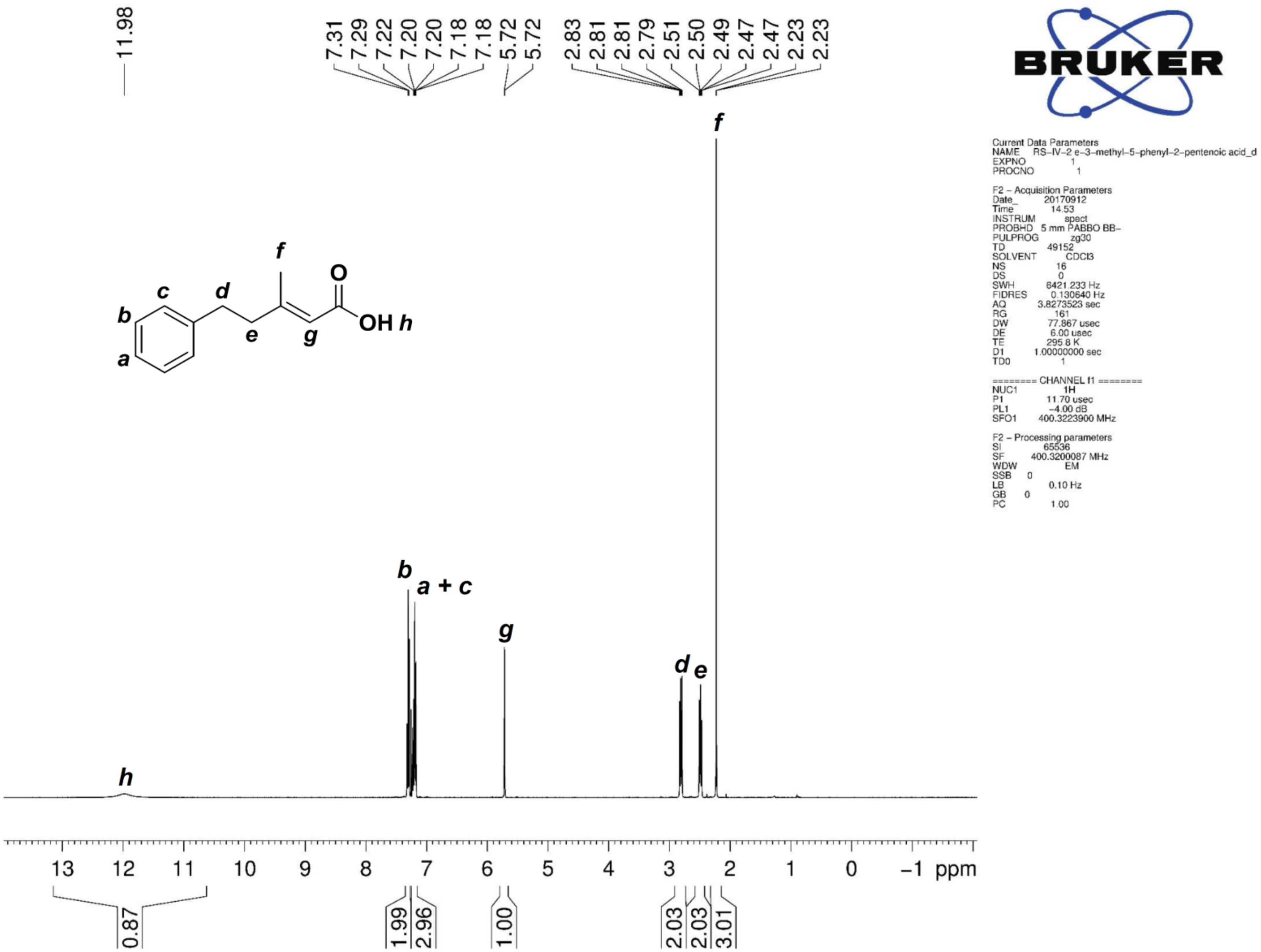
11.98
7.31 7.29 7.22 7.20 7.20 7.18 7.18 5.72 5.72
2.83 2.81 2.81 2.79 2.51 2.50 2.49 2.47 2.47 2.23 2.23
BRUKER
f
Current Data Parameters
NAME RS–IV–2 e–3–methyl–5–phenyl–2–pentenoic acid_d
EXPNO 1
PROCNO 1
F2 – Acquisition Parameters
Date_ 20170912
Time 14.53
INSTRUM spect
PROBHD 5 mm PABBO BB–
PULPROG zg30
TD 49152
SOLVENT CDCl3
NS 16
DS 0
SWH 6421.233 Hz
FIDRES 0.130640 Hz
AQ 3.8273523 sec
RG 161
DW 77.867 usec
DE 6.00 usec
TE 295.8 K
D1 1.00000000 sec
TD0 1
======== CHANNEL f1 ========
NUC1 1H
P1 11.70 usec
PL1 –4.00 dB
SFO1 400.3223900 MHz
F2 – Processing parameters
SI 65536
SF 400.3200087 MHz
WDW EM
SSB 0
LB 0.10 Hz
GB 0
PC 1.00
f
c
d
O
b
OH h
e
g
a
b
a + c
g
d e
h
13 12 11 10 9 8 7 6 5 4 3 2 1 0 –1 ppm
0.87
1.99 2.96
1.00
2.03 2.03 3.01

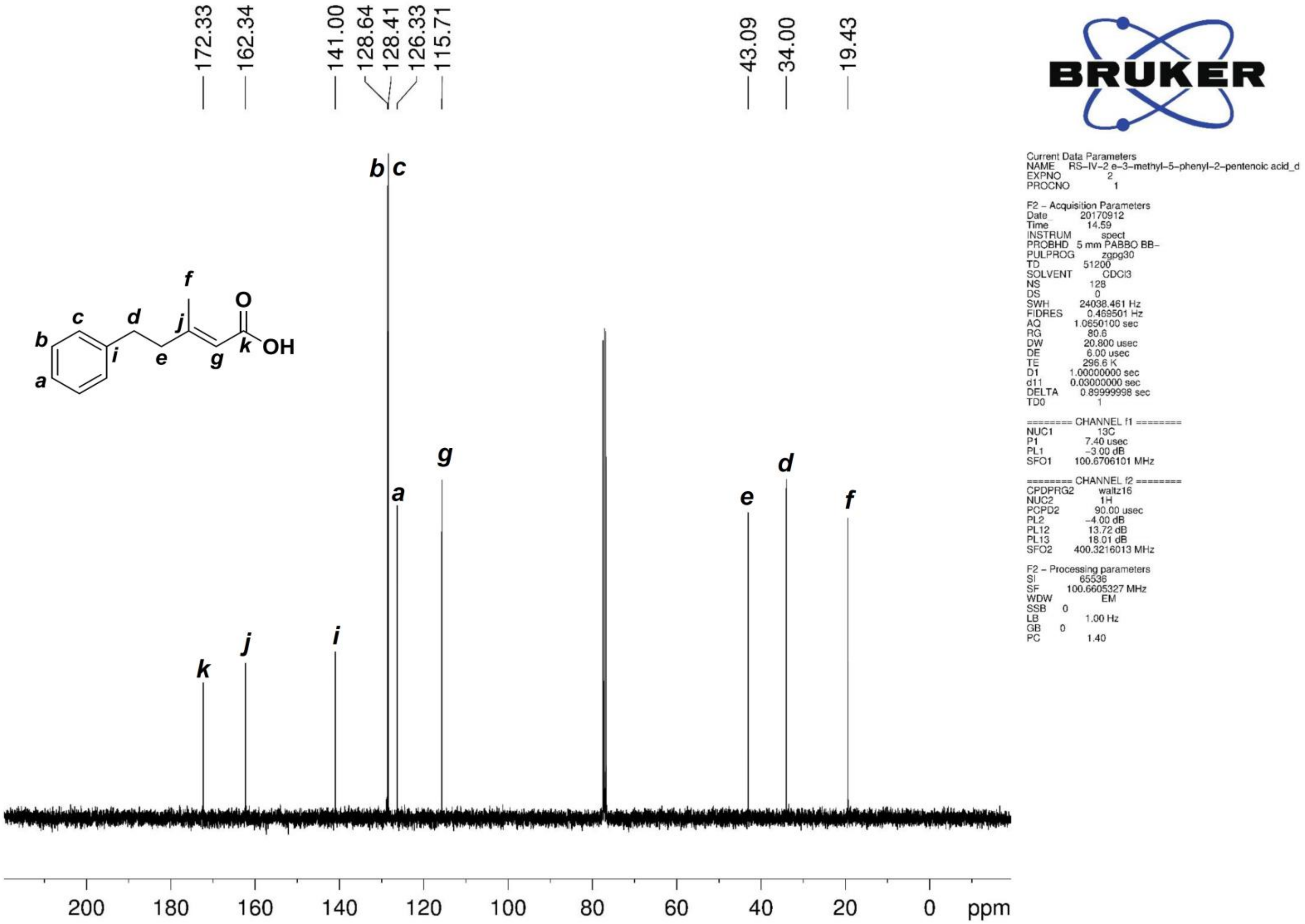

172.33
162.34
141.00
128.64
128.41
126.33
115.71
43.09
34.00
19.43
b
c
a
g
e
d
f
k
j
i
f
O
d
j
c
b
i
k
OH
e
g
a
200
180
160
140
120
100
80
60
40
20
0
ppm
BRUKER
Current Data Parameters
NAME RS–IV–2 e–3–methyl–5–phenyl–2–pentenoic acid_d
EXPNO 2
PROCNO 1
F2 – Acquisition Parameters
Date_ 20170912
Time 14.59
INSTRUM spect
PROBHD 5 mm PABBO BB–
PULPROG zgpg30
TD 51200
SOLVENT CDCl3
NS 128
DS 0
SWH 24038.461 Hz
FIDRES 0.469501 Hz
AQ 1.0650100 sec
RG 80.6
DW 20.800 usec
DE 6.00 usec
TE 296.6 K
D1 1.00000000 sec
d11 0.03000000 sec
DELTA 0.89999998 sec
TD0 1
======== CHANNEL f1 ========
NUC1 13C
P1 7.40 usec
PL1 –3.00 dB
SFO1 100.6706101 MHz
======== CHANNEL f2 ========
CPDPRG2 waltz16
NUC2 1H
PCPD2 90.00 usec
PL2 –4.00 dB
PL12 13.72 dB
PL13 18.01 dB
SFO2 400.3216013 MHz
F2 – Processing parameters
SI 65536
SF 100.6605327 MHz
WDW EM
SSB 0
LB 1.00 Hz
GB 0
PC 1.40

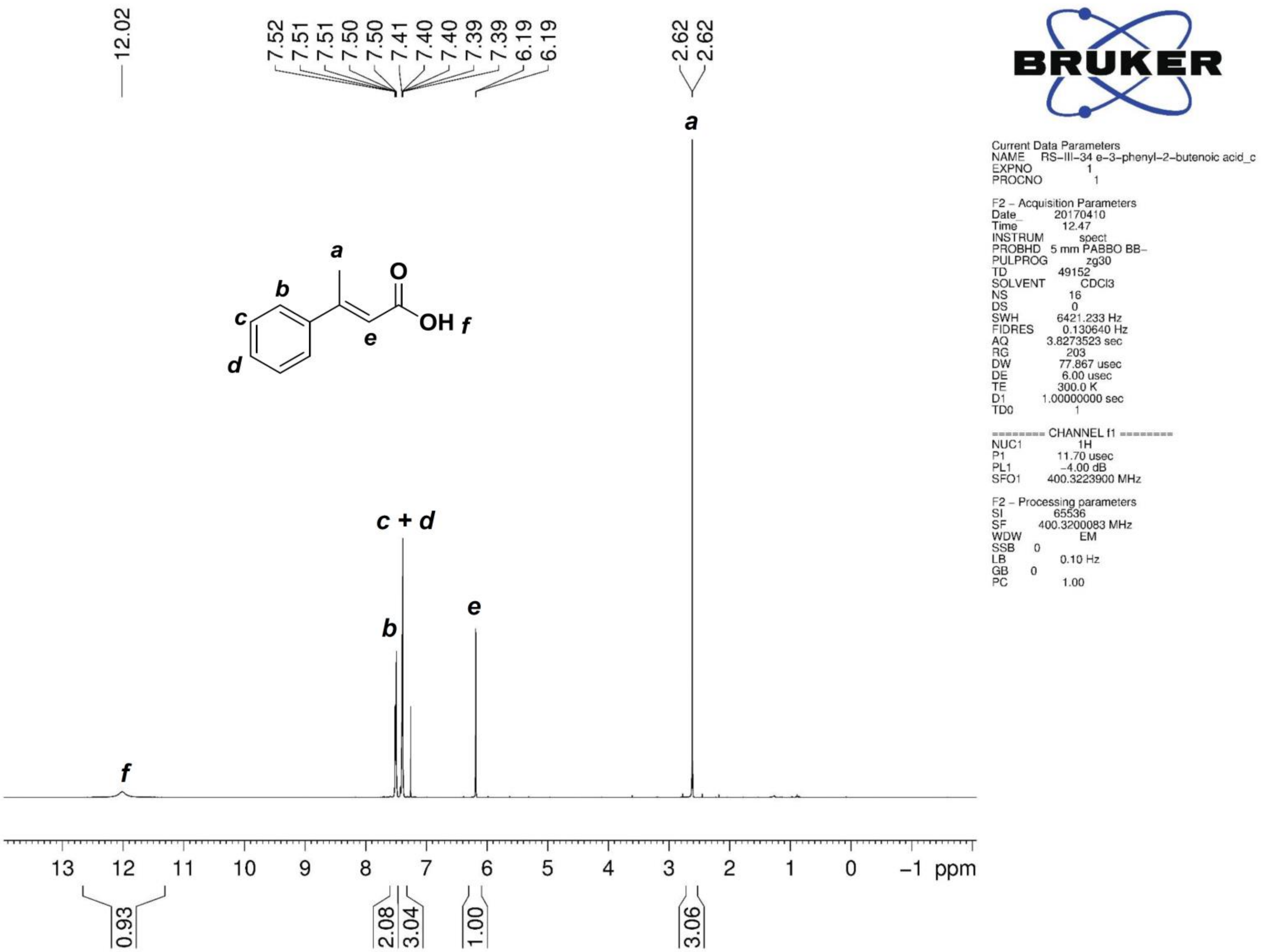
12.02
7.52
7.51
7.51
7.50
7.50
7.41
7.40
7.40
7.39
7.39
6.19
6.19
2.62
2.62
a
b
c
d
e
f
O
OH
c + d
BRUKER
Current Data Parameters
NAME RS-III-34 e-3-phenyl-2-butenoic acid_c
EXPNO 1
PROCNO 1
F2 - Acquisition Parameters
Date_ 20170410
Time 12.47
INSTRUM spect
PROBHD 5 mm PABBO BB-
PULPROG zg30
TD 49152
SOLVENT CDCl3
NS 16
DS 0
SWH 6421.233 Hz
FIDRES 0.130640 Hz
AQ 3.8273523 sec
RG 203
DW 77.867 usec
DE 6.00 usec
TE 300.0 K
D1 1.00000000 sec
TD0 1
======== CHANNEL f1 ========
NUC1 1H
P1 11.70 usec
PL1 -4.00 dB
SFO1 400.3223900 MHz
F2 - Processing parameters
SI 65536
SF 400.3200083 MHz
WDW EM
SSB 0
LB 0.10 Hz
GB 0
PC 1.00
13 12 11 10 9 8 7 6 5 4 3 2 1 0 -1 ppm
0.93
2.08
3.04
1.00
3.06

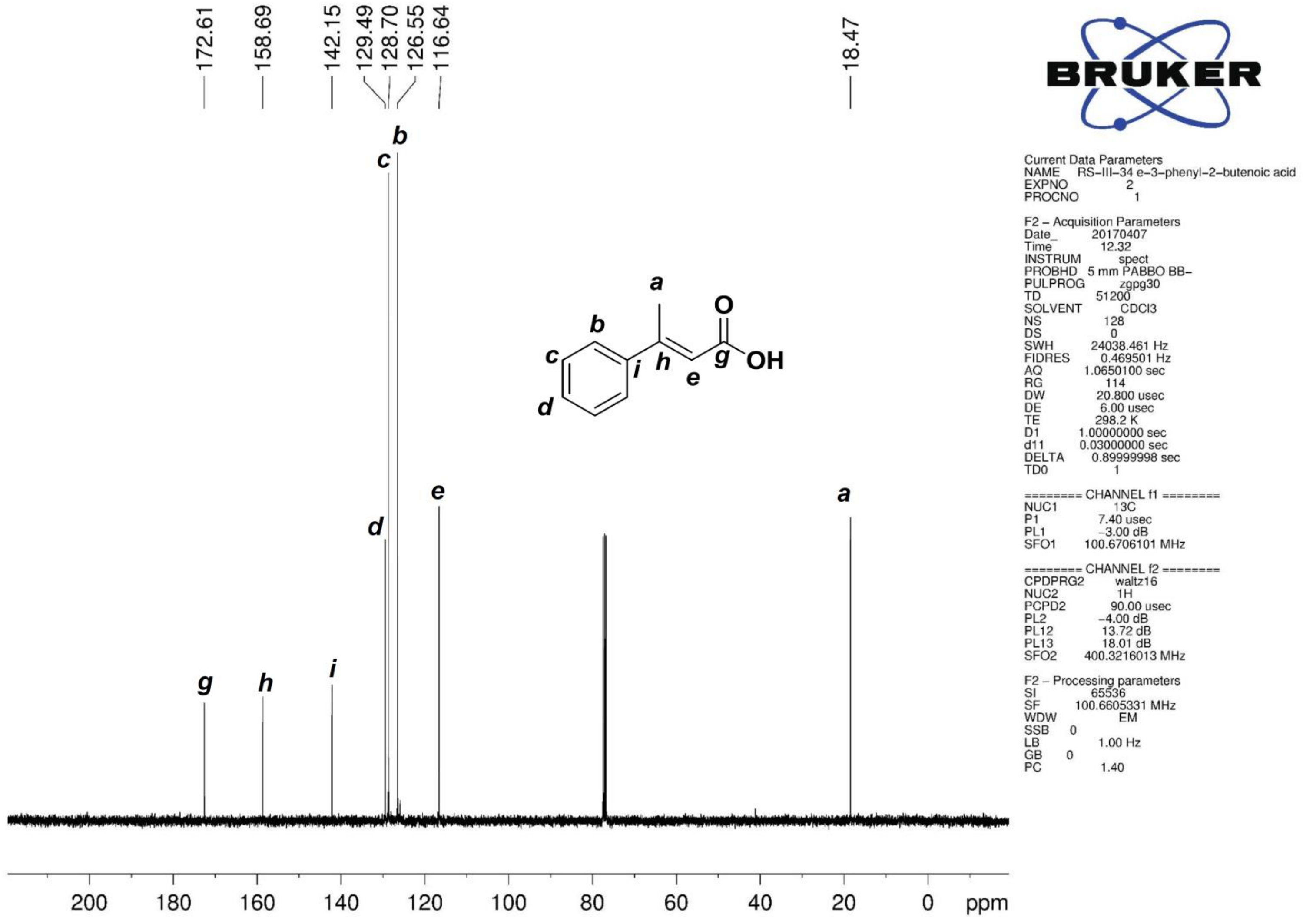
172.61
158.69
142.15
129.49
128.70
126.55
116.64
18.47
a
b
c
d
e
g
h
i
O
OH
200
180
160
140
120
100
80
60
40
20
0
ppm
Current Data Parameters
NAME RS-III-34 e-3-phenyl-2-butenoic acid
EXPNO 2
PROCNO 1
F2 - Acquisition Parameters
Date_ 20170407
Time 12.32
INSTRUM spect
PROBHD 5 mm PABBO BB-
PULPROG zgpg30
TD 51200
SOLVENT CDCl3
NS 128
DS 0
SWH 24038.461 Hz
FIDRES 0.469501 Hz
AQ 1.0650100 sec
RG 114
DW 20.800 usec
DE 6.00 usec
TE 298.2 K
D1 1.00000000 sec
d11 0.03000000 sec
DELTA 0.89999998 sec
TD0 1
======== CHANNEL f1 ========
NUC1 13C
P1 7.40 usec
PL1 -3.00 dB
SFO1 100.6706101 MHz
======== CHANNEL f2 ========
CPDPRG2 waltz16
NUC2 1H
PCPD2 90.00 usec
PL2 -4.00 dB
PL12 13.72 dB
PL13 18.01 dB
SFO2 400.3216013 MHz
F2 - Processing parameters
SI 65536
SF 100.6605331 MHz
WDW EM
SSB 0
LB 1.00 Hz
GB 0
PC 1.40

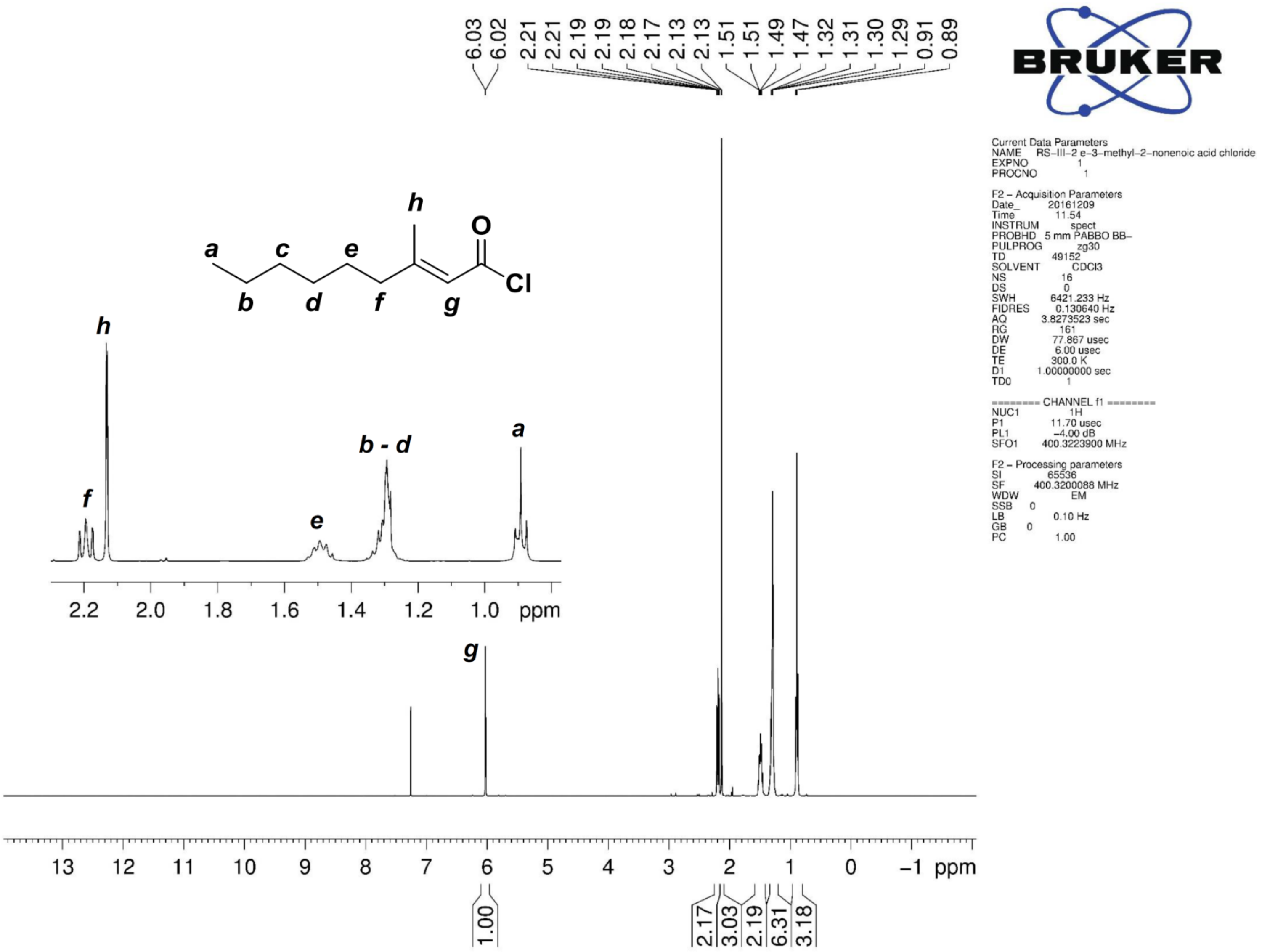

6.03
6.02
2.21
2.21
2.19
2.19
2.18
2.17
2.13
2.13
1.51
1.51
1.49
1.47
1.32
1.31
1.30
1.29
0.91
0.89
h
O
a
c
e
Cl
b
d
f
g
h
f
e
b - d
a
2.2
2.0
1.8
1.6
1.4
1.2
1.0
ppm
g
13
12
11
10
9
8
7
6
5
4
3
2
1
0
−1
ppm
1.00
2.17
3.03
2.19
6.31
3.18
Current Data Parameters
NAME RS–III–2 e–3–methyl–2–nonenoic acid chloride
EXPNO 1
PROCNO 1
F2 – Acquisition Parameters
Date_ 20161209
Time 11.54
INSTRUM spect
PROBHD 5 mm PABBO BB–
PULPROG zg30
TD 49152
SOLVENT CDCl3
NS 16
DS 0
SWH 6421.233 Hz
FIDRES 0.130640 Hz
AQ 3.8273523 sec
RG 161
DW 77.867 usec
DE 6.00 usec
TE 300.0 K
D1 1.00000000 sec
TD0 1
======== CHANNEL f1 ========
NUC1 1H
P1 11.70 usec
PL1 −4.00 dB
SFO1 400.3223900 MHz
F2 – Processing parameters
SI 65536
SF 400.3200088 MHz
WDW EM
SSB 0
LB 0.10 Hz
GB 0
PC 1.00

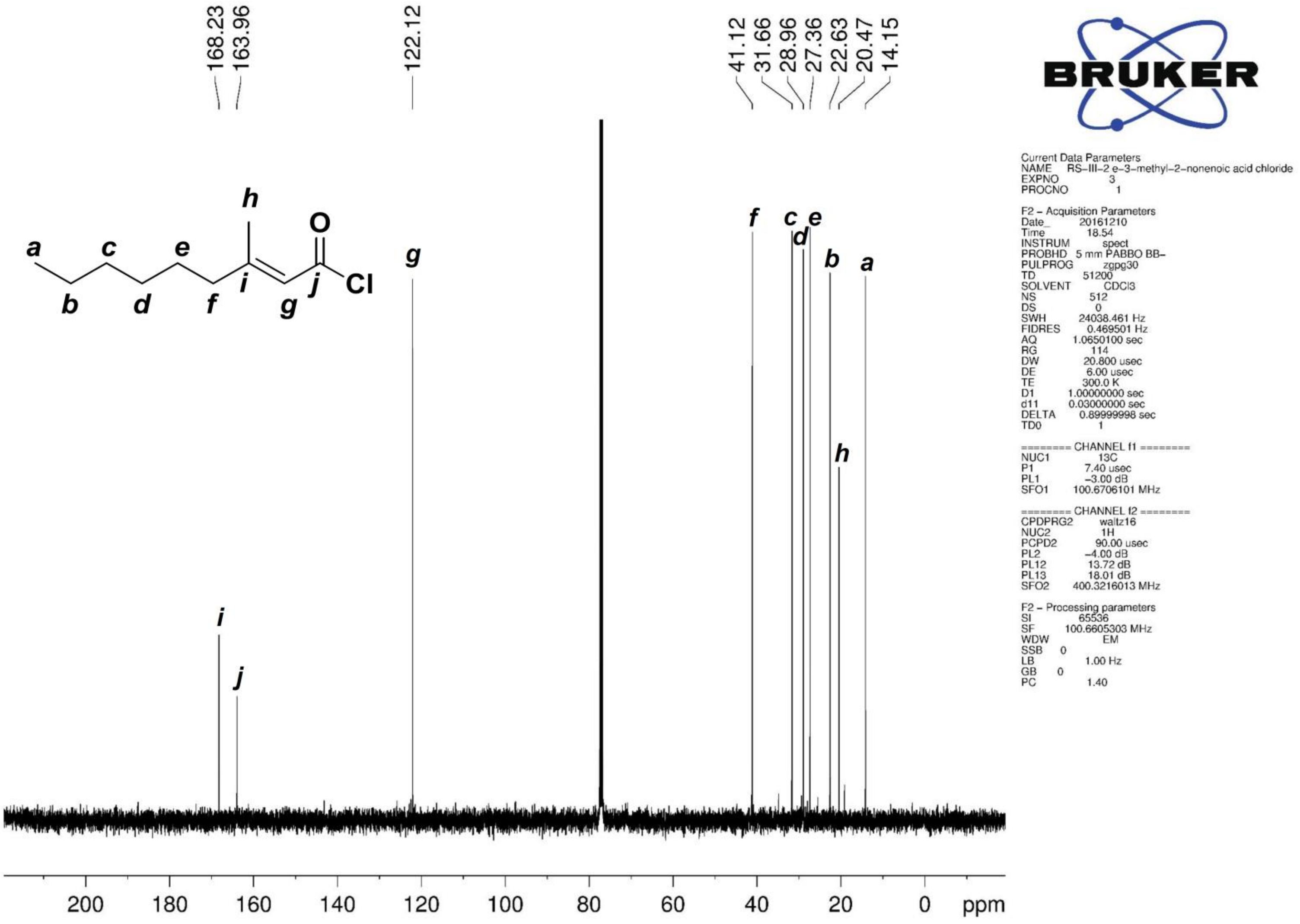
168.23
163.96
122.12
41.12
31.66
28.96
27.36
22.63
20.47
14.15
BRUKER
Current Data Parameters
NAME RS–III–2 e–3–methyl–2–nonenoic acid chloride
EXPNO 3
PROCNO 1
F2 – Acquisition Parameters
Date_ 20161210
Time 18.54
INSTRUM spect
PROBHD 5 mm PABBO BB–
PULPROG zgpg30
TD 51200
SOLVENT CDCl3
NS 512
DS 0
SWH 24038.461 Hz
FIDRES 0.469501 Hz
AQ 1.0650100 sec
RG 114
DW 20.800 usec
DE 6.00 usec
TE 300.0 K
D1 1.00000000 sec
d11 0.03000000 sec
DELTA 0.89999998 sec
TD0 1
======== CHANNEL f1 ========
NUC1 13C
P1 7.40 usec
PL1 –3.00 dB
SFO1 100.6706101 MHz
======== CHANNEL f2 ========
CPDPRG2 waltz16
NUC2 1H
PCPD2 90.00 usec
PL2 –4.00 dB
PL12 13.72 dB
PL13 18.01 dB
SFO2 400.3216013 MHz
F2 – Processing parameters
SI 65536
SF 100.6605303 MHz
WDW EM
SSB 0
LB 1.00 Hz
GB 0
PC 1.40
h
O
a
c
e
Cl
b
d
f
i
g
j
200
180
160
140
120
100
80
60
40
20
0
ppm

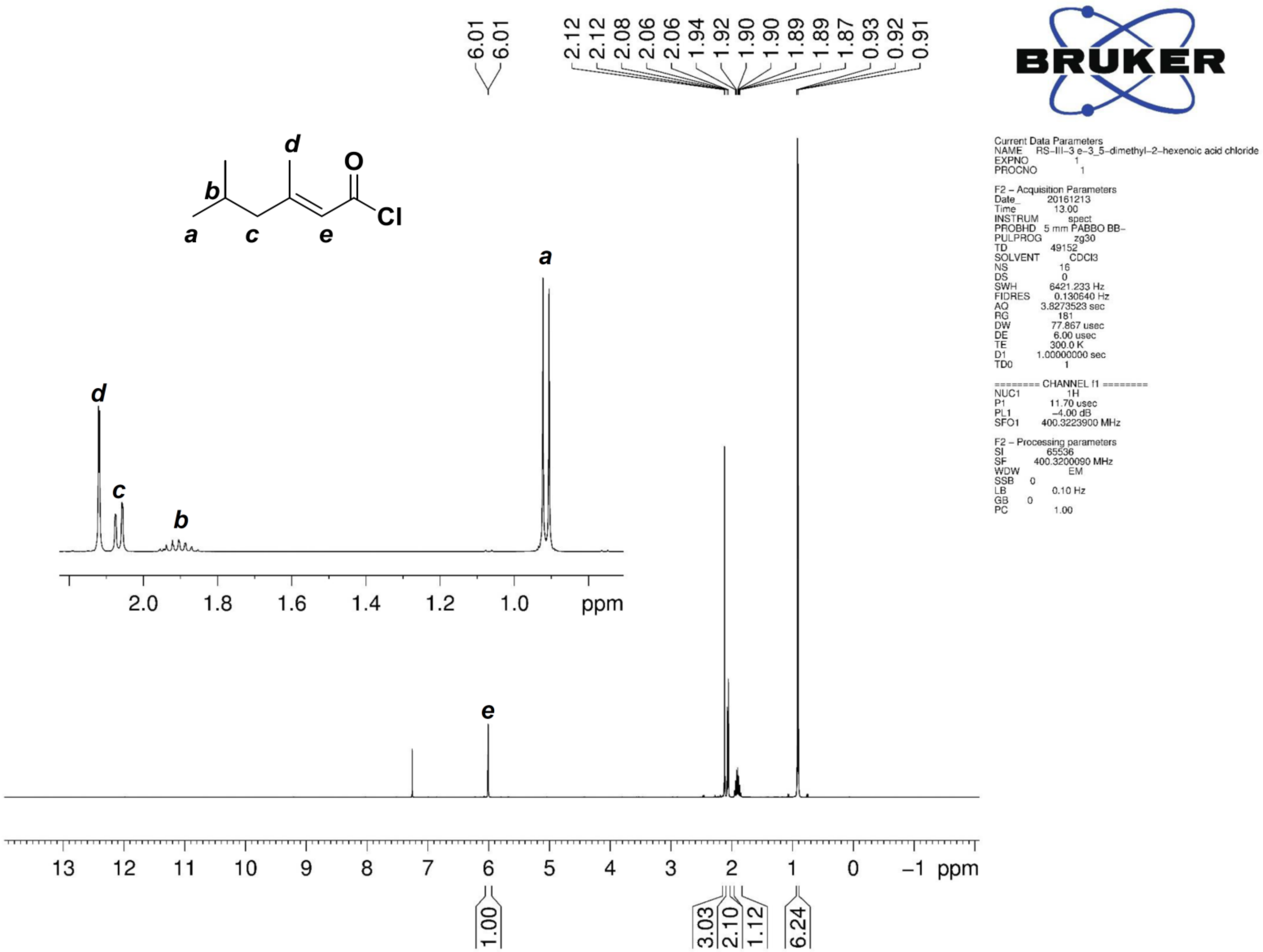
6.01
6.01
2.12
2.12
2.08
2.06
2.06
1.94
1.92
1.90
1.90
1.89
1.89
1.87
0.93
0.92
0.91
d
O
b
Cl
a
c
e
a
d
c
b
2.0
1.8
1.6
1.4
1.2
1.0
ppm
e
13
12
11
10
9
8
7
6
5
4
3
2
1
0
−1
ppm
1.00
3.03
2.10
1.12
6.24
Current Data Parameters
NAME RS–III–3 e–3_5–dimethyl–2–hexenoic acid chloride
EXPNO 1
PROCNO 1
F2 – Acquisition Parameters
Date_ 20161213
Time 13.00
INSTRUM spect
PROBHD 5 mm PABBO BB–
PULPROG zg30
TD 49152
SOLVENT CDCl3
NS 16
DS 0
SWH 6421.233 Hz
FIDRES 0.130640 Hz
AQ 3.8273523 sec
RG 181
DW 77.867 usec
DE 6.00 usec
TE 300.0 K
D1 1.00000000 sec
TD0 1
======== CHANNEL f1 ========
NUC1 1H
P1 11.70 usec
PL1 −4.00 dB
SFO1 400.3223900 MHz
F2 – Processing parameters
SI 65536
SF 400.3200090 MHz
WDW EM
SSB 0
LB 0.10 Hz
GB 0
PC 1.00

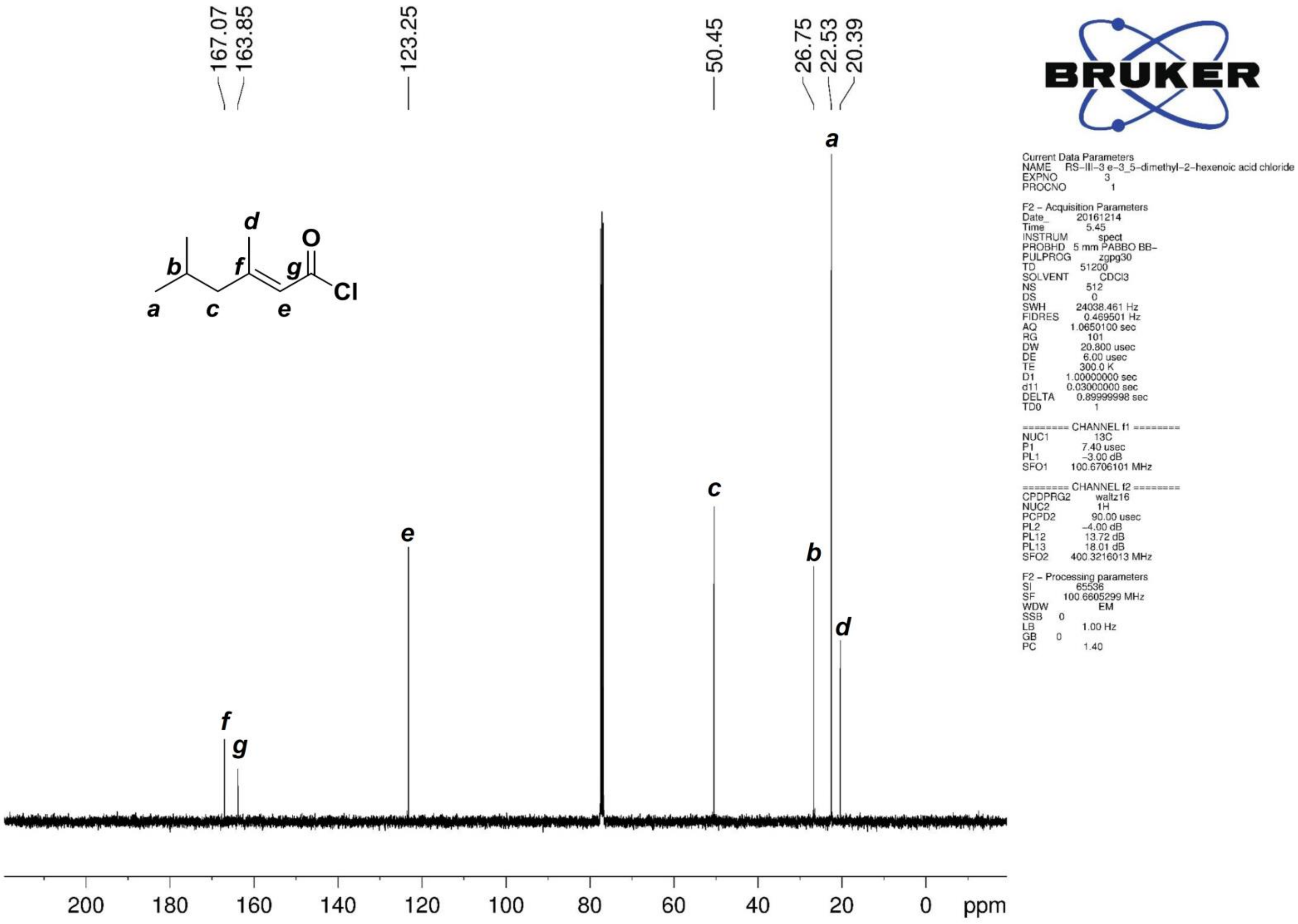
167.07
163.85
123.25
50.45
26.75
22.53
20.39
BRUKER
a
b
c
d
e
f
g
O
Cl
Current Data Parameters
NAME RS-III-3 e-3_5-dimethyl-2-hexenoic acid chloride
EXPNO 3
PROCNO 1
F2 - Acquisition Parameters
Date_ 20161214
Time 5.45
INSTRUM spect
PROBHD 5 mm PABBO BB-
PULPROG zgpg30
TD 51200
SOLVENT CDCl3
NS 512
DS 0
SWH 24038.461 Hz
FIDRES 0.469501 Hz
AQ 1.0650100 sec
RG 101
DW 20.800 usec
DE 6.00 usec
TE 300.0 K
D1 1.00000000 sec
d11 0.03000000 sec
DELTA 0.89999998 sec
TD0 1
======== CHANNEL f1 ========
NUC1 13C
P1 7.40 usec
PL1 -3.00 dB
SFO1 100.6706101 MHz
======== CHANNEL f2 ========
CPDPRG2 waltz16
NUC2 1H
PCPD2 90.00 usec
PL2 -4.00 dB
PL12 13.72 dB
PL13 18.01 dB
SFO2 400.3216013 MHz
F2 - Processing parameters
SI 65536
SF 100.6605299 MHz
WDW EM
SSB 0
LB 1.00 Hz
GB 0
PC 1.40
200
180
160
140
120
100
80
60
40
20
0
ppm

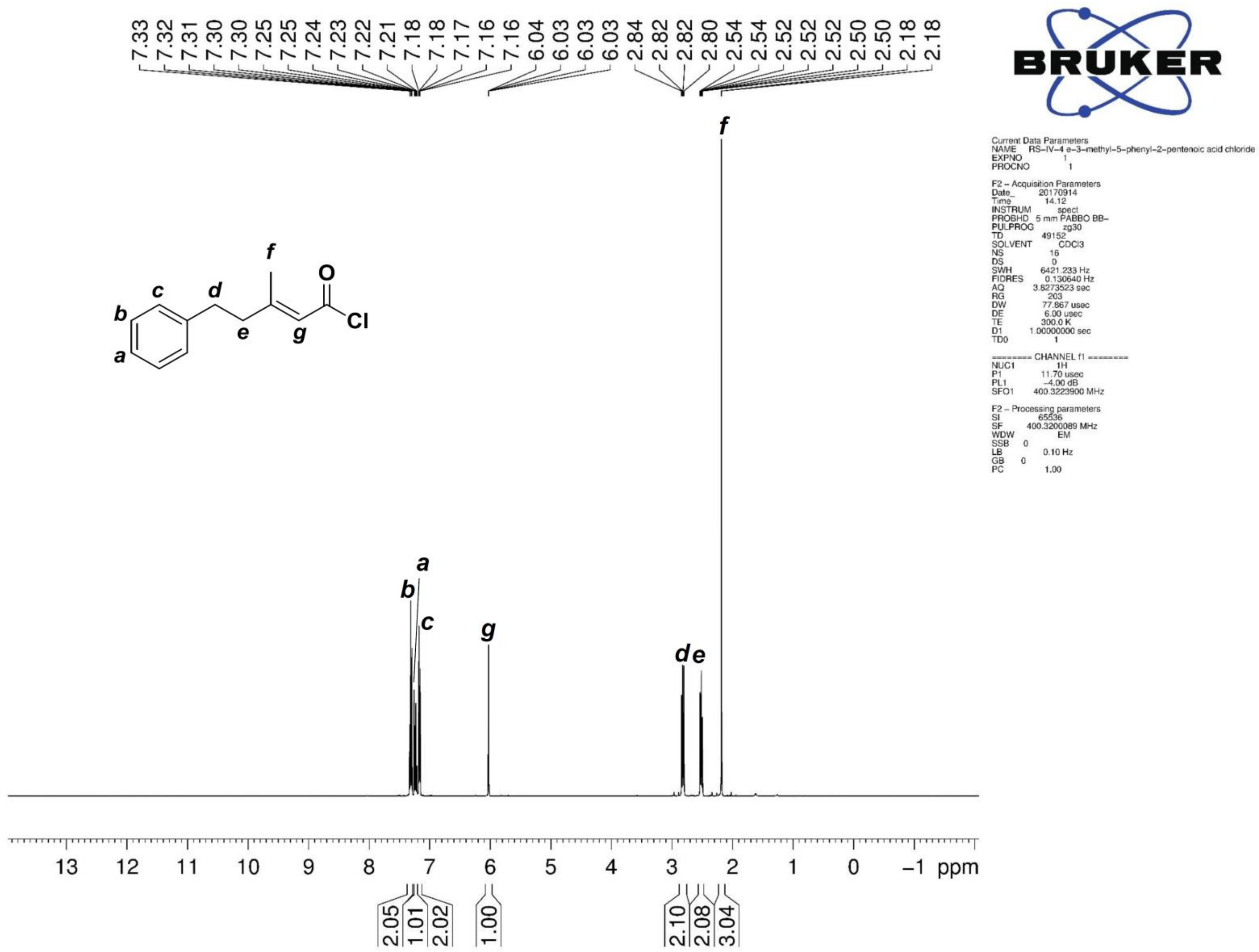

7.33 7.32 7.31 7.30 7.30 7.25 7.25 7.24 7.23 7.22 7.21 7.18 7.18 7.17 7.16 7.16 6.04 6.03 6.03 6.03 2.84 2.82 2.82 2.80 2.54 2.54 2.52 2.52 2.52 2.50 2.50 2.18 2.18
BRUKER
Current Data Parameters
NAME RS-IV-4 e-3-methyl-5-phenyl-2-pentenoic acid chloride
EXPNO 1
PROCNO 1
F2 – Acquisition Parameters
Date_ 20170914
Time 14.12
INSTRUM spect
PROBHD 5 mm PABBO BB-
PULPROG zg30
TD 49152
SOLVENT CDCl3
NS 16
DS 0
SWH 6421.233 Hz
FIDRES 0.130640 Hz
AQ 3.8273523 sec
RG 203
DW 77.867 usec
DE 6.00 usec
TE 300.0 K
D1 1.00000000 sec
TD0 1
======== CHANNEL f1 ========
NUC1 1H
P1 11.70 usec
PL1 -4.00 dB
SFO1 400.3223900 MHz
F2 – Processing parameters
SI 65536
SF 400.3200089 MHz
WDW EM
SSB 0
LB 0.10 Hz
GB 0
PC 1.00
a b c d e f g O Cl
13 12 11 10 9 8 7 6 5 4 3 2 1 0 −1 ppm
2.05 1.01 2.02 1.00 2.10 2.08 3.04

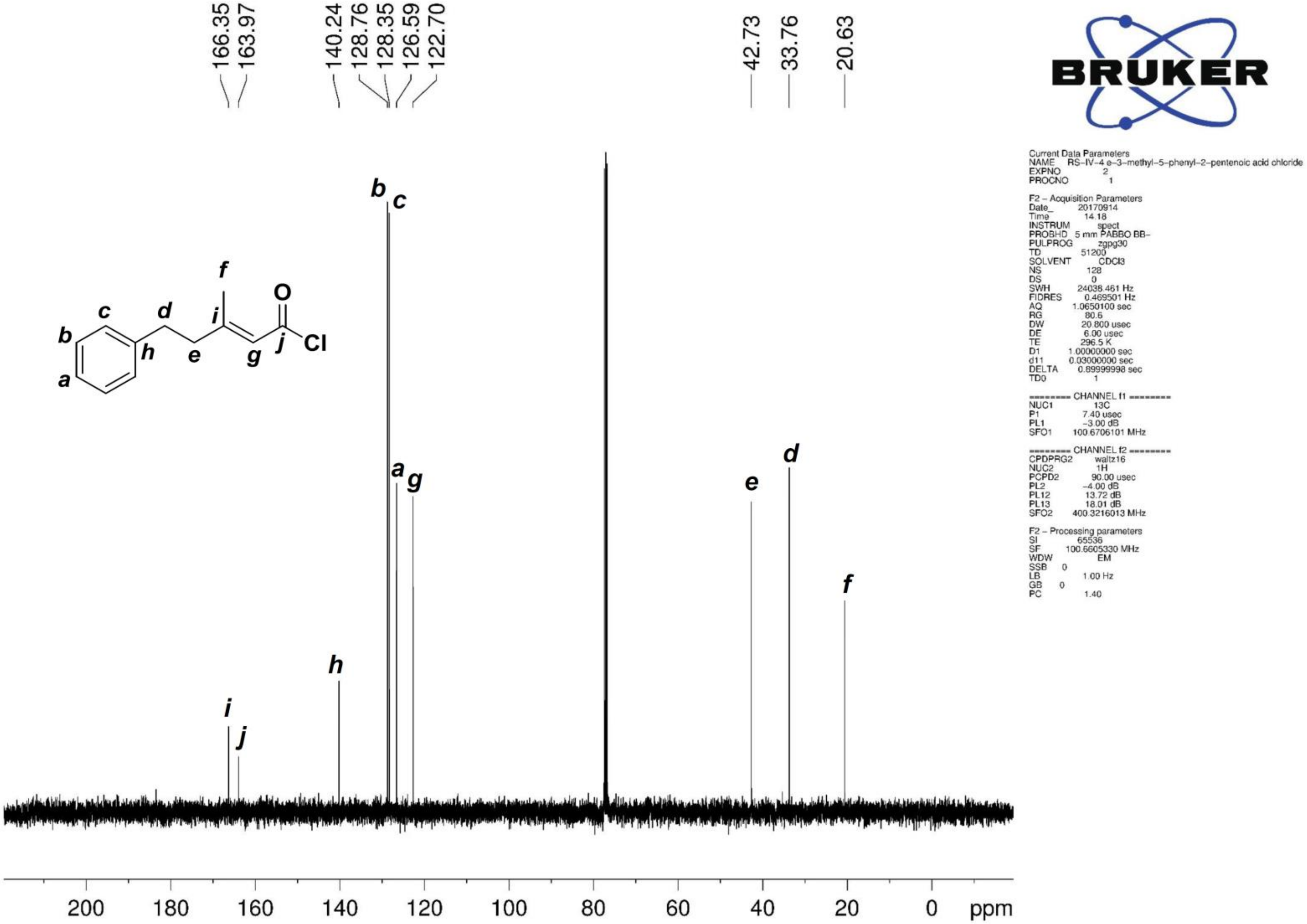
166.35
163.97
140.24
128.76
128.35
126.59
122.70
42.73
33.76
20.63
O
Cl
a
b
c
d
e
f
g
h
i
j
BRUKER
Current Data Parameters
NAME RS-IV-4 e-3-methyl-5-phenyl-2-pentenoic acid chloride
EXPNO 2
PROCNO 1
F2 - Acquisition Parameters
Date_ 20170914
Time 14.18
INSTRUM spect
PROBHD 5 mm PABBO BB-
PULPROG zgpg30
TD 51200
SOLVENT CDCl3
NS 128
DS 0
SWH 24038.461 Hz
FIDRES 0.469501 Hz
AQ 1.0650100 sec
RG 80.6
DW 20.800 usec
DE 6.00 usec
TE 296.5 K
D1 1.00000000 sec
d11 0.03000000 sec
DELTA 0.89999998 sec
TD0 1
======== CHANNEL f1 ========
NUC1 13C
P1 7.40 usec
PL1 -3.00 dB
SFO1 100.6706101 MHz
======== CHANNEL f2 ========
CPDPRG2 waltz16
NUC2 1H
PCPD2 90.00 usec
PL2 -4.00 dB
PL12 13.72 dB
PL13 18.01 dB
SFO2 400.3216013 MHz
F2 - Processing parameters
SI 65536
SF 100.6605330 MHz
WDW EM
SSB 0
LB 1.00 Hz
GB 0
PC 1.40
200
180
160
140
120
100
80
60
40
20
0
ppm

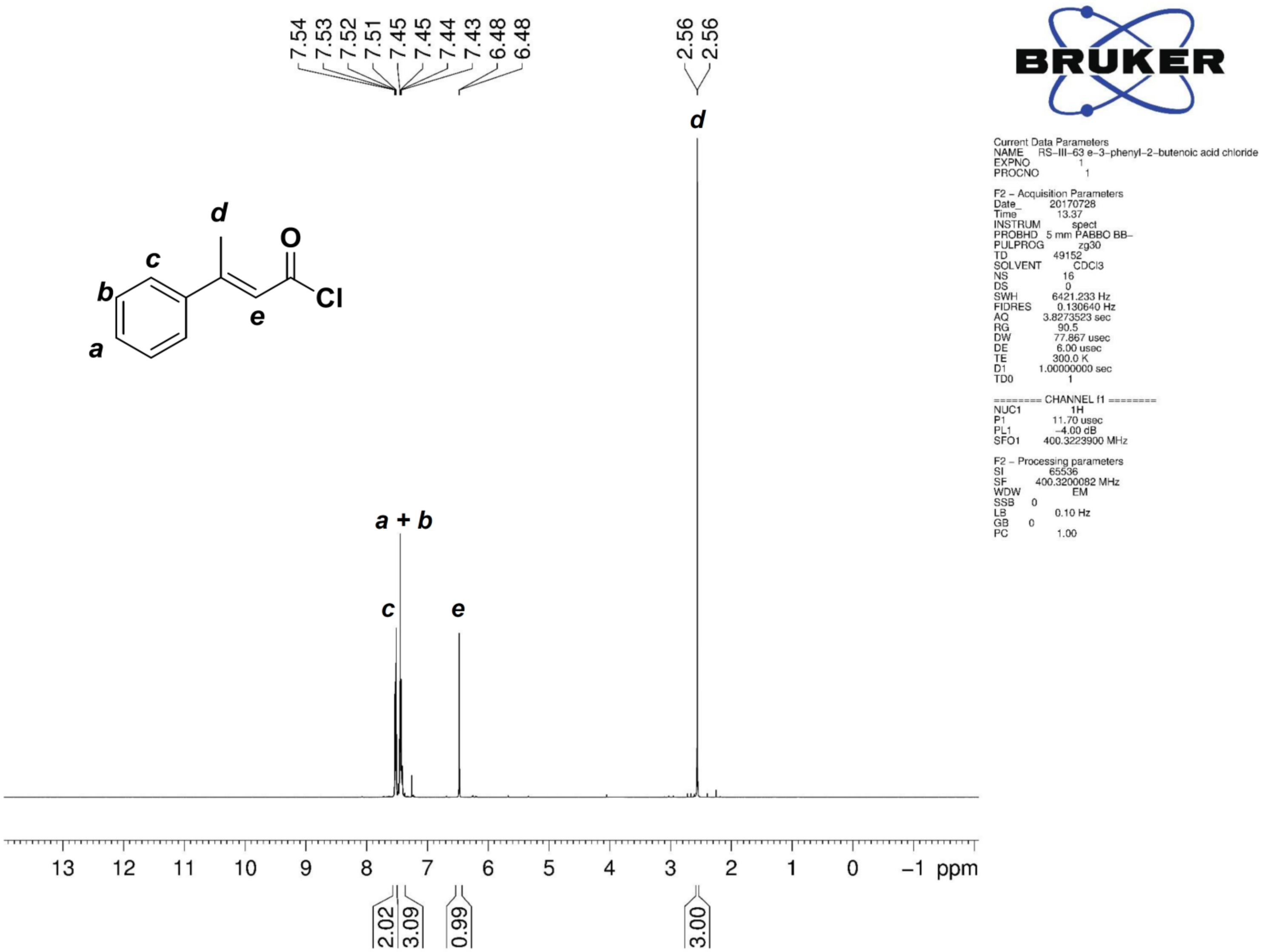

7.54
7.53
7.52
7.51
7.45
7.45
7.44
7.43
6.48
6.48
2.56
2.56
d
a + b
c
e
2.02
3.09
0.99
3.00
13 12 11 10 9 8 7 6 5 4 3 2 1 0 −1 ppm
O
Cl
a
b
c
d
e
BRUKER
Current Data Parameters
NAME RS–III–63 e–3–phenyl–2–butenoic acid chloride
EXPNO 1
PROCNO 1
F2 – Acquisition Parameters
Date_ 20170728
Time 13.37
INSTRUM spect
PROBHD 5 mm PABBO BB–
PULPROG zg30
TD 49152
SOLVENT CDCl3
NS 16
DS 0
SWH 6421.233 Hz
FIDRES 0.130640 Hz
AQ 3.8273523 sec
RG 90.5
DW 77.867 usec
DE 6.00 usec
TE 300.0 K
D1 1.00000000 sec
TD0 1
======== CHANNEL f1 ========
NUC1 1H
P1 11.70 usec
PL1 −4.00 dB
SFO1 400.3223900 MHz
F2 – Processing parameters
SI 65536
SF 400.3200082 MHz
WDW EM
SSB 0
LB 0.10 Hz
GB 0
PC 1.00

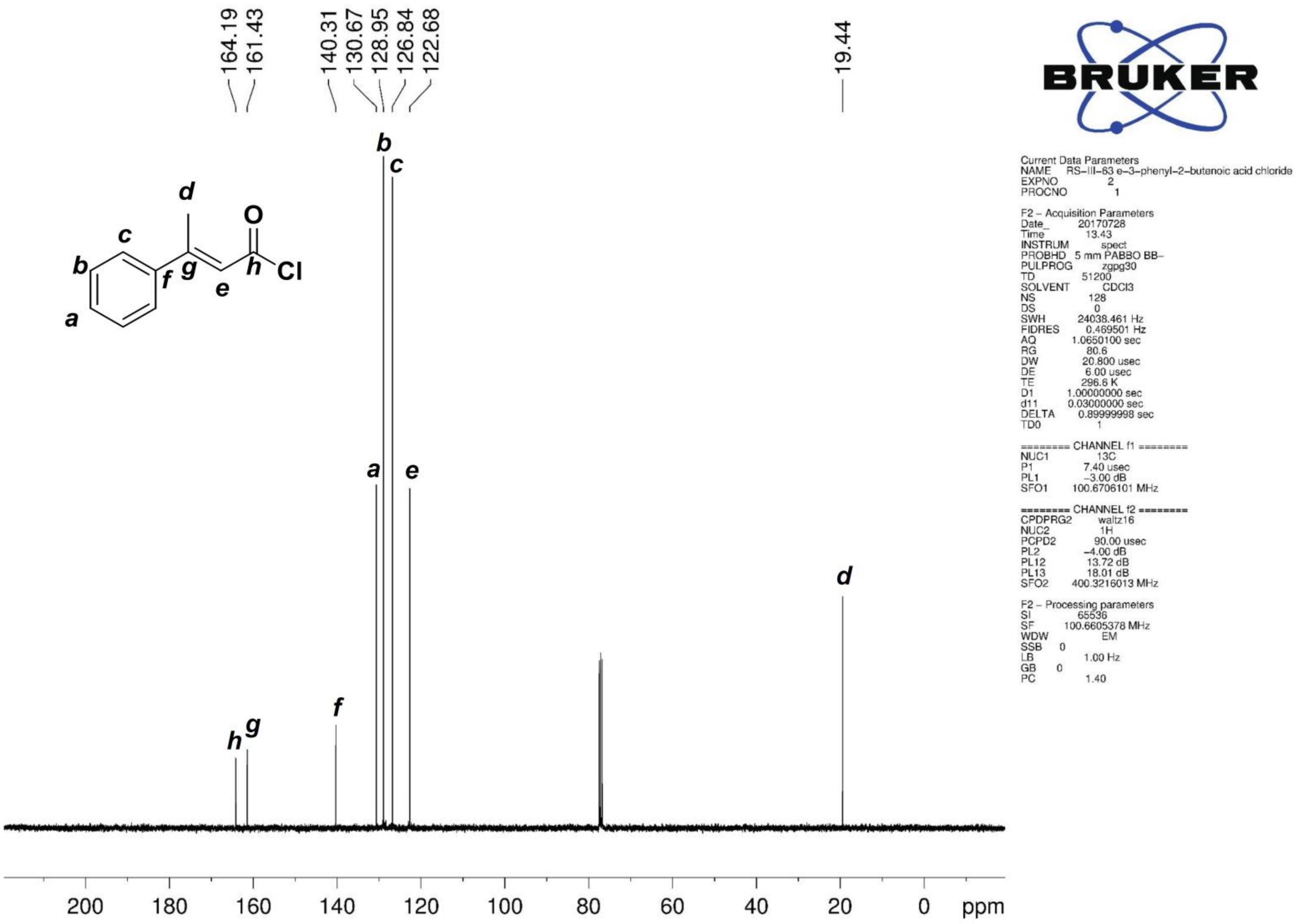

164.19
161.43
140.31
130.67
128.95
126.84
122.68
19.44
BRUKER
Current Data Parameters
NAME RS–III–63 e–3–phenyl–2–butenoic acid chloride
EXPNO 2
PROCNO 1
F2 – Acquisition Parameters
Date_ 20170728
Time 13.43
INSTRUM spect
PROBHD 5 mm PABBO BB–
PULPROG zgpg30
TD 51200
SOLVENT CDCl3
NS 128
DS 0
SWH 24038.461 Hz
FIDRES 0.469501 Hz
AQ 1.0650100 sec
RG 80.6
DW 20.800 usec
DE 6.00 usec
TE 296.6 K
D1 1.00000000 sec
d11 0.03000000 sec
DELTA 0.89999998 sec
TD0 1
======== CHANNEL f1 ========
NUC1 13C
P1 7.40 usec
PL1 –3.00 dB
SFO1 100.6706101 MHz
======== CHANNEL f2 ========
CPDPRG2 waltz16
NUC2 1H
PCPD2 90.00 usec
PL2 –4.00 dB
PL12 13.72 dB
PL13 18.01 dB
SFO2 400.3216013 MHz
F2 – Processing parameters
SI 65536
SF 100.6605378 MHz
WDW EM
SSB 0
LB 1.00 Hz
GB 0
PC 1.40
200 180 160 140 120 100 80 60 40 20 0 ppm

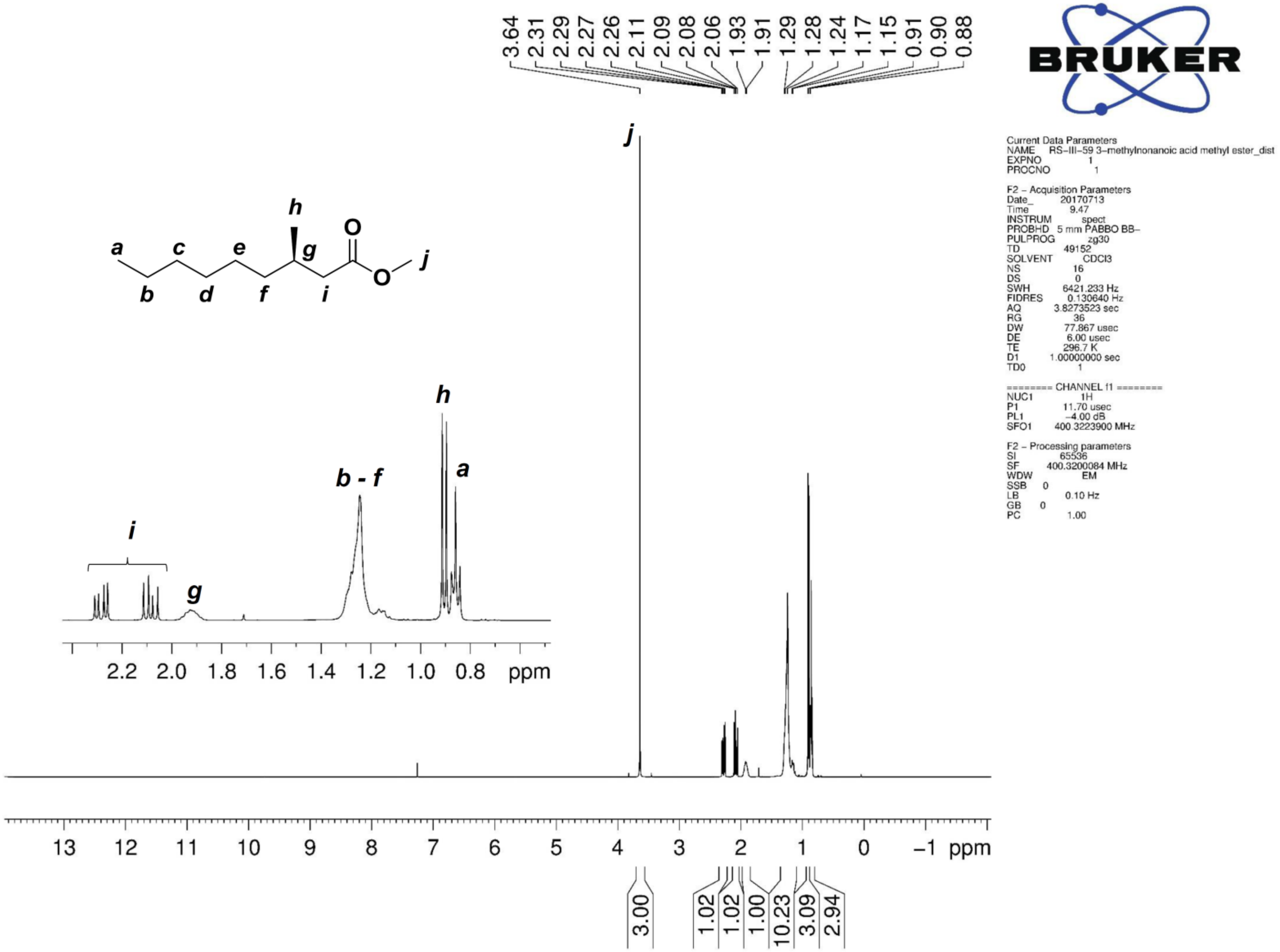

3.64 2.31 2.29 2.27 2.26 2.11 2.09 2.08 2.06 1.93 1.91 1.29 1.28 1.24 1.17 1.15 0.91 0.90 0.88
BRUKER
Current Data Parameters
NAME RS-III-59 3-methylnonanoic acid methyl ester_dist
EXPNO 1
PROCNO 1
F2 - Acquisition Parameters
Date_ 20170713
Time 9.47
INSTRUM spect
PROBHD 5 mm PABBO BB-
PULPROG zg30
TD 49152
SOLVENT CDCl3
NS 16
DS 0
SWH 6421.233 Hz
FIDRES 0.130640 Hz
AQ 3.8273523 sec
RG 36
DW 77.867 usec
DE 6.00 usec
TE 296.7 K
D1 1.00000000 sec
TD0 1
======== CHANNEL f1 ========
NUC1 1H
P1 11.70 usec
PL1 -4.00 dB
SFO1 400.3223900 MHz
F2 - Processing parameters
SI 65536
SF 400.3200084 MHz
WDW EM
SSB 0
LB 0.10 Hz
GB 0
PC 1.00
a b c d e f g h i j O O
j
h
a
b - f
i
g
2.2 2.0 1.8 1.6 1.4 1.2 1.0 0.8 ppm
13 12 11 10 9 8 7 6 5 4 3 2 1 0 −1 ppm
3.00 1.02 1.02 1.00 10.23 3.09 2.94

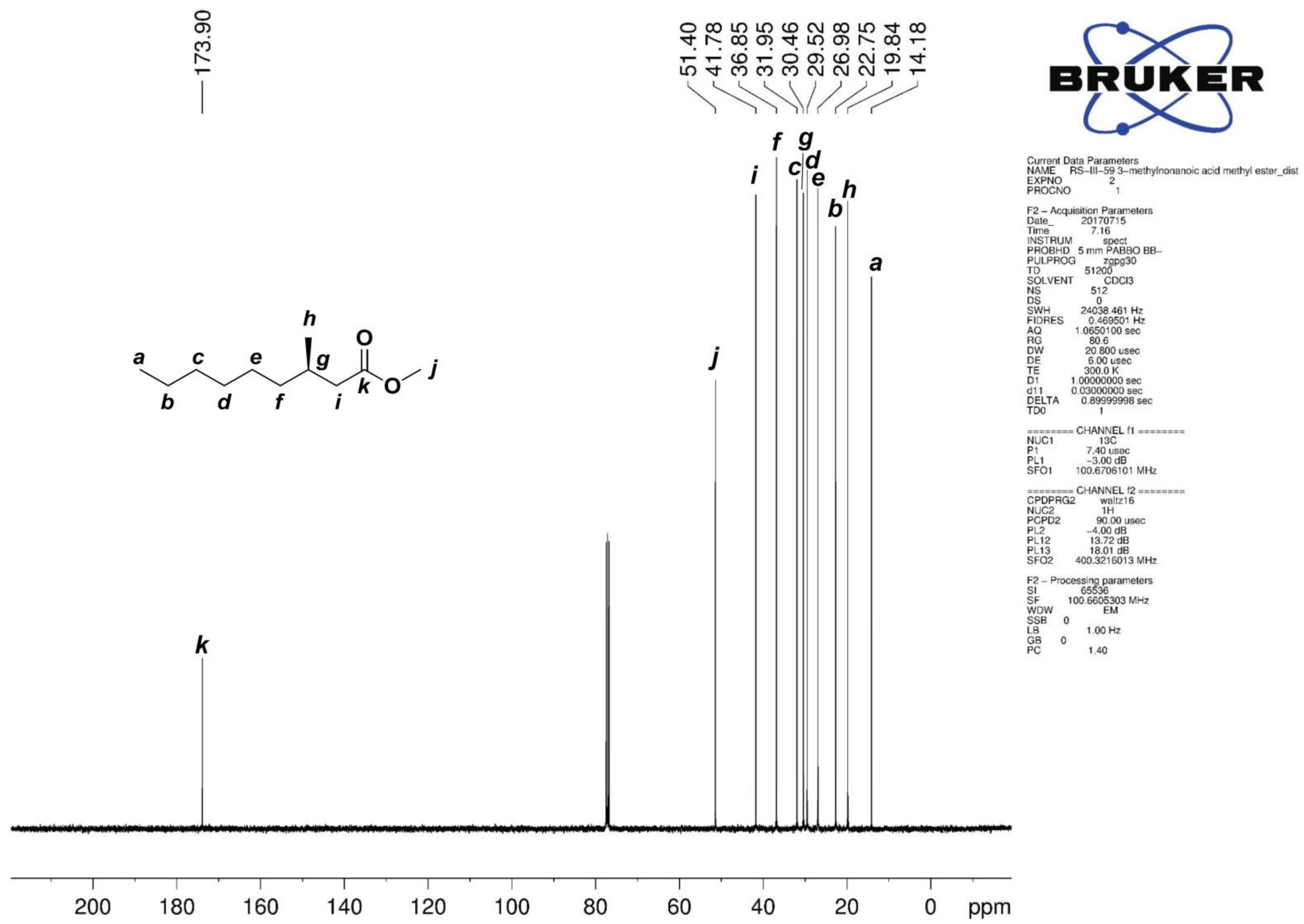

173.90
51.40
41.78
36.85
31.95
30.46
29.52
26.98
22.75
19.84
14.18
BRUKER
Current Data Parameters
NAME RS-III-59 3-methylnonanoic acid methyl ester_dist
EXPNO 2
PROCNO 1
F2 - Acquisition Parameters
Date_ 20170715
Time 7.16
INSTRUM spect
PROBHD 5 mm PABBO BB-
PULPROG zgpg30
TD 51200
SOLVENT CDCl3
NS 512
DS 0
SWH 24038.461 Hz
FIDRES 0.469501 Hz
AQ 1.0650100 sec
RG 80.6
DW 20.800 usec
DE 6.00 usec
TE 300.0 K
D1 1.00000000 sec
d11 0.03000000 sec
DELTA 0.89999998 sec
TD0 1
======== CHANNEL f1 ========
NUC1 13C
P1 7.40 usec
PL1 -3.00 dB
SFO1 100.6706101 MHz
======== CHANNEL f2 ========
CPDPRG2 waltz16
NUC2 1H
PCPD2 90.00 usec
PL2 -4.00 dB
PL12 13.72 dB
PL13 18.01 dB
SFO2 400.3216013 MHz
F2 - Processing parameters
SI 65536
SF 100.6605303 MHz
WDW EM
SSB 0
LB 1.00 Hz
GB 0
PC 1.40
a b c d e f g h i j k O
200 180 160 140 120 100 80 60 40 20 0 ppm

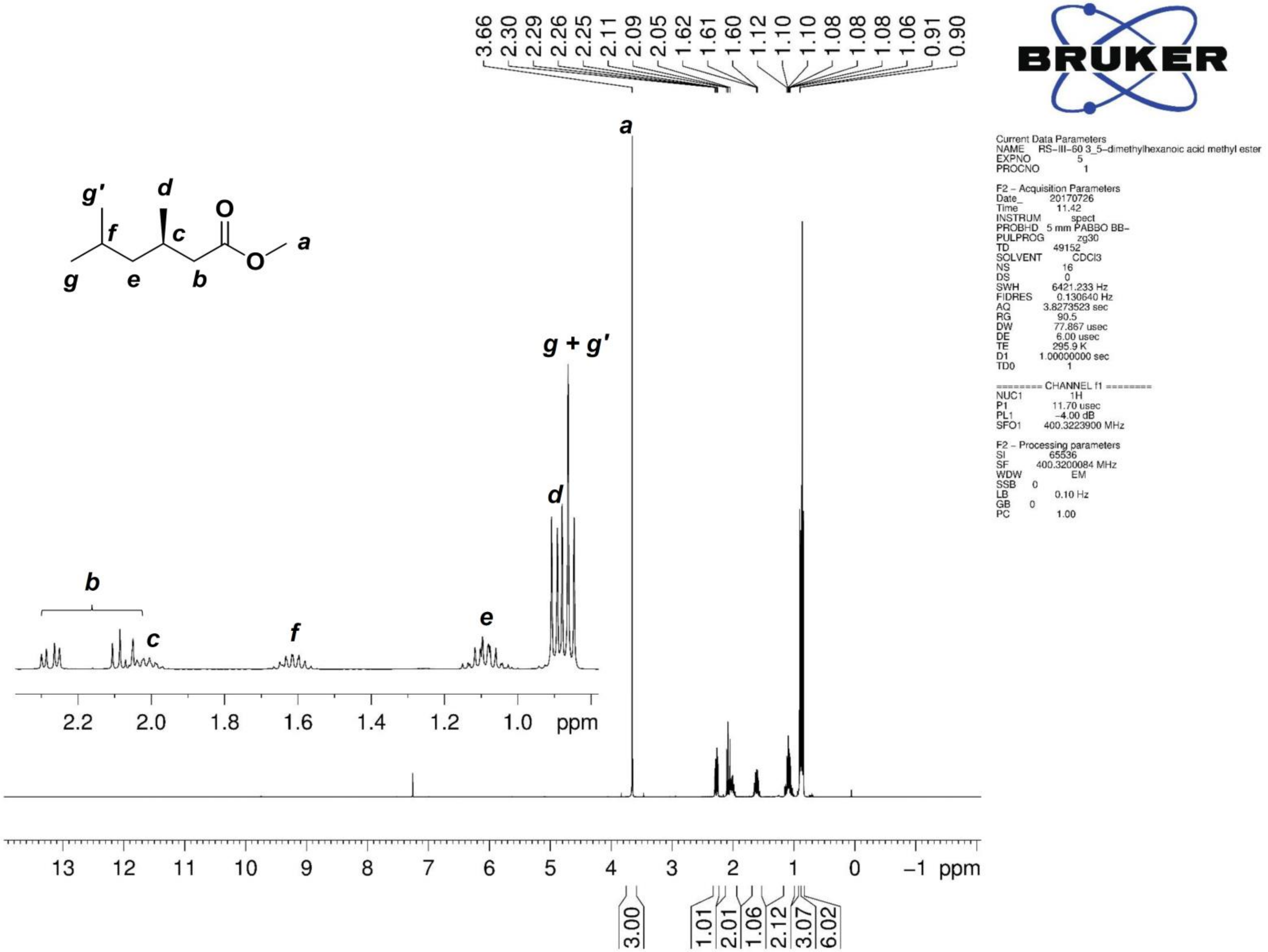
3.66 2.30 2.29 2.26 2.25 2.11 2.09 2.05 1.62 1.61 1.60 1.12 1.10 1.10 1.08 1.08 1.08 1.06 0.91 0.90
BRUKER
Current Data Parameters
NAME RS–III–60 3_5–dimethylhexanoic acid methyl ester
EXPNO 5
PROCNO 1
F2 – Acquisition Parameters
Date_ 20170726
Time 11.42
INSTRUM spect
PROBHD 5 mm PABBO BB–
PULPROG zg30
TD 49152
SOLVENT CDCl3
NS 16
DS 0
SWH 6421.233 Hz
FIDRES 0.130640 Hz
AQ 3.8273523 sec
RG 90.5
DW 77.867 usec
DE 6.00 usec
TE 295.9 K
D1 1.00000000 sec
TD0 1
======== CHANNEL f1 ========
NUC1 1H
P1 11.70 usec
PL1 –4.00 dB
SFO1 400.3223900 MHz
F2 – Processing parameters
SI 65536
SF 400.3200084 MHz
WDW EM
SSB 0
LB 0.10 Hz
GB 0
PC 1.00
a
g + g'
d
b
c
f
e
g' d O f c a O g e b
2.2 2.0 1.8 1.6 1.4 1.2 1.0 ppm
13 12 11 10 9 8 7 6 5 4 3 2 1 0 –1 ppm
3.00 1.01 2.01 1.06 2.12 3.07 6.02

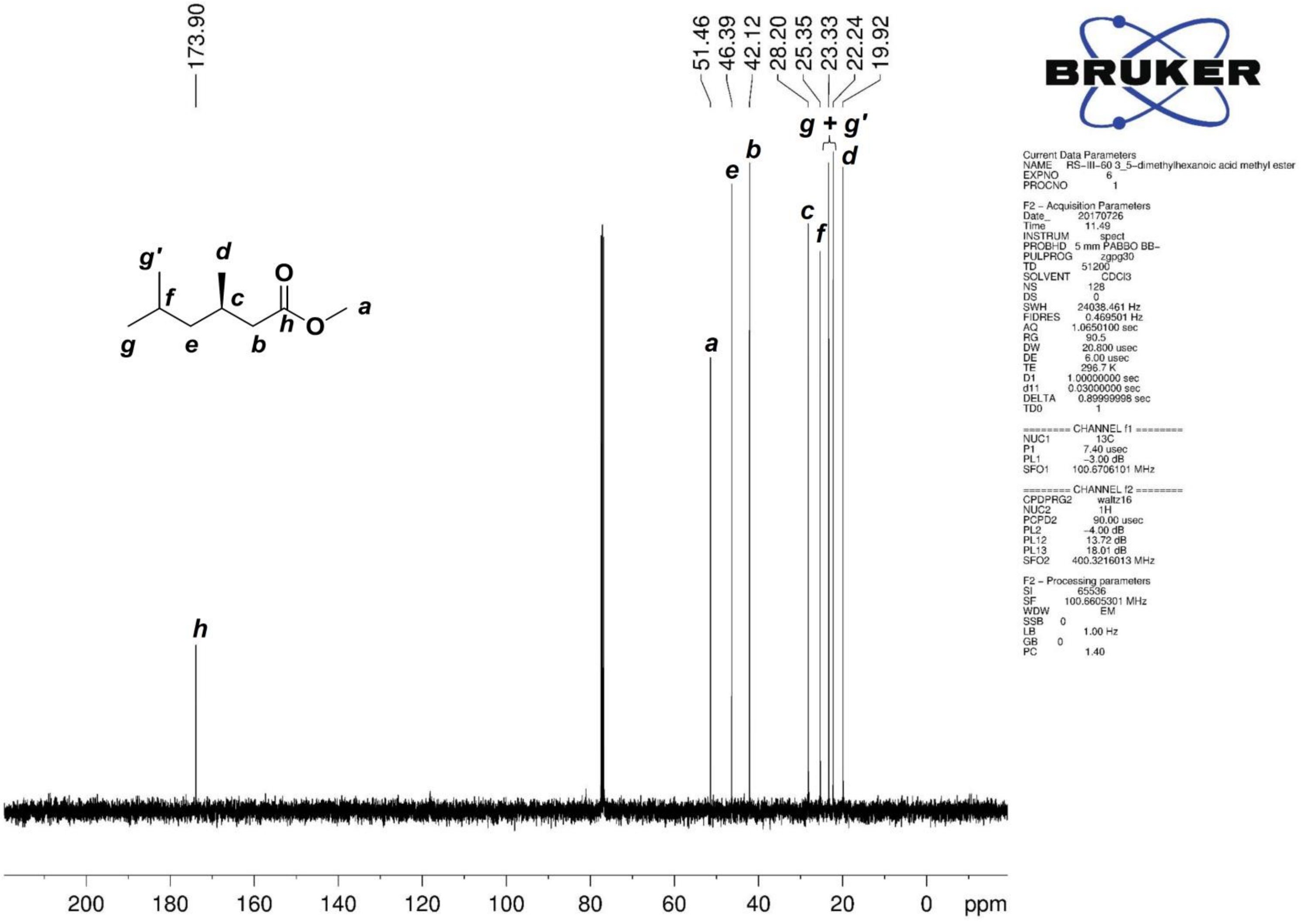
173.90
51.46
46.39
42.12
28.20
25.35
23.33
22.24
19.92
g + g'
b
e
d
c
f
a
h
g'
d
f
c
O
h
O
a
g
e
b
BRUKER
Current Data Parameters
NAME RS-III-60 3_5-dimethylhexanoic acid methyl ester
EXPNO 6
PROCNO 1
F2 - Acquisition Parameters
Date_ 20170726
Time 11.49
INSTRUM spect
PROBHD 5 mm PABBO BB-
PULPROG zgpg30
TD 51200
SOLVENT CDCl3
NS 128
DS 0
SWH 24038.461 Hz
FIDRES 0.469501 Hz
AQ 1.0650100 sec
RG 90.5
DW 20.800 usec
DE 6.00 usec
TE 296.7 K
D1 1.00000000 sec
d11 0.03000000 sec
DELTA 0.89999998 sec
TD0 1
======== CHANNEL f1 ========
NUC1 13C
P1 7.40 usec
PL1 -3.00 dB
SFO1 100.6706101 MHz
======== CHANNEL f2 ========
CPDPRG2 waltz16
NUC2 1H
PCPD2 90.00 usec
PL2 -4.00 dB
PL12 13.72 dB
PL13 18.01 dB
SFO2 400.3216013 MHz
F2 - Processing parameters
SI 65536
SF 100.6605301 MHz
WDW EM
SSB 0
LB 1.00 Hz
GB 0
PC 1.40
200
180
160
140
120
100
80
60
40
20
0
ppm

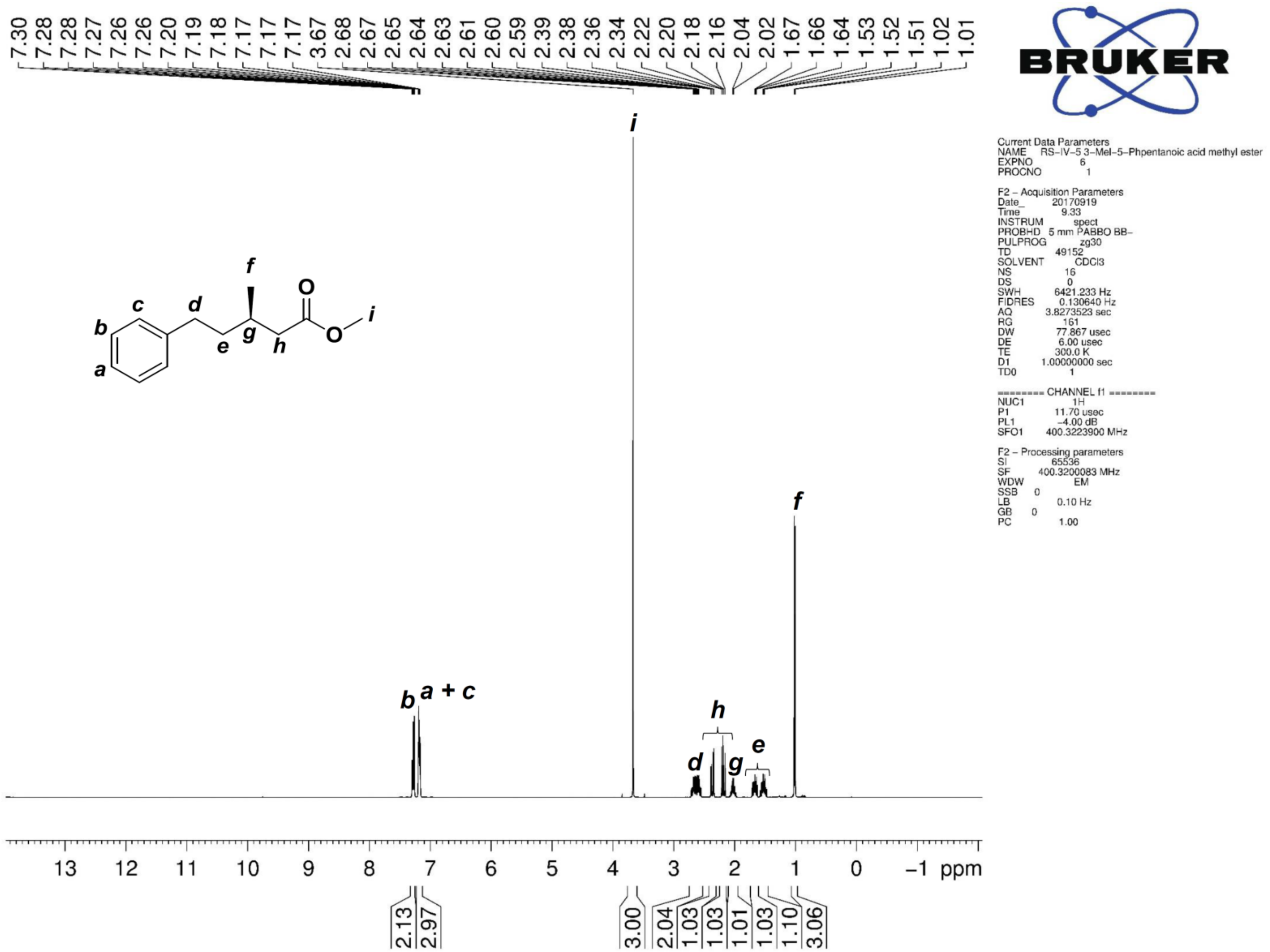
7.30 7.28 7.28 7.27 7.26 7.26 7.20 7.19 7.18 7.17 7.17 7.17 3.67 2.68 2.67 2.65 2.64 2.63 2.61 2.60 2.59 2.39 2.38 2.36 2.34 2.22 2.20 2.18 2.16 2.04 2.02 1.67 1.66 1.64 1.53 1.52 1.51 1.02 1.01
BRUKER
Current Data Parameters
NAME RS-IV-5 3-Mel-5-Phpentanoic acid methyl ester
EXPNO 6
PROCNO 1
F2 – Acquisition Parameters
Date_ 20170919
Time 9.33
INSTRUM spect
PROBHD 5 mm PABBO BB-
PULPROG zg30
TD 49152
SOLVENT CDCl3
NS 16
DS 0
SWH 6421.233 Hz
FIDRES 0.130640 Hz
AQ 3.8273523 sec
RG 161
DW 77.867 usec
DE 6.00 usec
TE 300.0 K
D1 1.00000000 sec
TD0 1
======== CHANNEL f1 ========
NUC1 1H
P1 11.70 usec
PL1 -4.00 dB
SFO1 400.3223900 MHz
F2 – Processing parameters
SI 65536
SF 400.3200083 MHz
WDW EM
SSB 0
LB 0.10 Hz
GB 0
PC 1.00
a b c d e f g h i O
b a + c
i
h
d g e
f
13 12 11 10 9 8 7 6 5 4 3 2 1 0 –1 ppm
2.13 2.97 3.00 2.04 1.03 1.03 1.01 1.03 1.10 3.06

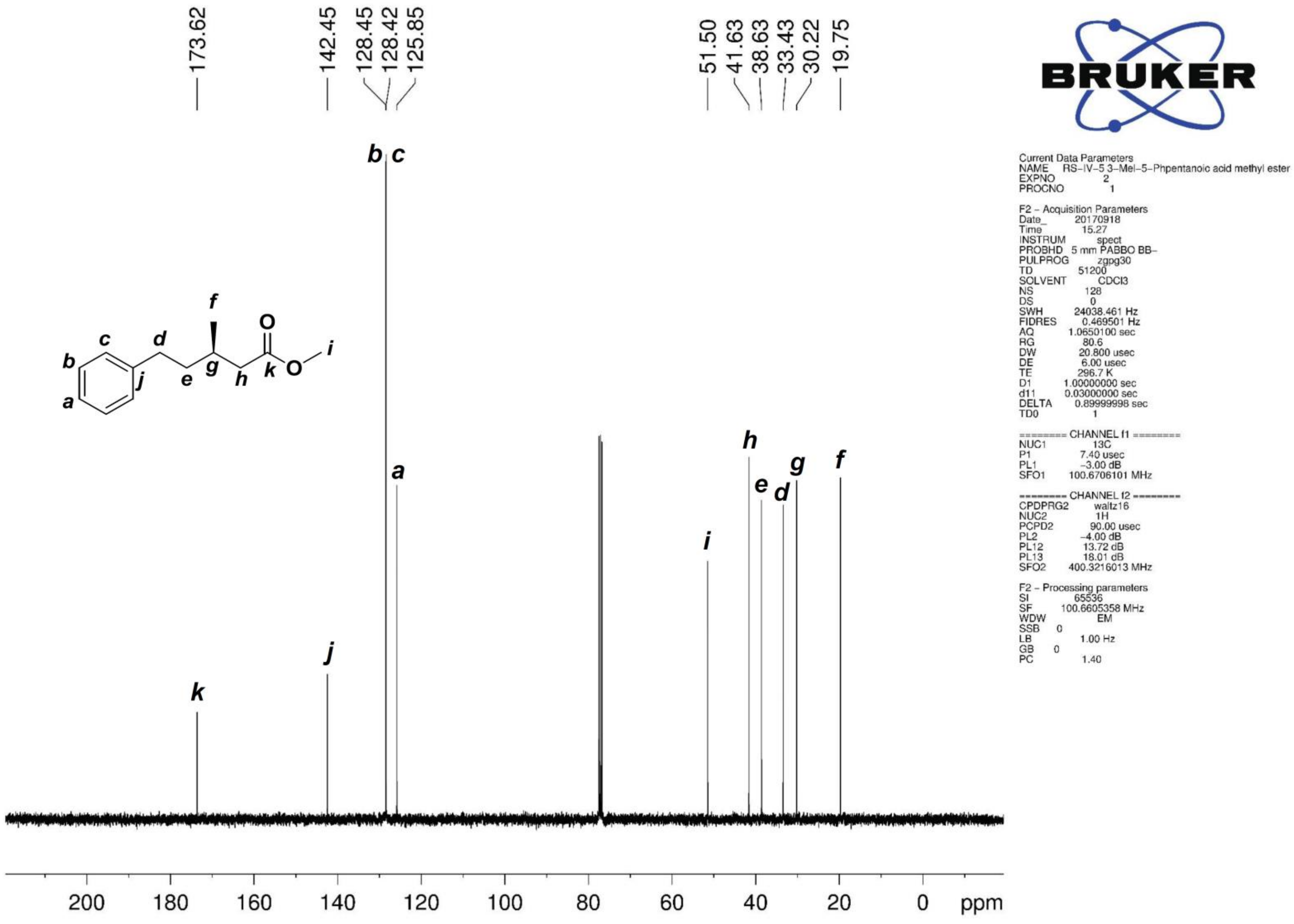
173.62
142.45
128.45
128.42
125.85
51.50
41.63
38.63
33.43
30.22
19.75
b c
a
k
j
i
h
e
d
g
f
O
O
200 180 160 140 120 100 80 60 40 20 0 ppm
Current Data Parameters
NAME RS-IV-5 3-MeI-5-Phpentanoic acid methyl ester
EXPNO 2
PROCNO 1
F2 - Acquisition Parameters
Date_ 20170918
Time 15.27
INSTRUM spect
PROBHD 5 mm PABBO BB-
PULPROG zgpg30
TD 51200
SOLVENT CDCl3
NS 128
DS 0
SWH 24038.461 Hz
FIDRES 0.469501 Hz
AQ 1.0650100 sec
RG 80.6
DW 20.800 usec
DE 6.00 usec
TE 296.7 K
D1 1.00000000 sec
d11 0.03000000 sec
DELTA 0.89999998 sec
TD0 1
======== CHANNEL f1 ========
NUC1 13C
P1 7.40 usec
PL1 -3.00 dB
SFO1 100.6706101 MHz
======== CHANNEL f2 ========
CPDPRG2 waltz16
NUC2 1H
PCPD2 90.00 usec
PL2 -4.00 dB
PL12 13.72 dB
PL13 18.01 dB
SFO2 400.3216013 MHz
F2 - Processing parameters
SI 65536
SF 100.6605358 MHz
WDW EM
SSB 0
LB 1.00 Hz
GB 0
PC 1.40

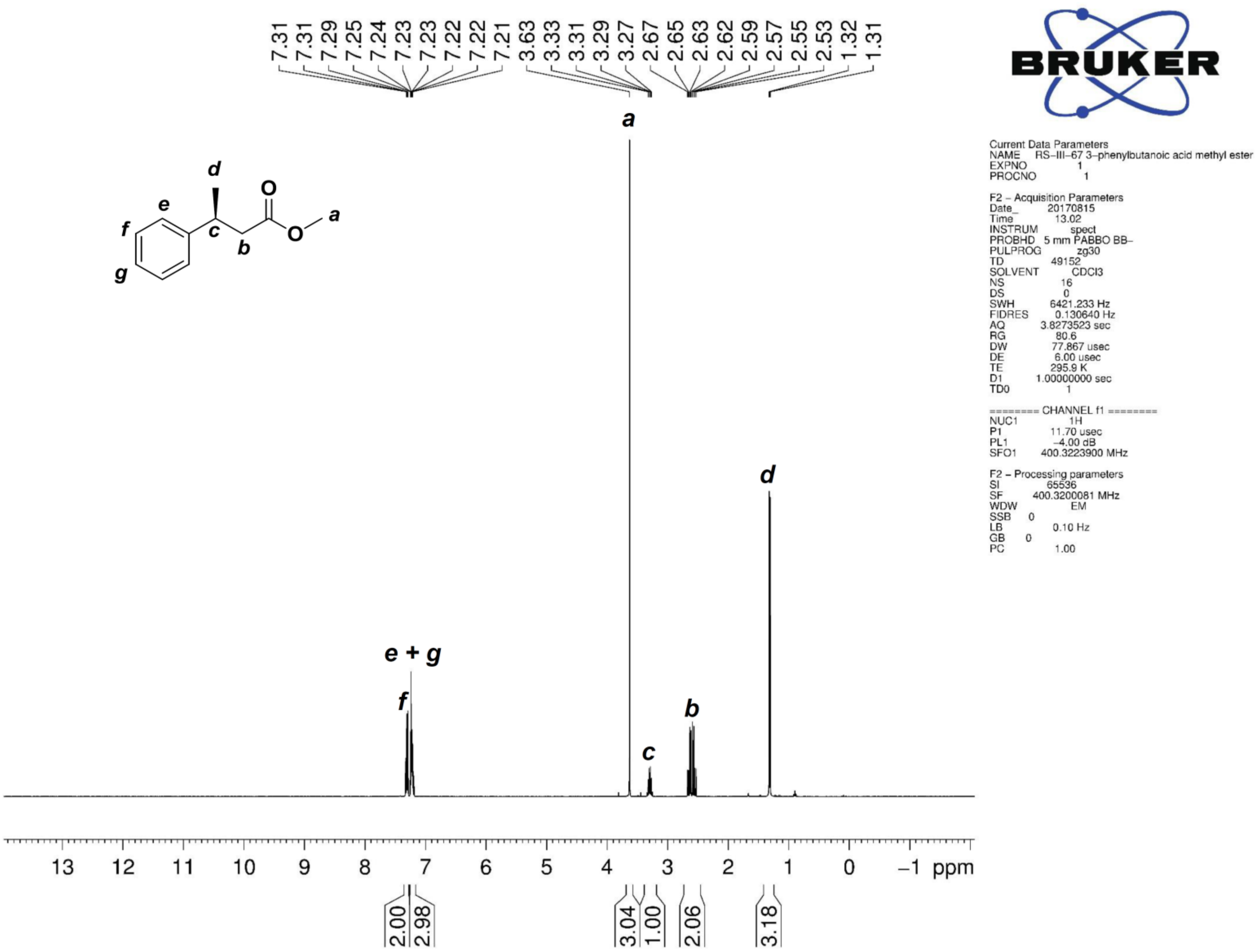
7.31 7.31 7.29 7.25 7.24 7.23 7.23 7.22 7.22 7.21 3.63 3.33 3.31 3.29 3.27 2.67 2.65 2.63 2.62 2.59 2.57 2.55 2.53 1.32 1.31
a
d
e
f
g
c
b
O
e + g
BRUKER
Current Data Parameters
NAME RS–III–67 3–phenylbutanoic acid methyl ester
EXPNO 1
PROCNO 1
F2 – Acquisition Parameters
Date_ 20170815
Time 13.02
INSTRUM spect
PROBHD 5 mm PABBO BB–
PULPROG zg30
TD 49152
SOLVENT CDCl3
NS 16
DS 0
SWH 6421.233 Hz
FIDRES 0.130640 Hz
AQ 3.8273523 sec
RG 80.6
DW 77.867 usec
DE 6.00 usec
TE 295.9 K
D1 1.00000000 sec
TD0 1
======== CHANNEL f1 ========
NUC1 1H
P1 11.70 usec
PL1 –4.00 dB
SFO1 400.3223900 MHz
F2 – Processing parameters
SI 65536
SF 400.3200081 MHz
WDW EM
SSB 0
LB 0.10 Hz
GB 0
PC 1.00
13 12 11 10 9 8 7 6 5 4 3 2 1 0 –1 ppm
2.00 2.98 3.04 1.00 2.06 3.18

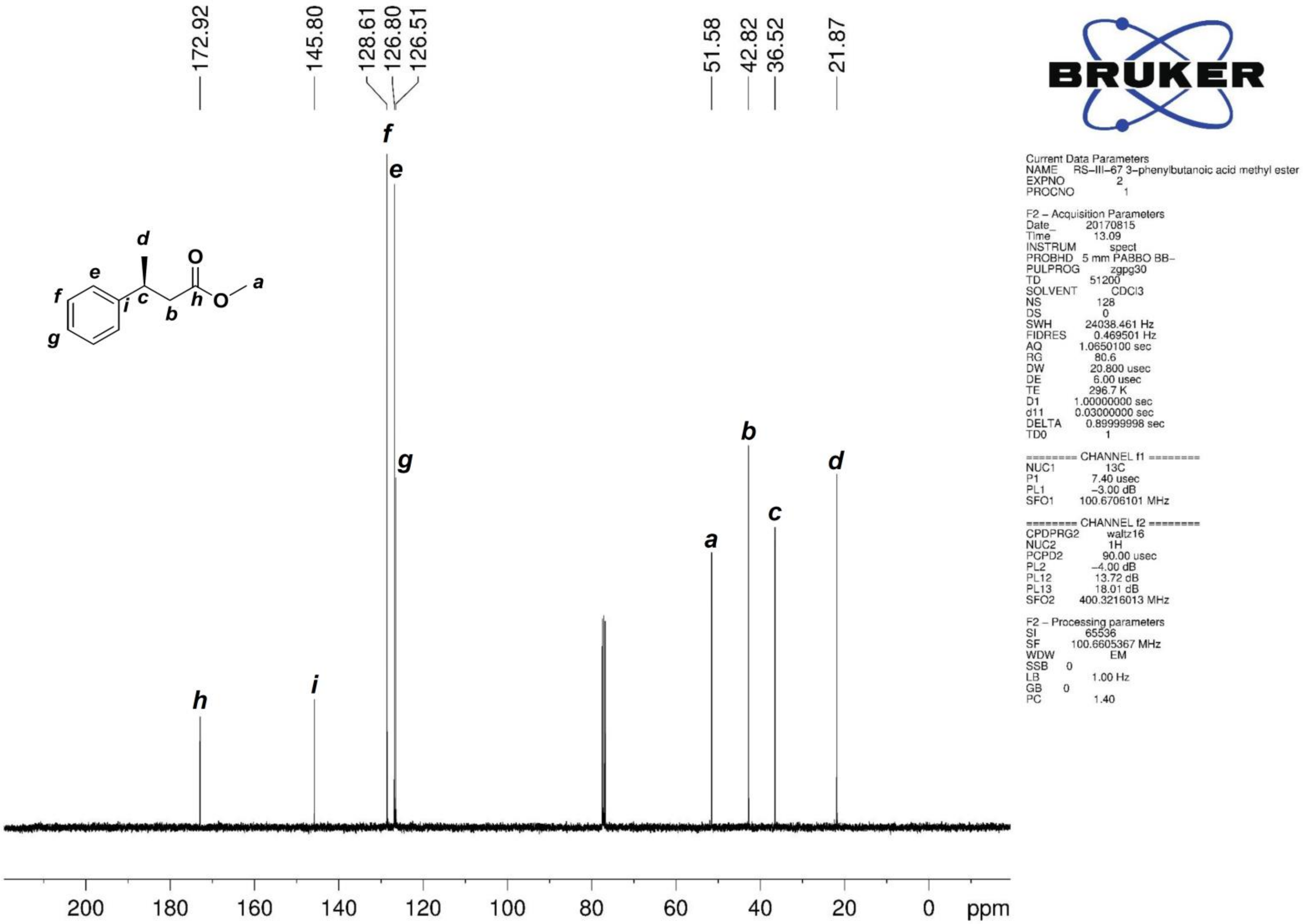
172.92
145.80
128.61
126.80
126.51
51.58
42.82
36.52
21.87
h
i
f
e
g
a
b
c
d
O
BRUKER
Current Data Parameters
NAME RS–III–67 3–phenylbutanoic acid methyl ester
EXPNO 2
PROCNO 1
F2 – Acquisition Parameters
Date_ 20170815
Time 13.09
INSTRUM spect
PROBHD 5 mm PABBO BB–
PULPROG zgpg30
TD 51200
SOLVENT CDCl3
NS 128
DS 0
SWH 24038.461 Hz
FIDRES 0.469501 Hz
AQ 1.0650100 sec
RG 80.6
DW 20.800 usec
DE 6.00 usec
TE 296.7 K
D1 1.00000000 sec
d11 0.03000000 sec
DELTA 0.89999998 sec
TD0 1
======== CHANNEL f1 ========
NUC1 13C
P1 7.40 usec
PL1 –3.00 dB
SFO1 100.6706101 MHz
======== CHANNEL f2 ========
CPDPRG2 waltz16
NUC2 1H
PCPD2 90.00 usec
PL2 –4.00 dB
PL12 13.72 dB
PL13 18.01 dB
SFO2 400.3216013 MHz
F2 – Processing parameters
SI 65536
SF 100.6605367 MHz
WDW EM
SSB 0
LB 1.00 Hz
GB 0
PC 1.40
200
180
160
140
120
100
80
60
40
20
0
ppm

7 Example GC traces of diastereomers following hydrogenation

```
File       :D:\MassHunter\GCMS\1\data\Ryan S\FID\RS-III-59\isothermal\hy
...         drog 1.D
Operator   :
Instrument :    5975B GCMS
Acquired   : 13 Jul 2017  11:14     using AcqMethod isotherm 250.M
Sample Name:
Misc Info  :
```

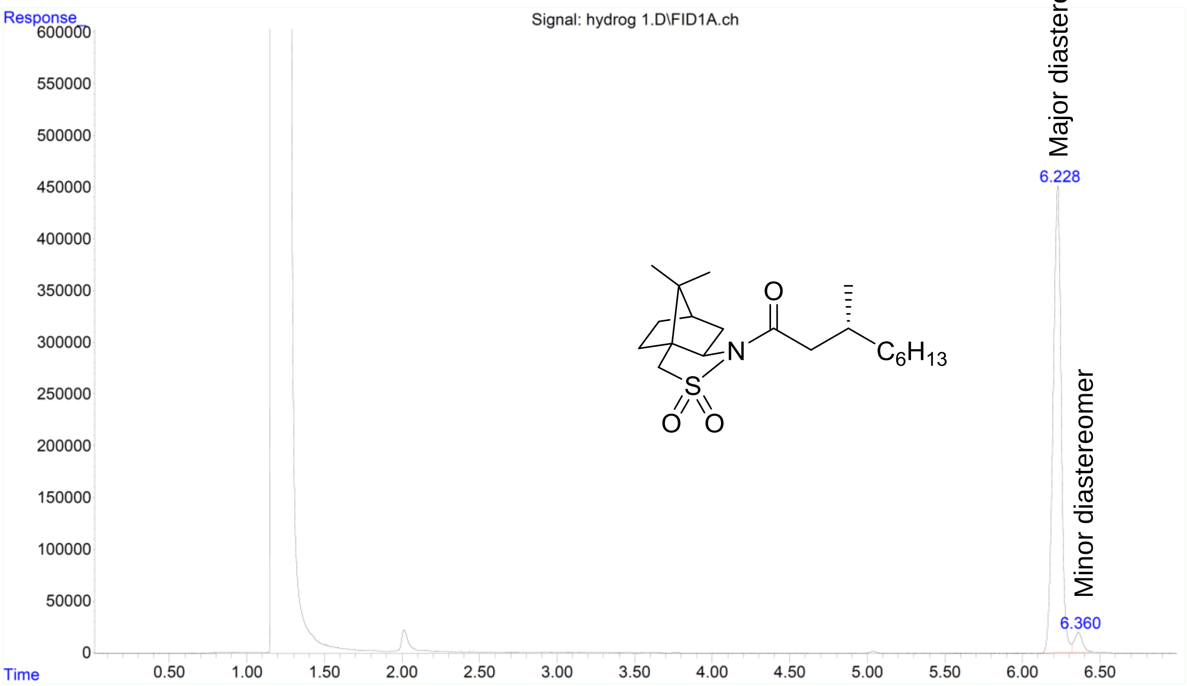

Area Percent Report

```
Data Path : D:\MassHunter\GCMS\1\data\Ryan S\FID\RS-III-59\isothermal\
Data File : hydrog 1.D
Signal(s) : FID1A.ch
Acq On    : 13 Jul 2017  11:14
Sample    :
Misc      :
ALS Vial  : 3   Sample Multiplier: 1

Integration File: autoint1.e

Method    : D:\MassHunter\GCMS\1\methods\MS.m
Title     :

Signal    : FID1A.ch
```

peak #	R.T. min	Start min	End min	PK TY	peak height	peak area	peak % max.	% of total
1	6.228	6.115	6.317	BV	451761	16336302	100.00%	95.861%
2	6.360	6.317	6.447	VB	19178	705294	4.32%	4.139%
			Sum of corrected areas:				17041596	

```
File       :D:\MassHunter\GCMS\1\data\Ryan S\FID\RS-III-60\isothermal\hy
...         dog 1.D
Operator   :
Instrument :    5975B GCMS
Acquired   : 25 Jul 2017  12:33     using AcqMethod isotherm 225.M
Sample Name:
Misc Info  :
```

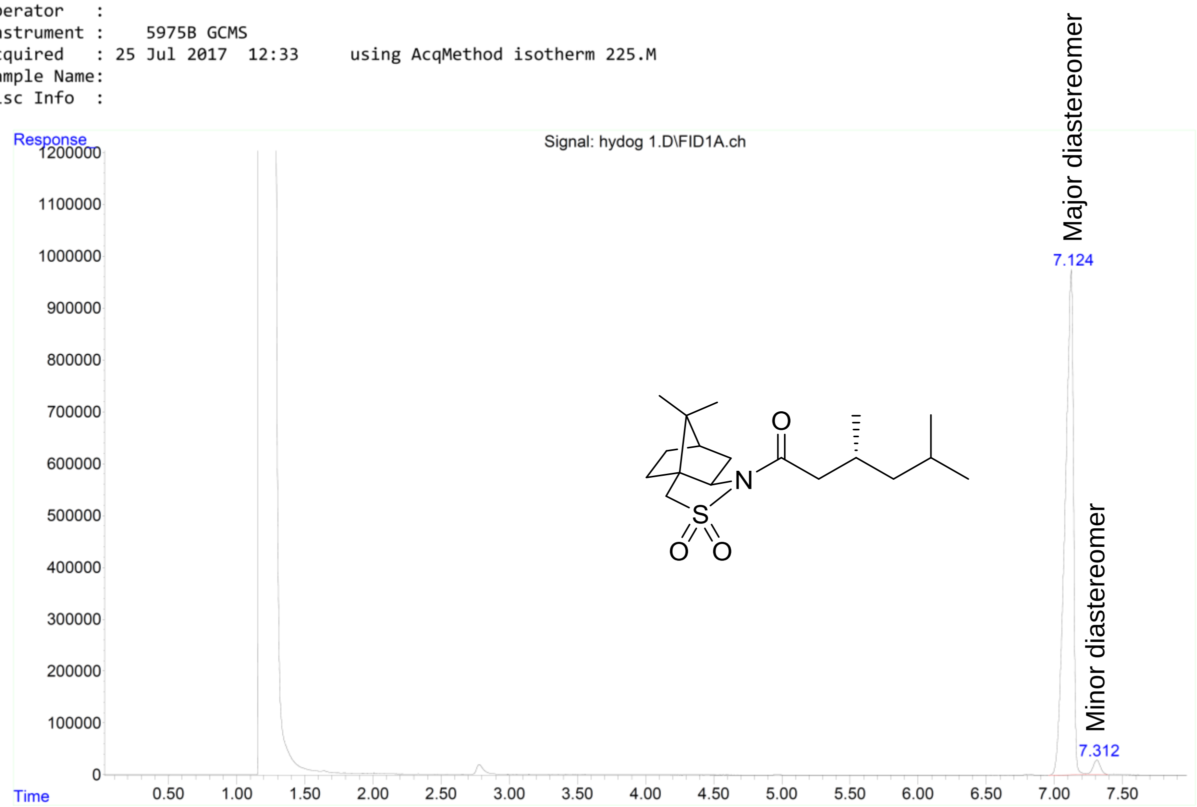


```
                              Area Percent Report

 Data Path : D:\MassHunter\GCMS\1\data\Ryan S\FID\RS-III-60\isothermal\
 Data File : hydog 1.D
 Signal(s) : FID1A.ch
 Acq On    : 25 Jul 2017  12:33
 Sample    :
 Misc      :
 ALS Vial  : 3   Sample Multiplier: 1

 Integration File: autoint1.e

 Method    : D:\MassHunter\GCMS\1\methods\MS.m
 Title     :

 Signal    : FID1A.ch
```

peak #	R.T. min	Start min	End min	PK TY	peak height	peak area	peak % max.	% of total
1	7.124	6.957	7.234	BV	974472	41865966	100.00%	97.548%
2	7.312	7.234	7.394	VB	27948	1052414	2.51%	2.452%

Sum of corrected areas: 42918380

File   :D:\MassHunter\GCMS\1\data\Ryan S\FID\RS-IV-8\isotherm\hydrog
...   1.D
Operator   :
Instrument :   5975B GCMS
Acquired   : 21 Sep 2017 11:10   using AcqMethod isotherm 275.M
Sample Name:
Misc Info :

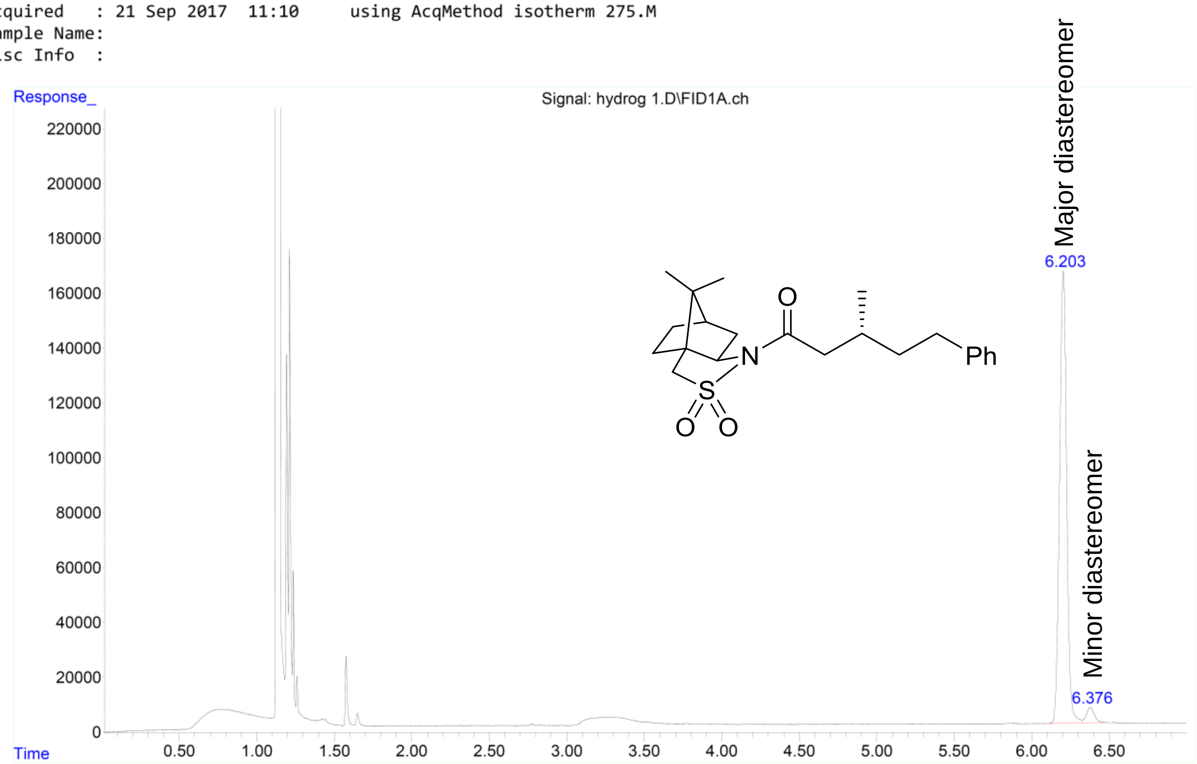

Area Percent Report

Data Path : D:\MassHunter\GCMS\1\data\Ryan S\FID\RS-IV-8\isotherm\
Data File : hydrog 1.D
Signal(s) : FID1A.ch
Acq On   : 21 Sep 2017 11:10
Sample   :
Misc   :
ALS Vial : 17   Sample Multiplier: 1

Integration File: autoint1.e

Method   : D:\MassHunter\GCMS\1\methods\MS.m
Title   :

Signal   : FID1A.ch

peak #	R.T. min	Start min	End min	PK TY	peak height	peak area	peak % max.	% of total
1	6.203	6.104	6.323	BV	164854	5201757	100.00%	96.258%
2	6.376	6.323	6.475	VB	5698	202215	3.89%	3.742%

Sum of corrected areas: 5403972

File   :D:\MassHunter\GCMS\1\data\Ryan S\FID\RS-III-64\hydrog 1.D
Operator   :
Acquired   : 31 Jul 2017 12:08   using AcqMethod FID_long.M
Instrument :   5975B GCMS
Sample Name:
Misc Info :
Vial Number: 6

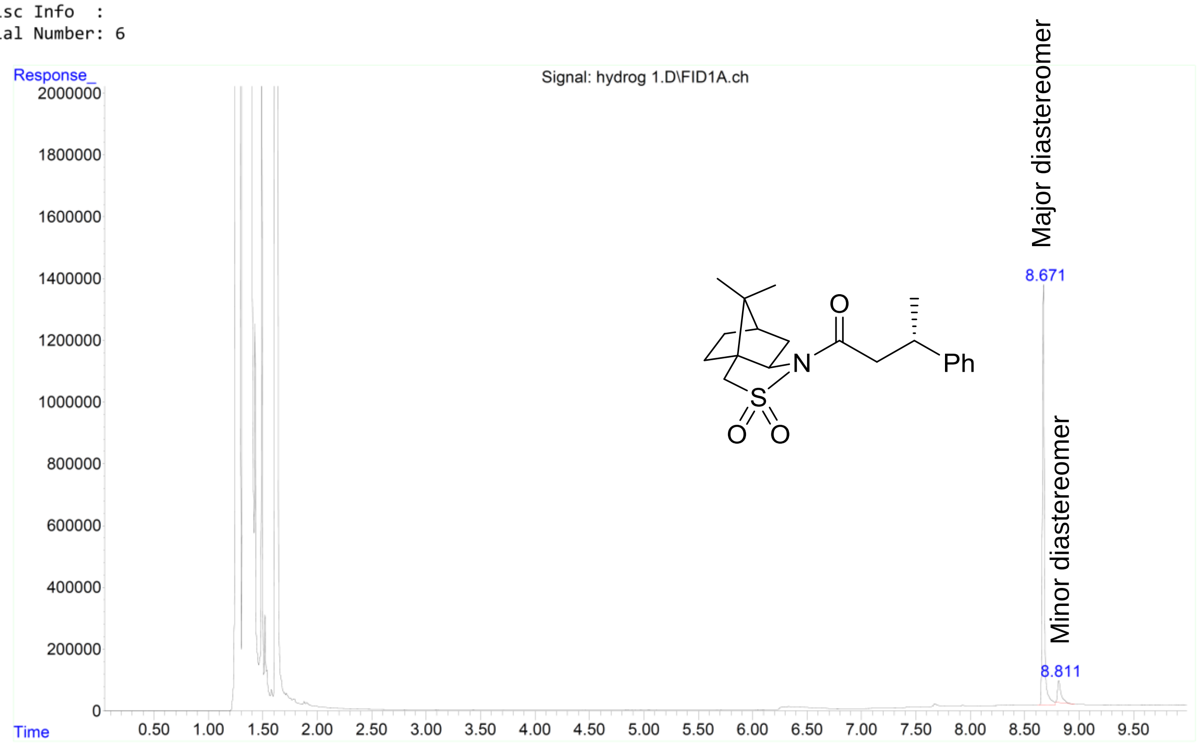

Area Percent Report

Data Path : D:\MassHunter\GCMS\1\data\Ryan S\FID\RS-III-64\
Data File : hydrog 1.D
Signal(s) : FID1A.ch
Acq On   : 31 Jul 2017 12:08
Sample   :
Misc   :
ALS Vial : 6   Sample Multiplier: 1

Integration File: autoint1.e

Method   : D:\MassHunter\GCMS\1\methods\MS.m
Title   :

Signal   : FID1A.ch

peak #	R.T. min	Start min	End min	PK TY	peak height	peak area	peak % max.	% of total
1	8.671	8.616	8.783	BV	1361159	17790937	100.00%	91.578%
2	8.811	8.784	8.961	M	71846	1636124	9.20%	8.422%

Sum of corrected areas: 19427061

8 Diastereoselective excess monitoring of hydrogenation

Diastereoselectivity over the hydrogenation stage was monitorred by withdrawing a ~25 μL aliquot from the post-hydrogenation organic phase and submitting to GC analysis every 30 min (once every nominal residence volume) during telescoped flow experiment. Plots of d.e. over time for each substrate are provided below:

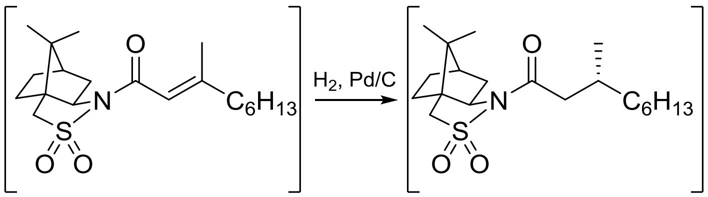

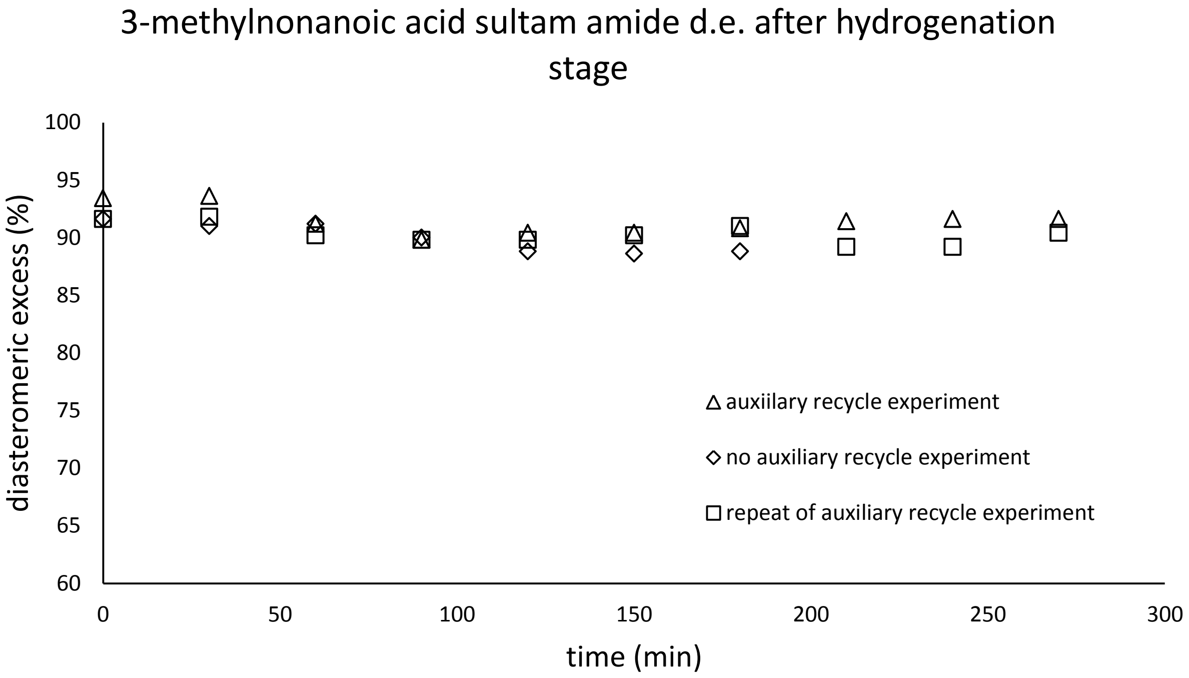

Figure S9. Diastereoselectivity over hydrogenation step of experiments with (*E*)-3-methylnon-2-eneoic acid chloride substrate

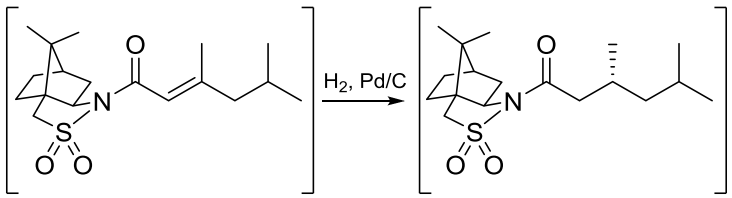

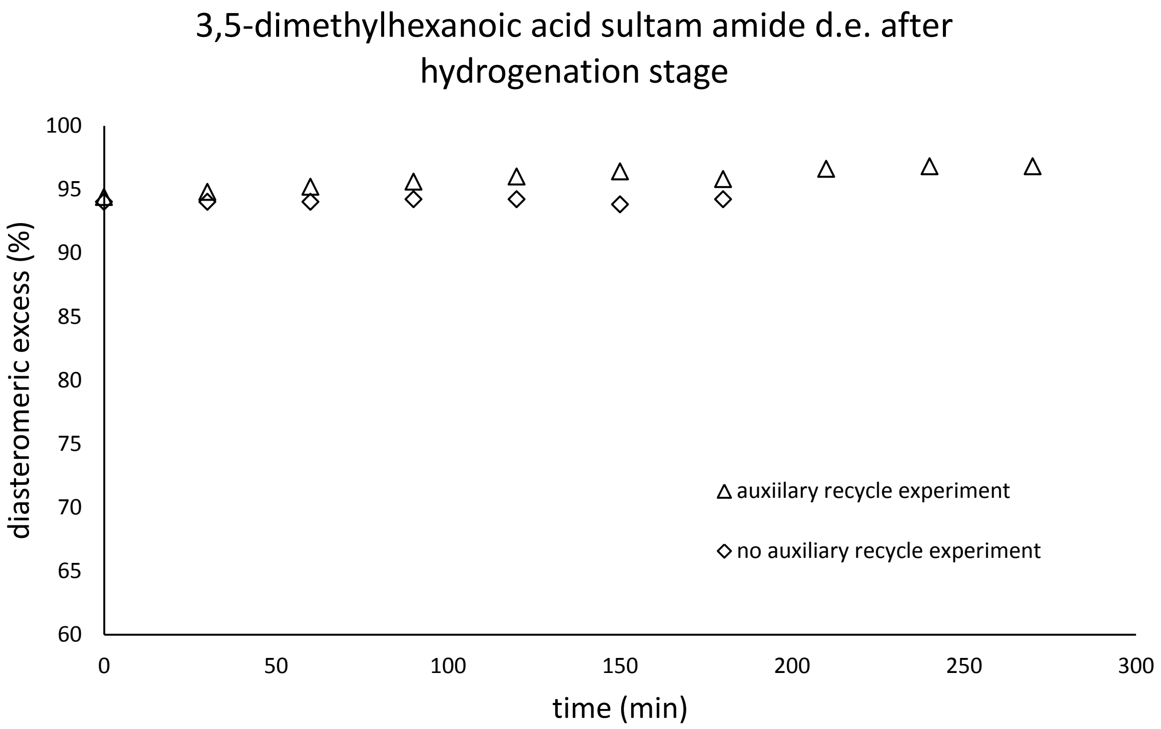

Figure S10. Diastereoselectivity over hydrogenation step of experiments with (*E*)-3,5-dimethylhex-2-eneoic acid chloride substrate

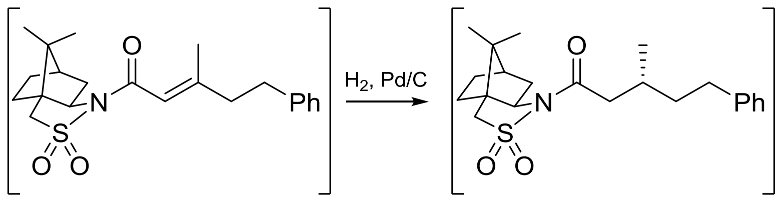

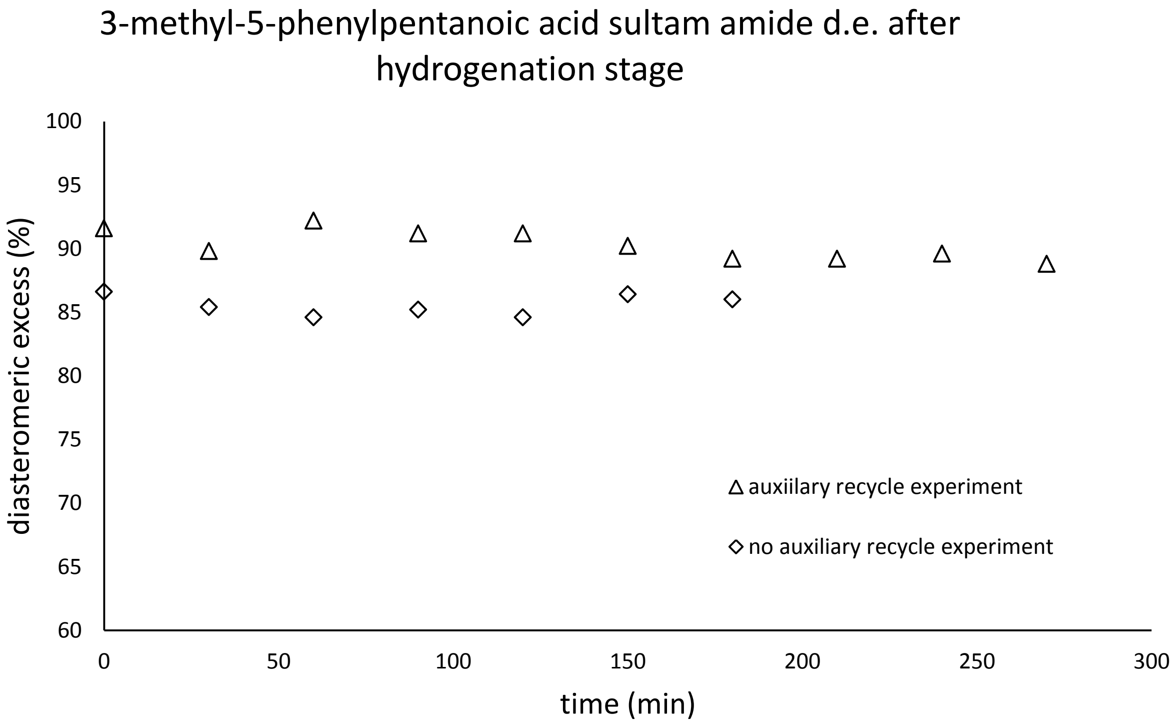

Figure S11. Diastereoselectivity over hydrogenation step of experiments with (*E*)-3-methyl-5-phenylpent-2-eneoic acid chloride substrate

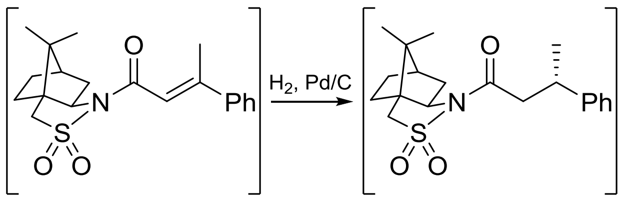

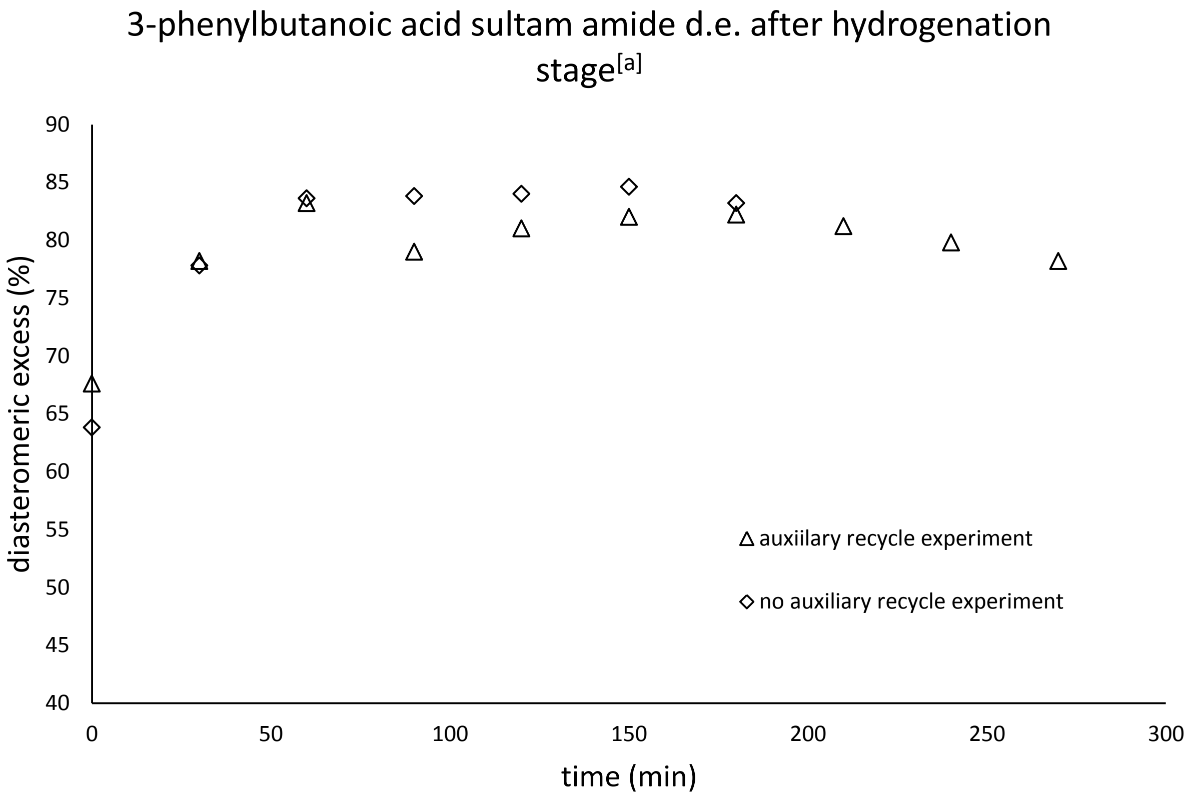

Figure S12. Diastereoselectivity over hydrogenation step of experiments with (*E*)-3-phenylbut-2-eneoic acid chloride substrate

[a] The d.r. of the 3,3-dialkyl substrates over the hydrogenation was steady from the first elution of product from the PBR, but the 3,3-aryl,alkyl substrate exhibited an initially lower d.r. of 80:20 that increased over the first 60 min of operation to 92:8 (auxiliary recovery experiment) or 90:10 (auxiliary recycle experiment) and then remained steady at this selectivity for the remainder of the experiment. This was attributed to chromatography effects within the PBR and represents a longer time to attain true steady-state operation with this substrate.

9 Chiral HPLC trace of (*R*)-3-Methyl-5-phenlybutanoic acid methyl ester

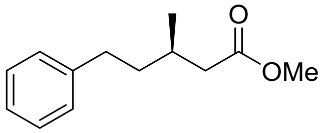


```
Data File C:\CHEM32\1\DATA\NEWMAN\RS OCT 05 17 2017-10-05 15-58-00\072-0401.D
Sample Name: RS3 Oct 05 17

=====================================================================
Acq. Operator   : Beauchemin                    Seq. Line :   4
Acq. Instrument : Instrument 1                   Location : Vial 72
Injection Date  : 10/5/2017 7:01:22 PM                Inj :   1
                                               Inj Volume : 5 µl
Acq. Method     : C:\Chem32\1\DATA\NEWMAN\RS OCT 05 17 2017-10-05 15-58-00\COL4-0_25PC-IPROH-
                  75MIN.M
Last changed    : 10/5/2017 3:56:24 PM by Beauchemin
Analysis Method : C:\CHEM32\1\DATA\NEWMAN\RS OCT 05 17 2017-10-05 15-58-00\072-0401.D\DA.M (
                  COL4-0_25PC-IPROH-75MIN.M)
Last changed    : 10/11/2017 5:00:43 PM by Beauchemin
                  (modified after loading)
```

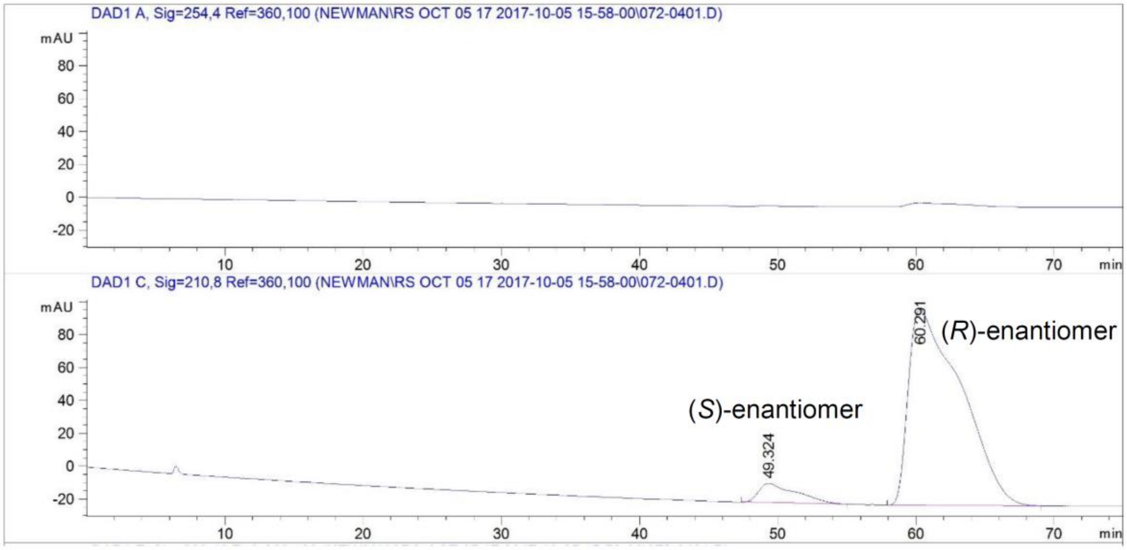


```
Data File C:\CHEM32\1\DATA\NEWMAN\RS OCT 05 17 2017-10-05 15-58-00\072-0401.D
Sample Name: RS3 Oct 05 17

=====================================================================
                         Area Percent Report
=====================================================================

Sorted By             :      Signal
Multiplier            :      1.0000
Dilution              :      1.0000
Use Multiplier & Dilution Factor with ISTDs


Signal 1: DAD1 A, Sig=254,4 Ref=360,100


Signal 2: DAD1 C, Sig=210,8 Ref=360,100

Peak RetTime Type  Width     Area       Height     Area
  #   [min]        [min]   [mAU*s]      [mAU]        %
----|-------|----|-------|----------|----------|--------|
   1  49.324 BV     2.5198 2238.77808   11.83050   6.6108
   2  60.291 BB     3.5475 3.16264e4   119.34978  93.3892

Totals :                   3.38652e4   131.18029
```

References

1. Y. Wang and M. Gu, *Anal. Chem.*, 2010, **82**, 7055–7062.
2. M. O'Brien, P. Koos, D. L. Browne and S. V. Ley, *Org. Biomol. Chem.*, 2012, **10**, 7031–7036.
3. R. K. Jensen, N. Thykier, M. V. Enevoldsen and A. T. Lindhardt, *Org. Process Res. Dev.*, 2017, **21**, 370–376.
4. R. J. Ingham, C. Battilocchio, D. E. Fitzpatrick, E. Sliwinski, J. M. Hawkins and S. V. Ley, *Angew. Chem. Int. Ed.*, 2015, **54**, 144–148.
5. K. Ogura, T. Nishino, T. Koyama and S. Seto, *J. Am. Chem. Soc.*, 1970, **92**, 6036–6041.
6. M. Takimoto and Z. Hou, *Chem. Eur. J.*, 2013, **19**, 11439–11445.
7. M. Bellassoued, S. Mouelhi, P. Fromentin and A. Gonzalez, *J. Organomet. Chem.*, 2005, **690**, 2172–2179.
8. Q. Yan, D. Kong, W. Zhao, G. Zi and G. Hou, *J. Org. Chem.*, 2016, **81**, 2070–2077.
9. D. H. Woodmansee, M.-A. Muller, M. Neuburger and A. Pfaltz, *Chem. Sci.*, 2010, **1**, 72–78.
10. K. Ando and K. Yamada, *Green Chem.*, 2011, **13**, 1143–1146.
11. P. S. Tiseni and R. Peters, *Angew. Chem. Int. Ed.*, 2007, **46**, 5325–5328.
12. D. Achard, S. Grisoni, J. L. Malleron, J. F. Peyronel and M. Tabart, U.S. Patent 5463077, 1995.
13. E. V. Dehmlow, R. Thieser, Y. Sasson and E. Pross, *Tetrahedron*, 1985, **41**, 2927–2932.
14. T. Suzuki, J. Ozaki and R. Sugawara, *Agric. Biol. Chem.*, 1983, **47**, 869–875.
15. B. M. Trost, J. T. Masters, B. R. Taft and J.-P. Lumb, *Chem. Sci.*, 2016, **7**, 6217–6231.
16. F. López, S. R. Harutyunyan, A. Meetsma, A. J. Minnaard and B. L. Feringa, *Angew. Chem. Int. Ed.*, 2005, **44**, 2752–2756.
17. C.-J. Hou, W.-L. Guo, X.-P. Hu, J. Deng and Z. Zheng, *Tetrahedron: Asymmetry*, 2011, **22**, 195–199.
